# Ligand Engineering for Precise Control of Ultrathin CsPbI_3_ Nanoplatelet Superlattices for Efficient Light‐Emitting Diodes

**DOI:** 10.1002/adma.74023

**Published:** 2026-07-10

**Authors:** Jongbeom Kim, Woo Hyeon Jeong, Junzhi Ye, Allison Nicole Arber, Vikram, Donghan Kim, Yi‐Teng Huang, Yixin Wang, Dongeun Kim, Dongryeol Lee, Chia‐Yu Chang, Xinyu Shen, Sung Yong Bae, Ashish Gaurav, Ye In Kim, Jinkyu Yang, Boseung Je, Changsoon Cho, Akshay Rao, Henry J. Snaith, M. Saiful Islam, Bo Ram Lee, Myoung Hoon Song, Robert L. Z. Hoye

**Affiliations:** ^1^ Department of Materials Science and Engineering Ulsan National Institute of Science and Technology (UNIST) Ulsan Republic of Korea; ^2^ School of Advanced Materials Science and Engineering Sungkyunkwan University Suwon Republic of Korea; ^3^ Inorganic Chemistry Laboratory University of Oxford Oxford UK; ^4^ Institute of Polymer Optoelectronic Materials and Devices Guangdong Basic Research Center of Excellence for Energy & Information Polymer Materials State Key Laboratory of Luminescent Materials and Devices School of Materials Science and Engineering South China University of Technology Guangzhou China; ^5^ Department of Materials University of Oxford Oxford UK; ^6^ Graduate Institute of Photonics and Optoelectronics and Department of Electrical Engineering National Taiwan University Taipei Taiwan; ^7^ Clarendon Laboratory Department of Physics University of Oxford Oxford United Kingdom; ^8^ Department of Materials Science and Engineering Pohang University of Science and Technology (POSTECH) Pohang Republic of Korea; ^9^ Cavendish Laboratory University of Cambridge Cambridge UK

**Keywords:** light‐emitting diode, linearly polarized light emission, nanocrystal growth control, perovskite nanoplatelets, superlattices

## Abstract

Strongly‐confined perovskite nanoplatelets (PeNPLs) offer opportunities not found in conventional isotropic nanocubes, especially in producing linearly polarized light, as well as enhancing outcoupling through control over the transition dipole moment. But this requires ultrathin nanoplatelets with three or fewer monolayers of PbI_6_ octahedra across the thickness, which are challenging to synthesise uniformly, and their luminescence is strongly affected by surface defects. Together, these limit the performance of ultrathin PeNPLs in light‐emitting diodes (LEDs). Here, we address these challenges with an ancillary ligand engineering strategy. We demonstrate that ligands with phosphoryl functional groups strongly bind to the perovskite surface, while having an organic backbone that is not sterically bulky ensures high ligand density. By modulating nucleation and growth, these ancillary ligands lead to monodisperse PeNPLs that stack more uniformly when self‐assembled into superlattices, with suppressed agglomeration. As a result, from edge‐up PeNPL superlattices, we achieve an enhanced degree of polarization, while from face‐down PeNPL superlattices, we achieve enhanced outcoupling that results in LEDs with 13.1% external quantum efficiency, the highest reported for ultrathin PeNPL LEDs. This work establishes ancillary ligand‐induced synthesis as a decisive route to achieve uniform nanoplatelets with robust orientation control, enabling full utilization of the multifunctionality of anisotropic PeNPLs.

## Graphical abstract

In this work, we introduce ancillary‐ligand‐mediated growth control of ultrathin CsPbI_3_ PeNPLs to achieve highly uniform thickness and reduced agglomeration. Strong ancillary‐ligand coordination to the perovskite surface regulates nucleation and improves surface passivation, enabling color‐pure emission and preferentially oriented superlattices. As a result, LEDs with 13.1% EQE are realized, the highest for ultrathin PeNPLs.

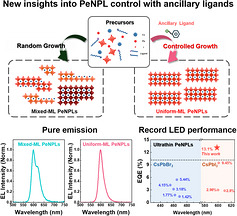

## 1 Introduction

Colloidal semiconductor nanocrystals (NCs) offer advantages for optics and photonics, as well as for fundamental investigations of light‐matter properties [1, 2, 3]. To fully realize these benefits, it is critical to achieve highly monodisperse NCs, which requires precise control over nucleation and growth via well‐regulated reaction conditions [4, 5, 6, 7, 8]. Lead halide perovskites, with the general formula APbX_3_ (A = formamidinium, methylammonium, Cs; X = Cl, Br, I), have emerged as one of the most promising NC materials, due to their narrow full‐width at half‐maximum (FWHM), defect tolerance, and the ability to achieve high luminescence quantum yields with cost‐effective solution processing methods [9, 10, 11, 12, 13]. In particular, perovskite nanoplatelets (PeNPLs), classified by the number of monolayers (MLs) of lead‐halide octahedra across the thickness, can have their emission wavelength tuned not only by changing their composition, but also by tuning their thickness. Thinner PeNPLs exhibit a blue‐shift in their emission due to stronger quantum and dielectric confinement effects across the out‐of‐plane direction [14, 15]. Furthermore, the anisotropy in their confinement leads to exciton fine structure splitting, which can enable linearly polarized light emission [16, 17, 18].

Prior studies have shown that the pronounced linearly polarized emission from PeNPLs is strongly thickness‐dependent and most reliably obtained in the ultrathin regime (typically *n* ≤ 3, where *n* is the number of monolayers of Pb‐halide octahedra across the thickness of the PeNPL). In ultrathin PeNPLs, anisotropic confinement maximizes the fine structure splitting in the bright excitonic manifold, where the excitonic state associated with the out‐of‐plane direction is raised in energy more than the doubly degenerate in‐plane excitonic states [18, 19, 20]. Thicker nanoplatelets generally exhibit a weaker polarization response due to reduced exciton fine structure splitting. Beyond the intrinsic excitonic effects, the pronounced shape anisotropy of ultrathin PeNPLs can also promote orientational ordering and superlattice assembly, allowing control of the transition dipole moment in films [21]. Therefore, achieving strong linear polarization and enabling ordered assembly requires precise and uniform thickness control in the ultrathin regime, since even a small fraction of non‐uniformity by having a mixture of different ML PeNPLs can rapidly reduce the multifunctionality of the assembled films. However, attaining this uniformity among nanoplatelets is highly challenging, given their surface‐dominated nature. The high surface area‐to‐volume ratio amplifies sensitivity to ligand dynamics and surface defects, thereby requiring robust surface passivation.

PeNPLs present inherent challenges due to their fast reaction kinetics, which stems from their ionic nature and low formation energy, making it difficult to achieve nanoplatelets that are uniform in thickness [5, 22, 23]. This results in a distribution of emission wavelengths that compromise their optical properties, including their color saturation (required for ultrahigh definition displays) and degree of linear polarization (required for optical communications, 3D displays, and bioimaging) [24, 25]. Such limitations represent a fundamental bottleneck across all classes of NPLs, not just halide perovskites, since achieving colloidal uniformity and structural stability are essential for fully exploiting their optoelectronic potential [26].

For the synthesis and dispersion of PeNPLs, long chain organic ligands such as oleate (OA–) and oleylammonium (OAm^+^) are commonly used [27, 28, 29]. While these native ligands play a crucial role in PeNPL synthesis, their weak surface binding affinity leads to dynamic ligand desorption, resulting in poor colloidal stability and surface defect formation, including halide vacancies and uncoordinated Pb ions [30, 31, 32]. These defects can lead to aggregation between PeNPLs or decomposition, resulting in a distribution in PeNPL thicknesses, which prevents the realization of color‐pure LEDs with a high degree of linear polarisation. This instability is further exacerbated during thin film formation, due to ligand desorption, diminishing both the stability and optical properties of PeNPLs [33, 34]. Moreover, these insulating ligands severely hinder charge‐carrier transport and injection, making them unsuitable for high‐performance LED applications [32, 35]. Therefore, to achieve efficient red‐emitting iodide‐based PeNPLs, it is essential to develop monodisperse PeNPLs while implementing advanced passivation strategies to overcome the limitations posed by native ligands.

Motivated by these challenges, we systematically investigate an ancillary ligand‐induced synthesis approach to precisely control the nucleation and growth of CsPbI_3_ PeNPLs. This involves introducing additional Lewis base ligands (i.e., ancillary ligands) to bind to the perovskite surface in addition to the oleate and oleylammonium ligands used in the synthesis of pristine PeNPLs. We herein find that the key determinants of an effective ancillary ligand are its backbone structure and Pb‐coordination strength with the perovskite surface. Ancillary ligands that optimally satisfy these two determining factors modulate the crystal growth kinetics, enabling the formation of uniform 3ML‐thick PeNPLs while simultaneously passivating surface defects. These PeNPLs not only retain homogeneous emission upon thin‐film formation but also self‐assemble into well‐ordered superlattices with significantly enhanced optical properties compared to pristine PeNPLs, owing to their well‐passivated surfaces that suppress agglomeration. By achieving fine control over the orientation of the NPLs in superlattices, we improve the degree of polarization (DOP) in edge‐up NPLs.

Conversely, face‐down oriented PeNPL superlattices enhance outcoupling in the direction normal to the substrate in LEDs, leading to high external quantum efficiencies (EQEs) reaching 13.1%, which represents the highest value reported for LEDs based on ultrathin PeNPLs (i.e., 3 ML or lower), where exciton fine structure splitting becomes sufficient for realizing polarised light emission. Taken together, our results establish ancillary ligand‐induced synthesis of uniform‐ML PeNPLs as a robust route to fully exploit the multifunctionality of these anisotropic nanocrystals for next‐generation photonics and displays.

## 2 Results

### 2.1 Controlling Ligand‐Perovskite Surface Interactions to Achieve Uniform PeNPLs

**FIGURE 1:**
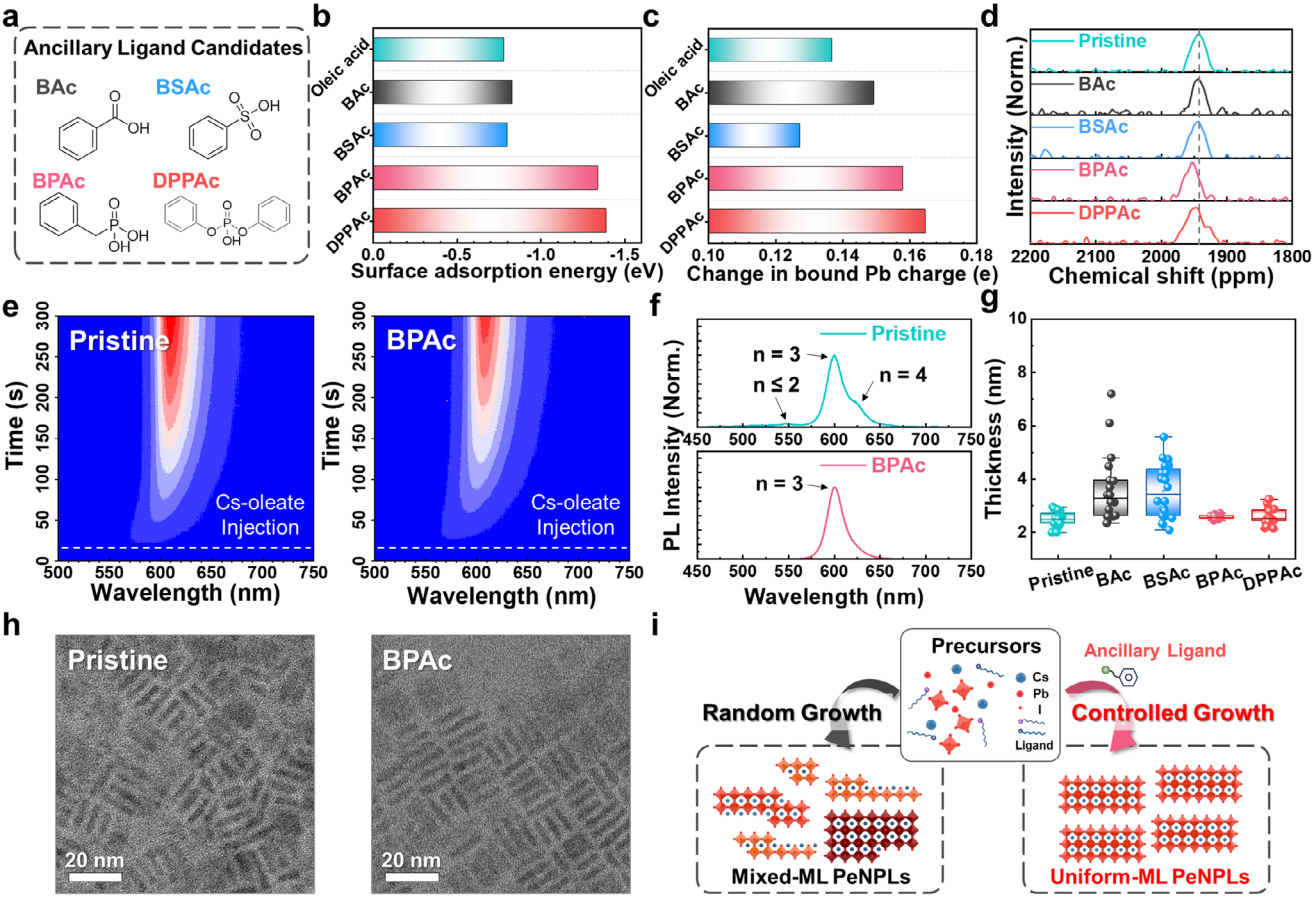
Ancillary ligand engineering to achieve well‐ordered, monodisperse CsPbI_3_ PeNPLs. (a) Candidate ancillary ligands considered in this work: benzoic acid (BAc), benzene sulfonic acid (BSAc), benzyl phosphonic acid (BPAc), diphenyl phosphate (DPPAc). (b) Calculated surface adsorption energy of ligands on the perovskite surface. (c) Calculated Bader charge on the surface Pb atom bound to the ligand molecule referenced to the charge of an unbound surface Pb atom. These charges increase upon binding, which correlates to the deshielding of Pb observed by nuclear magnetic resonance (NMR). (d) Liquid‐phase ^207^ Pb NMR spectra of PbI_2_ precursor solutions without and with ancillary ligands. (e) In situ photoluminescence spectra recorded during synthesis under ambient air conditions, excited with a 405 nm CW laser (37.3 mW cm^−2^). (f) Photoluminescence spectra of pristine‐PeNPL and BPAc‐PeNPL colloidal solutions. (g) Thickness distribution histogram of CsPbI_3_ PeNPLs determined from TEM measurements from Figure 1h and Figure S8. The target thickness during synthesis was 3 monolayers. (h) High‐resolution transmission electron microscopy (TEM) images of pristine‐PeNPLs and BPAc‐PeNPLs with an overall edge‐up orientation. These nanoplatelets were dispersed in a hexane solvent and drop‐cast onto a Cu TEM grid. (i) Schematic illustration of conventional synthesis and ancillary ligand‐induced synthesis for PeNPLs.

We hypothesized that the introduction of ligands strongly coordinating to the perovskite precursor monomers in colloidal solution can retard PeNPL formation, thus enabling regulation of crystallization and greater control over the final thickness of the PeNPLs. Within this hypothesis, the functional group of the ligand would play a decisive role, as would the organic backbone of the ligands, which can influence the growth kinetics through steric hindrance [36]. Guided by this hypothesis, we compared ligands with a carboxylic acid, sulfonic acid and phosphonic acid functional group (keeping the organic backbone to a benzyl group), i.e., benzoic acid (BAc), benzene sulfonic acid (BSAc) and benzyl phosphonic acid (BPAc). We selected these functional groups for comparison, since they have oxo‐groups available for coordination with Pb^2+^ as Lewis bases [37, 38, 39]. We also compared ligands with the same phosphoryl functional group, but changed the organic backbone, i.e., BPAc vs. diphenyl phosphate (DPPAc). The ancillary ligand candidates are illustrated in Figure 1a.

Understanding the interactions between Pb precursors and coordinating ligands at the atomic scale is critical for elucidating how crystallization can be regulated. Prior to experimental investigation, density functional theory (DFT) calculations were performed to provide atomistic insights into ligand‐surface interactions to clarify the role of specific ligands (Computational details in **Methods**).

The calculations show that DPPAc and BPAc have the highest surface adsorption energies (Figure 1b) on the (001)‐terminated surface of the perovskite. Surface adsorption energy is defined as the energy reduction upon ligand adsorption to the perovskite surface after structural optimization of the complex. The strength of adsorption or binding can be related to the degree of PbI_6_ octahedral distortion simulated at the surface, as well as the length of the bond formed between the surface and the ligand molecules (Figure S1). DPPAc and BPAc were found to induce the highest degree of structural distortion, and both have the shortest Pb─O bond length of 2.46 Å, compared to 2.63 Å for oleic acid, the weakest‐bound ligand. The slightly stronger binding of BAc compared with oleic acid can be attributed to its more compact backbone, which reduces steric hindrance and allows closer access to surface Pb sites. However, because BAc still coordinates through a carboxylate group, its interaction with the perovskite surface remains weaker than that of BPAc and DPPAc, whose phosphoryl functional groups exhibit substantially stronger coordination. The calculated projected density of states (PDOS) for Pb and O atoms involved in bonding between BPAc and the perovskite surface shows significant Pb─O hybridization in the occupied (valence) region, indicating strong covalency between the ligand and perovskite surface (Figure S2). This is consistent with O from the phosphoryl functional group in BPAc acting as a Lewis base and coordinating with the empty Pb 6p states, with electron density shared between them. Bader charge analysis (Figure 1c) assigns slightly more electron density to O than to Pb in the Pb─O bond because of the higher electronegativity of the O atom. Comparing all ligands, the DPPAc and BPAc ligands exhibited the largest polarization of the Pb‐ligand bond (i.e., largest change in bound Pb charge, Figure 1c), which is consistent with the phosphoryl group being the functional group that binds the strongest to the perovskite surface.

To further substantiate differences in ligand‐precursor interactions, we performed liquid‐phase ^207^ Pb nuclear magnetic resonance (NMR) measurements of the PbI_2_ precursor solutions with and without the ancillary ligand added. The ^207^ Pb NMR chemical shift is highly sensitive to the coordination environment of Pb^2+^ [40]. As shown in Figure 1d, the precursor solutions containing BPAc and DPPAc exhibit a clear chemical shift from 1943.4 ppm (pristine) to 1953.2 ppm and 1949.7 ppm, indicating strong coordination of BPAc and DPPAc to Pb^2+^. Repeat measurements consistently gave the same ^207^ Pb peak positions for each condition, as well as the same trends in chemical shifts. That the BPAc and DPPAc ligands result in the largest change in chemical shift substantiates the simulated surface adsorption energies (Figure 1b) showing these ligands to have the strongest binding to the perovskite surface via the P═O groups.

We synthesized colloidal CsPbI_3_ PeNPLs using the LARP method (detailed in **Methods**). Five types of samples were prepared for comparison: pristine‐PeNPLs synthesized without ancillary ligands (pristine‐PeNPLs), and PeNPLs with ancillary ligands incorporated (BAc‐PeNPLs, BSAc‐PeNPLs, BPAc‐PeNPLs and DPPAc‐PeNPLs), prepared by adding the ligands to the PbI_2_ precursor solution (2.9 mM concentration). To elucidate the mechanism by which ancillary ligands regulate PeNPL growth, we conducted in situ photoluminescence (PL) measurements during synthesis. A 405 nm wavelength continuous‐wave (CW) laser was used as the excitation source, and the PL spectra were recorded at 1 s time intervals following the injection of the Cs‐oleate precursor into the PbI_2_‐ligand solution. As shown in Figure 1e and Figures S3 and S4, pristine‐PeNPLs exhibited a rapid onset of PL signal followed by a gradual redshift in emission wavelength, indicating the formation of PeNPLs with a mixture of different thicknesses, arising through uncontrolled nucleation and growth. BAc‐ and BSAc‐PeNPLs developed PL emission faster, reaching saturation within 250 s before declining, indicating that they did not retard crystal growth and instead destabilized the reaction dynamics. In contrast, BPAc‐ and DPPAc‐PeNPLs displayed a delayed PL onset and a slower spectral shift, suggesting suppressed nucleation and more regulated crystal growth dynamics. Although BAc possesses a similarly compact molecular backbone, its carboxylate functional group has weaker coordination with the Pb^2+^ species, and is therefore less effective at regulating precursor availability compared to the phosphonic acid ligands. As a result, BAc does not sufficiently suppress nucleation or uniformly retard PeNPL growth kinetics, resulting in poorer thickness control compared with BPAc. This trend is quantified in Figure S5, which plots the evolution of the PL peak wavelength as a function of reaction time. Pristine‐, BAc‐ and BSAc‐PeNPLs showed a fast redshift from ∼580 to 609 nm wavelength, while BPAc‐ and DPPAc‐PeNPLs gradually evolved from 570 to 608 nm wavelength, indicating more controlled lateral and vertical growth. Additionally, the PL intensity profiles during the early reaction stages (Figure S6) reveal that BPAc‐ and DPPAc‐PeNPLs exhibit a slower rise in PL intensity compared to other samples. These observations suggest that BPAc and DPPAc ancillary ligands slow down nucleation and the subsequent crystal growth kinetics. Such observations are consistent with these ligands binding more strongly with Pb^2+^, since they could delay the availability of Pb^2+^ for perovskite formation.

Figure 1f and Figure S7 show the ultraviolet‐visible absorption and PL spectra of the PeNPL dispersions. Given that the out‐of‐plane dimensions of the PeNPLs are smaller than the Bohr diameter of CsPbI_3_ (∼12 nm [41]), the optical properties of these nanocrystals are strongly influenced by quantum confinement effects. The absorption spectra of all PeNPL samples exhibit a pronounced excitonic peak, directly confirming strong quantum confinement. Pristine‐PeNPLs exhibit multiple PL peaks centered at 550, 600, and 627 nm wavelength (Figure 1f), corresponding to nanoplatelets with *n* = 2, 3, and 4 [14], respectively. These multiple emission peaks indicate poor thickness uniformity, which is a known challenge in PeNPL systems, given that these PeNPLs are achieved under kinetically controlled growth conditions, rather than thermodynamic growth conditions. Multi‐emission peaks were also found in the BAc‐, BSAc‐ and DPPAc‐PeNPLs, even peaks centered at 680 nm wavelength, which corresponds to nanoplatelets with *n* ≥ 5 MLs. By contrast, BPAc‐PeNPLs exhibit a single, sharp emission peak at 600 nm wavelength (FWHM ∼ 21 nm), consistent with the formation of uniform 3ML‐thick nanoplatelets. The absorption spectra further corroborate this observation, with the absence of higher or lower energy transitions confirming the suppression of undesired thickness variants.

To further validate the morphological and structural uniformity, high‐resolution transmission electron microscopy (HR‐TEM) was performed on edge‐up oriented PeNPLs (Figure 1g,h; Figure S8) and face‐down oriented PeNPLs (Figures S9 and S10). Detailed HR‐TEM analysis revealed the thicknesses of 2.50 ± 0.29 nm for pristine‐PeNPLs, 3.63 ± 0.12 nm for BAc‐PeNPLs, 3.54 ± 0.98 nm for BSAc‐PeNPLs, 2.57 ± 0.06 nm for BPAc‐PeNPLs, and 2.64 ± 0.39 nm for DPPAc‐PeNPLs. Notably, among all samples, BPAc‐PeNPLs is the only one that shows a narrow thickness distribution near the target 3 ML thickness of CsPbI_3_ PeNPLs (≈2.6 nm) [18, 25]. PeNPLs with this ancillary ligand also had aspect ratios that were closest to unity (Figure S10). The significantly narrower thickness distribution in BPAc‐PeNPLs directly reflects enhanced monodispersity, consistent with the optical results. However, it is curious that BPAc led to improved monodispersity than DPPAc, despite both having P═O groups that bind to Pb^2+^, and DPPAc and BPAc having similar surface adsorption energies. To understand this apparent discrepancy and rationalize the effects of steric hindrance during NPL growth, we conducted ^1^H‐NMR to characterize the surface chemistry of BPAc‐ and DPPAc‐PeNPLs (Figures S11 and S15c). Ferrocene (0.5 mg mL^−1^) was introduced into each PeNPL solution in DMSO‐d_6_ as an internal reference for quantitative analysis. In the ^1^H‐NMR spectra, the resonance at 4.17 ppm is assigned to ferrocene, and the signal at 5.33 ppm corresponds to the vinylic protons (‐HC═CH‐) of the native ligands [42, 43]. Quantitative integration of these peaks relative to ferrocene shows that DPPAc‐PeNPLs have significantly decreased amounts of native oleate/oleylammonium ligands than pristine‐ and BPAc‐PeNPLs, despite comparable target ligand density. This difference can be rationalized from the more sterically bulky backbone of DPPAc, which would hinder the attachment of native ligands to the perovskite surface during growth and purification, leading to reduced colloidal stability and increased polydispersity. Overall, the results in Figure 1 underscore two notable conclusions. First, the incorporation of functional groups, such as P═O, which exhibit strong binding to Pb, is crucial for regulating crystal growth, thereby enabling the synthesis of uniform‐ML PeNPLs. Second, even when crystal growth is effectively controlled, the steric bulk of the ancillary ligands can modulate the surface chemistry and, in turn, alter the uniformity in thickness of the resulting PeNPLs.

Based on the understanding of the nucleation and growth kinetics of ancillary ligand incorporation, to investigate the impact of the BPAc ligands on the optical properties of PeNPLs, we performed photoluminescence quantum yield (PLQY) and time‐resolved photoluminescence (TRPL) measurements for both pristine‐PeNPL and BPAc‐PeNPL colloidal solutions. As shown in Figure S12, BPAc‐PeNPLs exhibit a maximum PLQY of 65.7% and a median PLQY of 62.7 ± 2.8% across 9 independently prepared samples, measured at 400 nm excitation wavelength. In contrast, pristine‐PeNPLs show significantly lower values, with a maximum PLQY of 36.1% and a median value of 34.3 ± 2.2% across 9 independently‐prepared samples. The expanded PLQY comparison including BAc‐, BSAc‐, and DPPAc‐PeNPLs shows a trend broadly consistent with the ligand binding energies discussed above, with BPAc‐ and DPPAc‐PeNPLs exhibiting higher colloidal PLQYs than BAc‐ and BSAc‐PeNPLs. The increased PLQY of BPAc‐PeNPLs suggests effective passivation of surface defects, reducing nonradiative recombination. Time‐resolved PL decay measurements further support the strongly passivating nature of BPAc ligands. Across all excitation fluences, the average carrier lifetime in BPAc‐PeNPLs exceeds that in pristine‐PeNPLs, consistent with reduced non‐radiative recombination (Figure S13 and Table S1). These findings highlight the critical role of BPAc in suppressing defect‐induced nonradiative losses, resulting in improved optical properties of PeNPLs. This can also be supported by the decay curves extracted from transient absorption (TA) spectroscopy measurements of pristine‐ and BPAc‐PeNPL colloidal solutions shown in Figure S14, where the pristine‐PeNPLs exhibited faster decay than BPAc‐PeNPLs. Further experimental evidence supporting BPAc coordination to the PeNPL surface is obtained from ^1^H NMR, ^31^P NMR, X‐ray Photoelectron Spectroscopy, and Fourier transform infrared spectroscopy measurements (detailed in Notes S1 and S2). These experimental observations are consistent with the DFT‐predicted strong binding between the BPAc ligand and the perovskite surface, reinforcing the dual role of BPAc ancillary ligand as both a kinetic modulator and an effective surface passivator. This dual functionality leads to the formation of highly uniform 3ML CsPbI_3_ PeNPLs with enhanced optical properties, including suppressed nonradiative recombination and prolonged charge‐carrier lifetimes. Such improvements in structural and photophysical properties are critical prerequisites for the formation of well‐ordered, and highly‐emissive PeNPL assemblies in thin films, which are further investigated using grazing‐incidence wide‐angle X‐ray scattering (GIWAXS) and X‐ray diffraction (XRD) in the following section.

### 2.2 Perovskite Nanoplatelet Uniformity in Films

**FIGURE 2:**
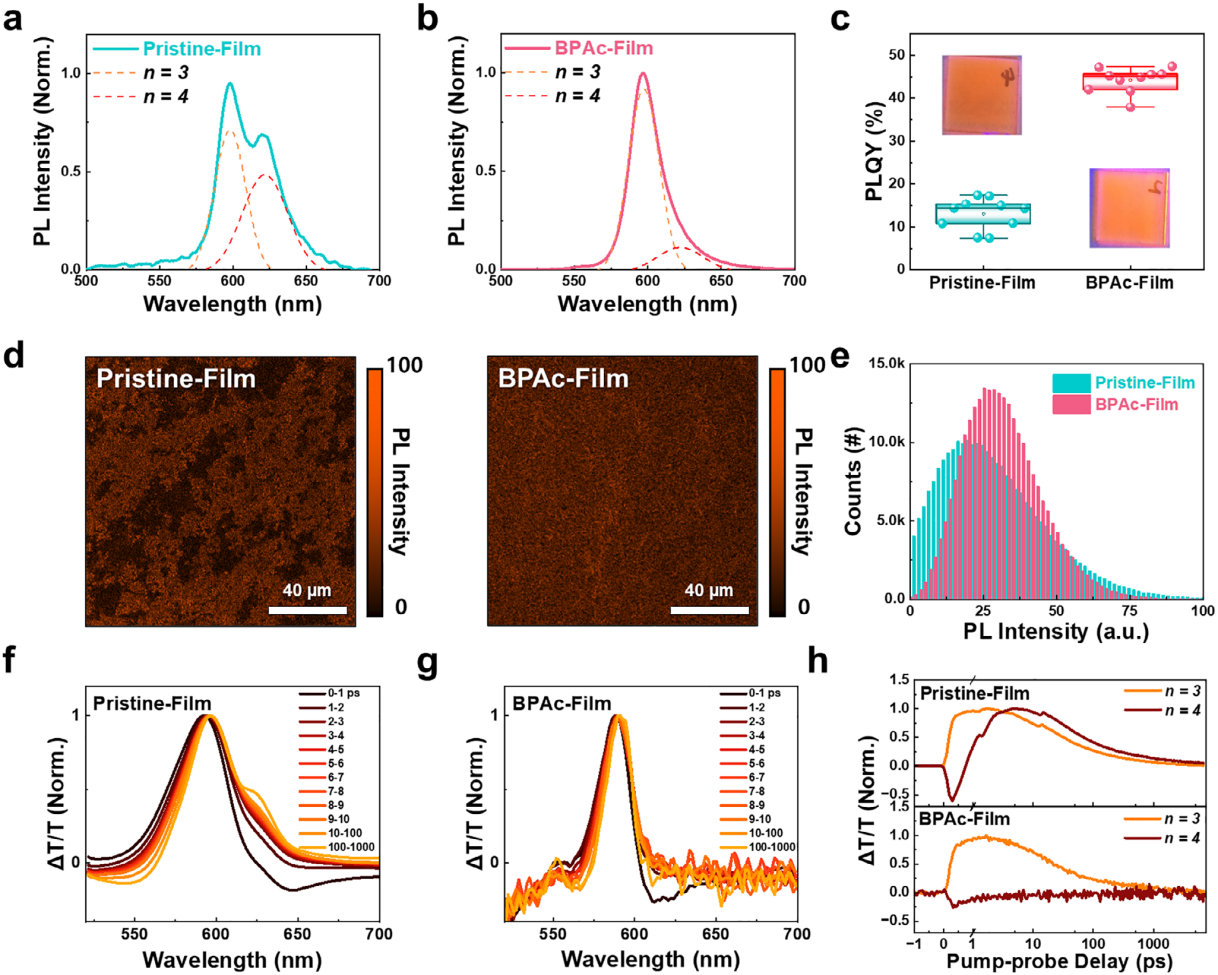
Optical characterization of pristine‐ and BPA‐treated CsPbI_3_ PeNPL films. PL spectra of (a) Pristine‐PeNPL and (b) BPAc‐PeNPL thin films showing contributions from both 3ML and 4ML components. (c) PLQY distributions and (d) confocal PL images of each PeNPL thin film (scan area: 120 × 120 µm^2^). (e) PL intensity histograms extracted from the confocal PL mapping images. Normalized TA spectra from (f) pristine‐ and (g) BPAc‐PeNPL films over the first 1000 ps after photoexcitation, excited with a 400 nm wavelength pulsed laser (17.28 µJ cm^−2^ fluence). (h) Charge‐carrier kinetics of the 3 ML (probed at 580 – 600 nm wavelength) and 4 ML (probed at 610–630 nm wavelength) PeNPLs present in the films. Positive signals represent the ground states bleach (GSB) signals. Δ*T*/*T* values normalized to the peak GSB for each trace.

Depositing PeNPLs from colloidal solution to form films can lead to ligand detachment and PeNPL agglomeration. To investigate the film‐formation behavior of PeNPLs, each PeNPL thin film was fabricated by spin coating from colloidal solution. Indeed, pristine‐PeNPL thin films (Figure 2a) show broadened emission with two distinct peaks at 600 and 627 nm emission wavelength, corresponding to 3ML and 4ML PeNPLs, respectively. This spectral broadening is more pronounced than that observed in colloidal solution (Figure 1f), indicative of NPL agglomeration [44]. In contrast, BPAc‐PeNPL thin films (Figure 2b) exhibit a dominant, narrow PL peak centered at 600 nm. Spectral deconvolution reveals that the 600 nm component accounts for 53.6% of the total PL in pristine films, increasing to 84.7% in BPAc‐PeNPL films, with the 627 nm wavelength component of the total PL emission reduced from 46.4% to 15.3%. The more dominant PL emission at 600 nm (3ML) can be attributed to the strong coordination between BPAc ligands and the perovskite surface, which prevents aggregation and preserves the size distribution even during thin film formation. PLQY measurements of the PeNPL films further supports these observations. As shown in Figure 2c, pristine‐PeNPL thin films exhibit a PLQY of 17.4% (median: 13.0% across 9 samples). In contrast, BPAc‐PeNPL thin films retain a high PLQY of 47.4% (median: 44.0% across 9 samples), comparable with the PLQY in colloidal solution. This result is consistent with the passivation effects observed in the solution‐phase measurements and confirms that BPAc ligand binding effectively suppresses nonradiative recombination pathways even in thin film form. The PL spectra and PLQY values of BAc‐, BSAc‐, and DPPAc‐PeNPL thin films shown in Figures S19 and S20 are broadly consistent with the optical properties observed in colloidal solution. In particular, although DPPAc‐PeNPLs show relatively high PLQY in solution, their thin‐film PLQY decreases markedly after film formation. As discussed in Figure S11 and Note S1, ^1^H NMR analysis revealed that BPAc and DPPAc are present in comparable amounts on the PeNPL surface, whereas the native oleate/oleylammonium ligand density is lower for DPPAc‐PeNPLs. This reduced native ligand coverage likely makes the DPPAc‐PeNPL surface more vulnerable to damage and aggregation during film formation, thereby leading to increased nonradiative recombination and lower PLQY in the thin‐film state. The uniformity of the PeNPL film was evaluated through PL mapping using a confocal microscope (Figure 2d), with the corresponding intensity histograms shown in Figure 2e. Pristine‐PeNPL thin films display heterogeneous emission intensity across the scanned area, and have a broad distribution in PL intensities (Figure 2e). In contrast, BPAc‐PeNPL thin films exhibit significantly enhanced PL uniformity, with narrower and more symmetric intensity profiles. This homogeneity is attributed to the enhanced PeNPL monodispersity and strong surface passivation provided by the BPAc ancillary ligand.

The improved monodispersity also influences the charge‐carrier dynamics in PeNPL thin films. Although the presence of ligands can suppress charge transfer between different PeNPLs [45], energy transfer between PeNPLs with different thicknesses (and thus different optical gaps) can still occur and degrade device performance [46, 47]. This energy transfer between NPLs of different thicknesses is much less pronounced in colloidal solution, where the NPLs are relatively far apart and separated by solvent molecules. Figure S14a shows the normalized TA spectra of the pristine PeNPLs in solution. Both characteristic ground state bleach (GSB) peaks of the 3ML (580–600 nm) and 4ML (610–630 nm) populations are present. The relative intensity ratio between these two peaks does not change over time, and the GSB decay curves for the two thicknesses show no overlap at early times (< 1 ns), indicating negligible energy transfer between NPLs in solution. In contrast, Figure 2f presents the normalized TA spectra of the pristine PeNPL thin film, where the relative intensity of the 4ML bleach peak initially increases over time. Moreover, Figure 2h shows clear overlap between the GSB decay curves of the 3ML and 4ML PeNPLs within each film. Together, these observations suggest substantial energy transfer in the pristine samples from the higher optical‐gap 3ML PeNPLs to the lower optical‐gap 4ML PeNPLs in the film. This additional energy‐transfer pathway introduces an extra nonradiative decay channel for photoexcited or electrically‐injected charge‐carriers, thereby reducing overall device performance and compromising emission purity. By comparison, the BPAc‐PeNPLs exhibit a more uniform thickness distribution. The substantially suppressed population of 4ML PeNPLs results in the 4ML GSB peak at 610–630 nm being negligible above noise in both the solution and film samples, as shown in Figure S14b and Figure 2g. This suppression of inter‐thickness energy transfer enables more efficient radiative recombination in BPAc‐PeNPL thin films.

### 2.3 Formation and Characterization of PeNPL Superlattices

**FIGURE 3:**
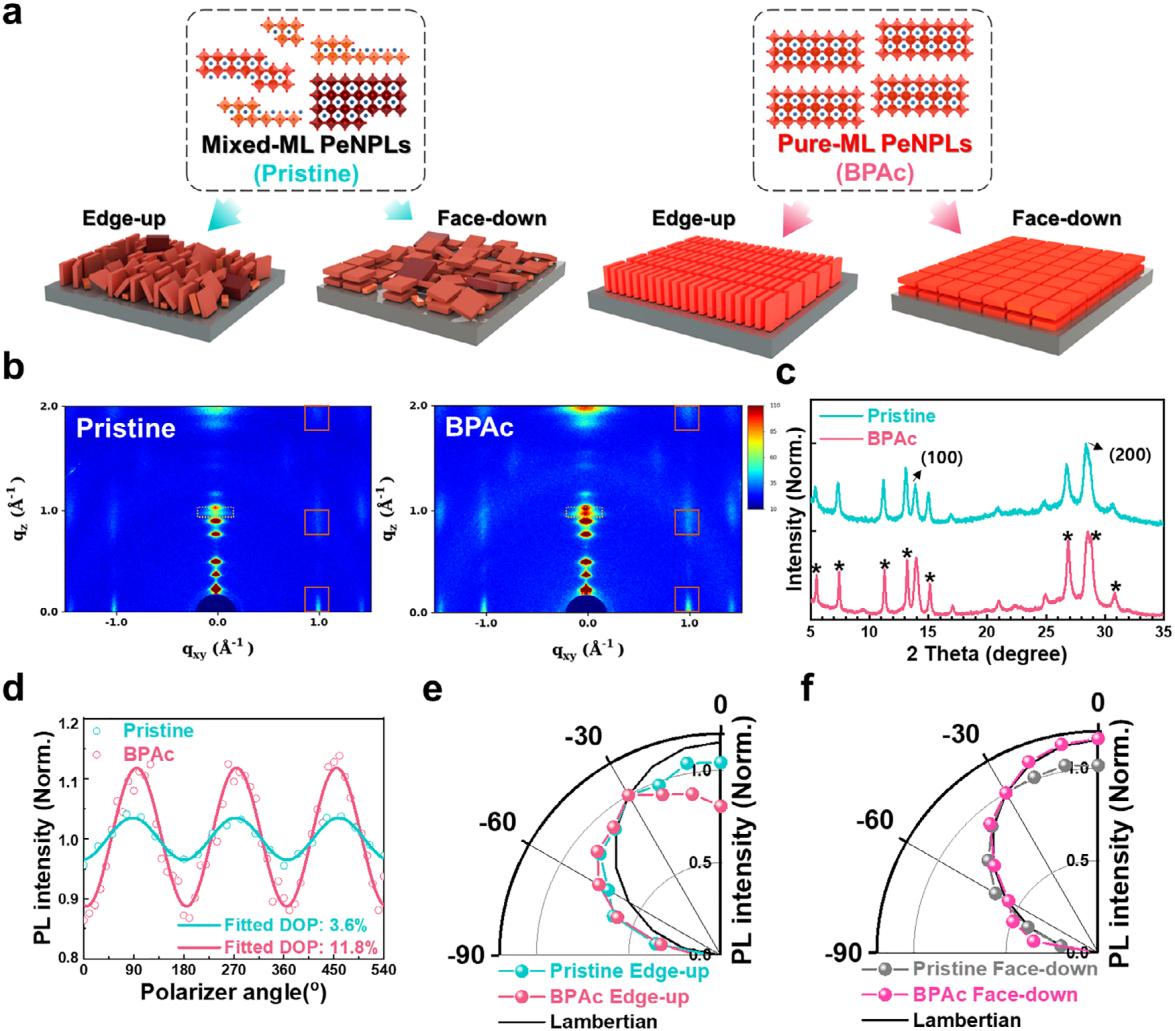
Structural characterization and linearly polarized light emission from superlattice films. (a) Schematic illustration of superlattice formation from mixed‐ML and uniform‐ML PeNPLs. (b) Grazing‐incidence wide‐angle X‐ray scattering (GIWAXS) patterns of pristine‐ and BPAc‐PeNPLs spin coated from a solution with octane with an overall face‐down orientation. (c) X‐ray diffraction (XRD) patterns of pristine‐ and BPAc‐PeNPL thin films spin coated from a solution with hexane with an overall edge‐up orientation. The peaks marked with stars correspond to the superlattice peaks, while the Bragg peaks are labelled with Miller indices. (d) Polarization angle dependence of normalized PL intensity of pristine‐ and BPAc‐PeNPL thin films, fitted with a sinusoidal function. Lambertian profile and angular distribution of the normalized PL intensity from (e) edge‐up and (f) face‐down pristine‐ and BPAc‐PeNPL thin films.

To further evaluate the structural properties of the synthesized PeNPLs, we investigated their formation into superlattices. The orientation of the PeNPLs can be tuned by controlling the evaporation rate of the alkane solvent used to disperse the PeNPLs [18, 25, 48]. A schematic illustration of the self‐assembly of PeNPLs is shown in Figure 3a, where BPAc‐PeNPLs, due to their narrow size and thickness distribution, form highly ordered superlattices. In contrast, pristine‐PeNPLs, with broader size and shape variations, exhibit disordered and randomly stacked superlattices. To characterize the structural orientation and periodicity of the PeNPL superlattices, GIWAXS measurements were conducted (Figure 3b; Figures S21 and S22). Each PeNPL thin film was fabricated by spin coating colloidal PeNPL dispersions in octane and hexane. Both sets of films contain regions of face‐down PeNPL superlattices, as well as regions of edge‐up PeNPL superlattices (Figure S25). Spin coating from the more slowly‐evaporating octane favors the face‐down orientation, while spin coating from the faster‐evaporating hexane favors the edge‐up orientation. This assembly process therefore adjusts the fraction of local regions of face‐down or edge‐up PeNPL superlattices across the entire film (Figure S25). BPAc‐PeNPL thin films exhibited a series of well‐defined out‐of‐plane diffraction peaks corresponding to the (100), (101), and (102) planes of the perovskite structure, which were markedly sharper and more intense than those of the pristine‐PeNPL thin films, consistent with the formation of more ordered superlattices (Figure 3b). GIWAXS measurements of the PeNPL films spin coated from solution with octane as the solvent show a vertical spread of the diffraction peaks along the *q*_z_ direction (Figure 3b; indicated in boxes), which is consistent with coherent layer‐by‐layer stacking in the BPAc‐PeNPL films. One‐dimensional scattering profiles extracted from the integration of diffraction rings (Figures S23 and S24) show low‐angle peaks (*q* < 1 Å^−1^), which can be attributed to periodic electron density variations between the dense inorganic slabs and the surrounding organic ligands [18, 21]. These features were especially pronounced in edge‐up oriented BPAc‐PeNPL thin films, supporting the formation of superlattices with consistent interlayer spacing. To quantify the degree of orientational alignment, we further analyzed the azimuthal dependence of scattering intensity (Figures S25 and S26). In the BPAc‐PeNPL thin films, face‐down orientations exhibited stronger intensity in the low azimuthal angle range (0°–10°), while edge‐up orientations showed enhanced scattering at higher angles (80°–90°). These patterns were significantly sharper than those of pristine samples, indicating a higher degree of orientation in both face‐down and edge‐up PeNPL thin films due to the improved monodispersity of BPAc‐PeNPLs that allowed more regular stacking. Finally, 1D *θ*−2*θ* XRD patterns were recorded to confirm the periodic stacking behavior (Figure 3c; Figures S27 and S28). Both PeNPL thin films exhibited multiple satellite peaks in addition to the Bragg peaks, in agreement with prior reports on PeNPL superlattices [48, 49]. Notably, BPAc‐PeNPL thin films showed narrower FWHM values for these satellite peaks, indicating superior long‐range structural coherence and crystallinity. Collectively, these results highlight the critical role of the BPAc ancillary ligand in enabling the formation of structurally uniform PeNPL assemblies that form local superlattices, where the fraction of face‐down or edge‐up superlattice regions is adjustable by the evaporation rate of the alkane solvent the colloidal nanoplatelets are dispersed in. As demonstrated in the preceding analyses, the well‐oriented PeNPL superlattices provide a versatile platform for a variety of applications. Edge‐up oriented PeNPL thin films, characterized by their high degree of in‐plane dipole alignment, are advantageous for achieving strong linear polarization in light emission. In contrast, face‐down oriented PeNPL thin films, with their efficient out‐coupling, are suitable for LED devices, where maximizing light extraction is essential [50, 51, 52, 53]. These orientation‐dependent properties will be further explored in the following sections, demonstrating the broad applicability of PeNPL superlattices in both polarization‐sensitive photonic applications and efficient light‐emitting diodes (LEDs).

### 2.4 Linearly Polarized Light Emission and Improved Out‐Coupling

Ultrathin PeNPL light emitters inherently exhibit linearly polarized light emission at the single particle level through exciton fine structure splitting. But maintaining a high degree of polarization at the device level requires fine control over the orientation and alignment of the PeNPLs [54, 55]. Previously, we established that a high degree of polarization (DOP) in emission from the normal direction to the substrate requires edge‐up PeNPLs [18, 25, 53]. We therefore focused on PeNPL superlattice films with an overall edge‐up orientation, and measured the DOP, as detailed in Figure S29. As shown in Figure 3d, Pristine‐PeNPL films exhibited a relatively low DOP, with a maximum of 3.6% and a median of 3.4% across 5 independently prepared samples. In contrast, BPAc‐PeNPL thin films displayed a significantly enhanced polarization response, with a maximum DOP of 11.8% and a median of 11.5% across 5 independently prepared samples. The corresponding DOP histogram is provided in Figures S30 and S31, with individual data points summarized. Angle‐resolved PL measurements of PeNPL superlattice films with an overall edge‐up orientation further supports this trend (Figure 3e). In these measurements, 0° corresponds to light collection normal to the thin film surface, while 90° represents collection parallel to the thin film plane. The PL intensity variation with respect to collection angle was significantly more pronounced in BPAc‐PeNPL thin films than in their pristine‐PeNPL thin films. This behavior indicates enhanced transition dipole alignment, as evidenced by the GIWAXS results, which show that an increase in the percentage of PeNPLs oriented edge‐up (Figure S25) from pristine (50%) to BPAc‐PeNPLs (54.5%).

The advantage of being able to tune the orientation of the PeNPL film is that we can also tune their orientation to face‐down in order to maximize outcoupling in the normal direction by maximizing the fraction of horizontal transition dipole moments [53, 56, 57]. The angular distribution profiles of PL from the superlattices with an overall face‐down orientation based on pristine‐PeNPLs and BPAc‐PeNPLs are shown in Figure 3f. These measurements also show that the BPAc‐PeNPLs exhibit a more Lambertian profile, in contrast to the more directional emission observed in the pristine‐PeNPL films. The improved angular emission profile with BPAc treatment is consistent with our previous optical simulations of CsPbI_3_ PeNPLs indicating that a higher fraction of horizontal transition dipole moments leads to more Lambertian‐like emission [18], in line with the more pronounced face‐down alignment suggested by our GIWAXS results (Figure S25c). These results also suggest that BPAc‐PeNPLs would exhibit higher outcoupling, which is particularly advantageous for LED applications [50, 51, 58].

### 2.5 Realizing Efficient Strongly‐Confined PeNPL LEDs in the Ultrathin Regime

**FIGURE 4:**
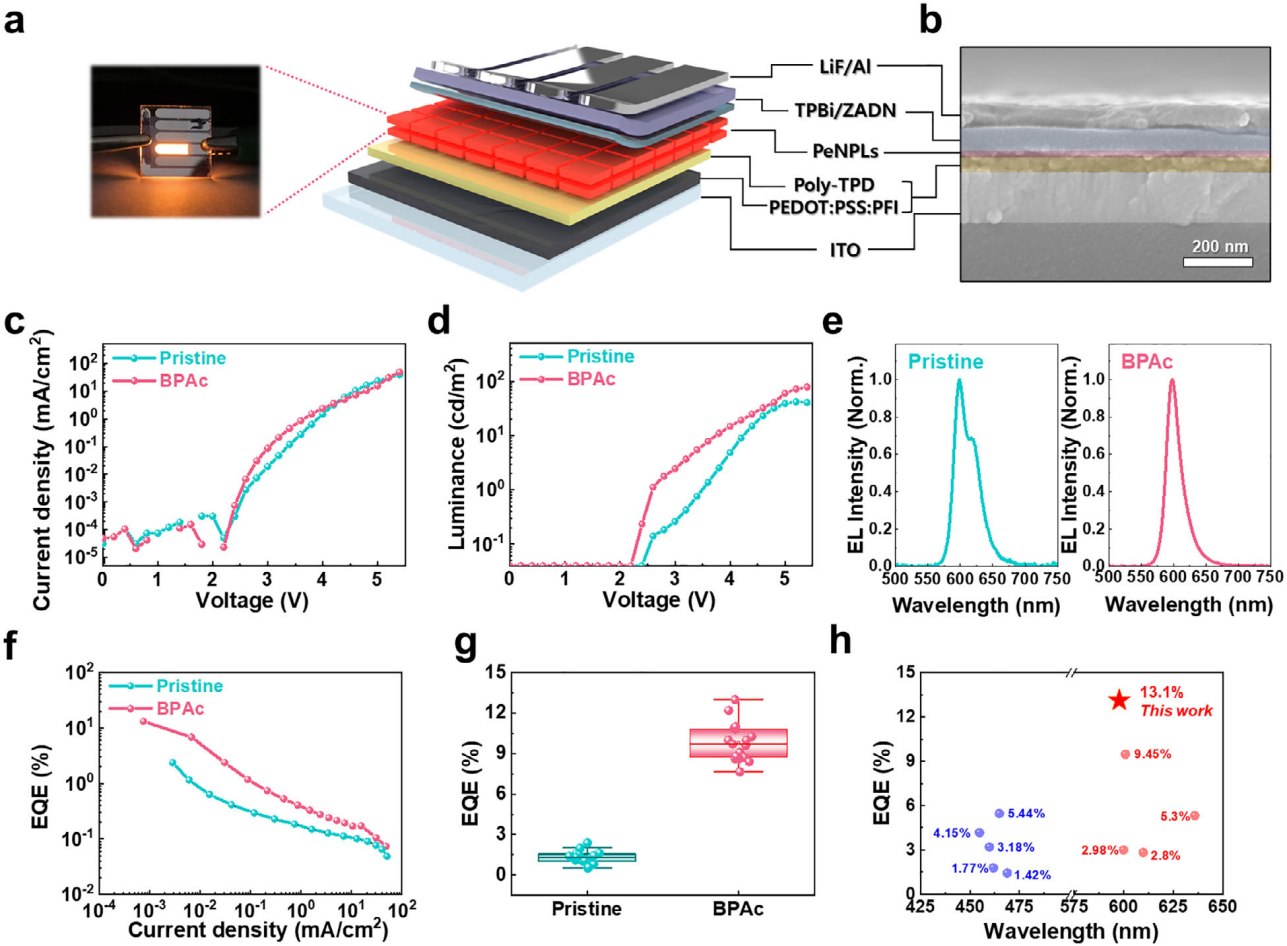
Device performance of pristine‐ and BPA‐treated CsPbI_3_ PeNPLs with an overall face‐down orientation. (a) Device architecture and (b) cross sectional scanning electron microscopy (SEM) images of LEDs. Comparison of the (c) current density–voltage (*J–V*) curve, (d) luminance–voltage (*L–V*) curve, (e) electroluminescence (EL) spectra, and (f) external quantum efficiency (EQE)–current density (*EQE–J*) curves of champion devices. (g) EQE_max_ histogram from 17 individual PeNPL devices of each type. (h) Comparison of the EQE_max_ of strongly‐confined CsPbX_3_ (X = I, Br) PeNPL LEDs in the ultrathin regime (*n* ≤ 3) with previous literature [18, 28, 29, 59, 60, 61, 62, 63, 64].

To evaluate the optoelectronic performance of the synthesized PeNPLs, we fabricated LEDs with the device architecture illustrated in Figure 4a. The device stack consists of an indium tin oxide (ITO) transparent electrode, followed by a bilayer hole transport layer composed of PEDOT:PSS:PFI and poly(N,N′‐bis‐4‐butylphenyl‐N,N′‐bisphenyl)benzidine (Poly‐TPD). The emissive layer comprises of either pristine‐ or BPAc‐PeNPLs. To maximize outcoupling, we spin‐coated the PeNPLs from a colloidal solution in octane to achieve an overall face‐down orientation. A bilayer electron transport structure comprised of 2,2′,2′′‐(1,3,5‐benzinetriyl)‐tris(1‐phenyl‐1‐H‐benzimidazole) (TPBi) and 2‐[4‐(9,10‐di‐naphthalen‐2‐yl‐anthracen‐2‐yl)‐phenyl]‐1‐phenyl‐1H‐benzoimidazole (ZADN) was deposited on top of the PeNPL layer, followed by LiF/Al as the top electrode. A cross‐sectional scanning electron microscopy (SEM) image of the complete device structure is shown in Figure 4b, confirming uniform layer stacking and a smooth interface with the PeNPL emissive layer. The orientation of PeNPLs within the thin film significantly influences device performance due to orientation‐dependent optical outcoupling (detailed discussion in Note S3). To quantify this effect, optical simulations were performed using the face‐down/edge‐up orientation ratios of the PeNPL films determined from GIWAXS measurements (Figure S25). Compared with the isotropic emitter reference case, the theoretical maximum efficiency increased from 32.68% to 35.31% for pristine‐PeNPL films (where 50.3% of the PeNPLs were face ‐ down) and further to 36.72% for BPAc‐PeNPL films that had 60% of the PeNPLs face‐down. These simulations confirm that an increased face‐down alignment provides an additional optical advantage through enhanced outcoupling in the normal direction. Thus, BPAc‐PeNPL films that had an overall face‐down orientation (spin coated from slowly‐evaporating octane solvent) were selected for LEDs due to their superior outcoupling in the normal direction, and these data are shown in Figure 4 and Table 1.

**TABLE 1:** Device performance of champion LEDs based on pristine‐ and BPAc‐PeNPLs with an overall face‐down orientation.

Sample configuration	EQE_max_ [%] @ bias	L_max_ [cd/m^2^] @ bias	LE_max_ [cd/A] @ bias	Turn‐on Voltage [V] @ 0.1 cd/m^2^
Pristine‐PeNPLs	2.4@2.6V	42@4.8V	5.1@2.6V	2.6
BPAc‐PeNPLs	13.1@2.4V	78@5.4V	31.2@2.4V	2.4

From the current density–voltage (*J–V*) and luminance–voltage (*L–V*) measurements (Figure 4c,d), devices based on BPAc‐PeNPLs exhibit improved charge‐carrier injection and a lower turn‐on voltage compared to pristine‐PeNPL devices. This improvement is attributed to the partial replacement of long‐chain, insulating native ligands in pristine‐PeNPLs with shorter BPAc ligands, which enhance charge carrier mobility and facilitate stronger interparticle coupling within the emissive layer. Additionally, the well‐ordered superlattice formed by the uniform‐ML BPAc‐PeNPLs promotes efficient charge transport and a uniform and narrow emission profile, as discussed in the section on PeNPL superlattice structural characterization. Electroluminescence (EL) spectra of the pristine‐PeNPL and BPAc‐PeNPL LED devices are shown in Figure 4e and Figure S35. Pristine‐PeNPL LEDs exhibited broad EL profiles with multiple emission peaks corresponding to mixed 3ML and 4ML PeNPLs, consistent with PL measurements from these films (Figure 2a). In contrast, BPAc‐PeNPL LEDs displayed a single, narrow EL peak centered at 600 nm wavelength, attributed to uniform 3ML PeNPL emission. This spectral purity, resulting from the improved well‐controlled thickness achieved through BPAc incorporation, is further corroborated by the absence of multiple EL peaks in BPAc‐PeNPL devices, confirming the structural integrity and strong exciton confinement of the PeNPL layer. Following device optimization, the champion BPAc‐PeNPL device achieved a maximum EQE of 13.1%, significantly surpassing the 2.4% EQE of the champion pristine‐PeNPL device (Figure 4f and Table 1). This performance was further validated by statistical analysis of 17 independently‐fabricated devices (Figure 4g), demonstrating high reproducibility and a narrow distribution in EQE values. In addition, operational stability measurements show that the BPAc‐PeLEDs exhibit a substantially extended half‐lifetime (T_50_) compared to pristine‐PeLEDs, increasing from ∼5 to ∼20 min at comparable initial luminance (∼30 cd m^−2^), as shown in Figure S36a. Furthermore, under a constant current density (0.25 mA cm^−2^), the BPAc‐PeLEDs exhibit significantly prolonged operational stability, sustaining emission for up to ∼72 min, whereas the pristine‐PeLEDs degrade within ∼15 min (Figure S36b). This improvement is consistent with the enhanced surface passivation and more homogeneous superlattice structure of the BPAc‐PeNPL films, which likely suppress defect‐assisted degradation during device operation. To the best of our knowledge, this value represents the highest reported EQE for strongly confined colloidal CsPbX_3_ PeNPL‐based LEDs in the ultrathin regime (i.e., 3 ML thick or thinner, Figure 4h and Figure S37 and Table S2). However, the maximum luminance of the BPAc‐PeNPL devices remains limited, suggesting that the present ancillary ligand effectively preserves the ultrathin PeNPL structure but does not yet provide sufficient ligand replacement for efficient charge injection. Further ligand‐engineering studies are therefore needed to achieve a higher degree of ligand replacement while maintaining the structural integrity and surface passivation of the PeNPLs. As explained in the introduction, this ultrathin regime is critical for realizing polarized light emission, and it is much more challenging to achieve efficient device performance in this ultrathin regime due to the prominent role of surfaces and greater difficulty in controlling thickness uniformity. The enhanced device performance observed in BPAc‐PeNPL LEDs can be attributed to several key factors. First is the efficient outcoupling arising from the well‐aligned, face‐down PeNPL superlattices with precisely controlled shape and thickness (Figure 3f). Second, the strong binding of the ancillary BPAc ligands to the perovskite surface effectively passivates surface defects, reducing nonradiative losses. These synergistic effects contribute to the superior device performance of BPAc‐PeNPL LED devices compared to the pristine‐PeNPL LEDs. Overall, the successful integration of BPAc as an ancillary ligand not only enhances the device efficiency of PeNPL‐based LEDs but also improves their linear polarization properties. These findings demonstrate the feasibility of ligand engineering as a promising approach to advance the development of high‐efficiency, strongly confined PeNPL‐based LEDs with potential applications in both high‐performance LEDs and linearly polarized quantum light sources. Finally, we compared the device performance of LEDs based on PeNPL films with an overall edge‐up orientation (Figure S34). These edge‐up PeNPL LEDs exhibit lower efficiencies than the face‐down PeNPL devices due to reduced outcoupling in the normal direction. This performance difference highlights the critical role of orientation in optimizing light extraction and achieving high‐efficiency PeNPL‐based LEDs.

## 3 Conclusions

This work reveals how the molecular structure of the ancillary ligand introduced during synthesis governs the nucleation, growth, and assembly of CsPbI_3_ nanoplatelets through a synergistic balance between headgroup coordination and backbone steric effects. The coordination strength of the functional group to Pb^2+^ influences the barrier to nanoplatelet nucleation, while the steric profile of the organic ligand backbone dictates anisotropic growth and colloidal stability. When these two factors are optimally balanced, the growth pathway converges toward PeNPLs with greater uniformity and well‐passivated surfaces, providing a molecular rationale that links ligand chemistry to nanoscale morphology and photonic function. The resulting PeNPLs are more uniformly oriented and exhibit enhanced optical properties, suppressed nonradiative losses, and tunable optical anisotropy. By precisely controlling the orientation of the PeNPLs, we achieved a remarkable EQE of 13.1% in LED devices with an overall face‐down orientation through a combination of improved outcoupling (as confirmed through optical simulations) and defect passivation, while PeNPLs with an overall edge‐up orientation exhibit enhanced linearly polarized light emission. Overall, our findings show that achieving PeNPLs with improved uniformity and PLQY can be realized using ancillary ligands containing a strong Lewis base in the functional group, as well as an organic backbone that is not overly bulky, such that they do not displace native ligands on the PeNPL surface. These design principles can be more broadly applied across the wider PeNPL family, as well as other NPL materials systems. The improvements in performance and uniformity can allow the multifunctionality of anisotropic NPLs to be fully utilized, particularly NPLs in the more challenging ultrathin regime required for achieving polarized light emission.

## 4 Methods

### 4.1 Materials

Oleylamine (OAm, 70%), oleic acid (OA, 90%), hexane (anhydrous, 95%), octane (anhydrous, ≥99%), methyl acetate (anhydrous, 99.5%), toluene (anhydrous, 99.8%), chlorobenzene (anhydrous, 99.8%), dimethyl sulfoxide‐d_6_ (99.9 atom % D, contains 0.03% (v/v) TMS), ferrocene (for synthesis), Benzoic acid (ACS reagent grade, ≥99.5%, crystalline), Benzenesulfonic acid (98.0% (T)), Diphenyl phosphate (99%) and cesium acetate (CsAc, 99.9% trace metals basis) were purchased from Sigma Aldrich. Poly(3,4‐ethylenedioxythiophene)‐poly(styrenesulfonate) (PEDOT:PSS) was purchased from Clevios. Nafion perfluorined resin solution (PFI) was purchased from Aladdin. 2, 2′, 2′′‐(1,3,5‐benzinetriyl)‐tris(1‐phenyl‐1‐H‐benzimidazole) (TPBi) and Poly‐TPD were purchased from OSM. 2‐[4‐(9,10‐Di‐naphthalen‐2‐yl‐anthracen‐2‐yl)‐phenyl]‐1‐phenyl‐1H‐benzoimidazole (ZADN) was purchased from Ossila. Benzyl phosphonic acid (BPAc, 97%) was purchased from Thermo Scientific Chemicals. Lead (II) Iodide (PbI_2_, 99.99%, trace metals basis) was purchased from Tokyo Chemical Industry.

### 4.2 Synthesis and Purification of CsPbI_3_ PeNPLs

The synthesis of CsPbI_3_ PeNPLs is based on previous reported method and modified. For Cs‐oleate preparation, 38.4 mg CsAc, 4 mL OA (0.05 mol L^−1^) was mixed in a vial and reacted at 100°C for 1 h. Cs‐oleate was cooled down and stored at room temperature. For PbI_2_ precursor solution, 92.2 mg PbI_2_, 0.2 mL OA/OAm, 10 mL toluene (0.02 mol L^−1^) was mixed with stirring at 100°C for 1 h and stored at room temperature. For the synthesis of pristine‐PeNPLs, 4 mL of PbI_2_ solution was heated to 40°C and prepared Cs‐oleate (240 µL) was injected into PbI_2_ solution and reacted by stirring for 5 min. For the synthesis of ancillary ligand‐PeNPLs, 11.6 µmol ancillary ligand was added to 4 mL PbI_2_ solution and sonicated to dissolve ligand powder. After fully dissolving the ligand, PbI_2_ solution was heated to 40°C and prepared Cs‐oleate (240 µL) was injected into PbI_2_ solution and reacted by stirring for 5 min. For purification, synthesized PeNPLs were carried into N_2_ filled glove box. The crude PeNPL solution was centrifuged at 11 000 rpm for 2 min. The precipitate was discarded. The supernatant and methyl acetate were mixed with 1:1 ratio followed by centrifugation at 11 000 rpm for 1 min. The precipitated PeNPLs were dispersed in octane or hexane, centrifuged at 11 000 rpm for 2 min to remove residues. The supernatant CsPbI_3_ PeNPLs solution was collected for further use.

### 4.3 PeNPL LED Fabrication

ITO‐coated glass substrates were cleaned with sonication in distilled water, acetone, and isopropyl alcohol, sequentially for 20 min per each step. After drying, the ITO substrate was treated to UV‐ozone for 40 min. The PEDOT:PSS:PFI solution (2:1 volume ratio) was spin‐coated on ITO substrate at 5000 rpm for 30 s and annealed at 100°C for 5 min and 150°C for 20 min, sequentially. After that, substrates were carried into a N_2_ filled glove box. Poly‐TPD (4 mg ml^−1^ in chlorobenzene) layer was spin‐coated on PEDOT:PSS:PFI layer at 4000 rpm for 30 s and annealed at 140°C for 20 min, subsequently. After deposited HTLs, prepared PeNPLs solution was spin‐coated at 1500 rpm for 30 s. The substrate was transferred to vacuum evaporator (<10^−6^ Torr) and TPBi (20 nm), ZADN (50 nm), LiF (1 nm) and Al (100 nm) were sequentially thermally evaporated. The emissive area is defined as 0.135 cm^2^ on the LED device. Overall device structure was optimized to achieve high EQE. Although our previous device structure showed relatively higher luminance but low EQE [18], in the present work we adopted a device architecture that enables more efficient and faster hole and electron injection to maximize the peak EQE. This optimization improved the peak efficiency, but the maximum luminance remained limited, suggesting that, for the present PeNPL emissive material, achieving high peak EQE and high‐luminance operation remains a trade‐off in this device architecture. These results suggest that achieving both high luminance and high EQE will require further improvement in the electrical properties of the PeNPLs and corresponding optimization of the device architecture.

### 4.4 Characterization

UV–vis absorption spectra were measured with Shimadzu UV–2600 UV–vis spectrometer. Steady‐state PL measurements were carried out using a 405 nm CW (37.3 mW cm^−2^) laser and signal was collected with QE Pro (Ocean Optics). TEM images were obtained via TEM‐2100F (JEOL) operating at an acceleration voltage of 200 kV. ^207^ Pb and ^1^H NMR sample was prepared by dissolving PeNPLs into dimethyl sulfoxide‐d_6_. ^207^ Pb and ^1^H NMR was conducted with 600 MHz FT‐NMR (AVANCE NEO 600, Bruker). Solution and thin film state PLQY measurement was conducted with commercial PLQY setup (Quantaurus‐QY Absolute PL quantum yield spectrometer HAMAMATSU), and samples were excitation at the 400 nm with xenon lamp. Fourier transform infrared spectroscopy (FTIR) was obtained using a Vertex 80v (Bruker) with ATR mode. X‐ray photoelectron spectroscopy were performed by AXIS‐NOVA (Kratos analytical) equipped with a monochromatic Al‐Ka (1486.6 eV) photon source. XPS spectra were referenced to the C 1s peak at 284.8 eV. To prevent surface charging during measurement, the NPL films were connected to the sample holder using conductive carbon tape. Time‐resolved photoluminescence were recorded using a confocal PL microscopy. The laser employed is a Coherent Mira 900 Ti:Sapphire laser. An Inrad 5–050 ultrafast harmonic generation system is used to generate the 400 nm wavelength laser. The second harmonic is used as the excitation source for PL. The repetition rate is 7.6 MHz, and the beam used had a 2 µm radius after passing the excitation laser through a confocal PL microscopy. The detector at the collection pass is a single photon avalanche didoes from PicoQuant Micro Photon Devices (MPD) series. Transient absorption measurements are performed using HARPIA transient absorption system (Light Conversion) at room temperature. The fundamental laser (PHAROS, Light Conversion, 1030 nm, 10 kHz) was divided into two beams for the generation of pump and probe beam respectively. The 400 nm pump beam was generated by collinear optical parametric amplifier (ORPHEUS, Light Conversion). The probe beam was obtained by focusing 1030 nm laser on a sapphire crystal to generate a white light spectra from 520 to 850 nm. The pump beam overlapped with the probe beam at the sample. The polarization of pump beam was set to magic angle (54.7°) relative to probe beam. The solution samples are measured inside a 1 mm thick quartz cuvette. The thin film samples were prepared in the same way as the device film with epoxy gel encapsulation to avoid degradation in the air. Grazing‐incidence wide‐angle X‐ray scattering measurements were obtained at the Pohang Accelerator Laboratory (PAL, Korea, beamline PLS‐II 6D). A two‐dimensional charge‐coupled device detector (pixel size of 0.078 nm, MX225‐HS, Rayonix L.L.C.), placed at a distance of 240.5 mm from the samples, was used to record the scattering patterns. The incident angle was fixed at 0.117°. XRD patterns were measured using Bruker D8. Confocal PL images of the PeNPL films were obtained using a High Sensitive and Multiplex Confocal Microscope (LSM 980, Zeiss) with a 63x oil immersion objective. The samples were excited using a 561 nm. Cross‐SEM was performed using a Cold FE‐SEM (SU8220, Hitachi) instrument operated at 10 kV. The performance of PeLEDs were measured using a Keithley 2400 source meter and spectroradiometer (CS‐2000, Konica Minolta) under ambient conditions. Each PeLED devices were encapsulation with glass and epoxy before measured. For PL polarization measurement, a 405 nm laser diode was used to excite the film through the glass side. Circularly polarized light was generated by passing through the 405 nm laser light into a linear polarizer‐quarter waveplate optic (CP1L405, Thorlabs). After excitation of the film, one the collection beam pass, a 405 nm filter and a linear polarizer (WP50L‐VIS, purchased from Thorlabs) was placed before photodetector and spectrometer. During the PL polarization measurement, linear polarizer was rotated via a motorized rotation mount (PRM1Z8, purchased from Thorlabs) with a step of 5 and step interval of 2 s for spectrum collection.

### 4.5 Computational Modeling

First‐principles calculations were performed using density functional theory (DFT) within the Vienna Ab initio Simulation Package (VASP) [65, 66] and the projector augmented‐wave (PAW) method [67]. The PBE exchange–correlation functional [68] and VASP‐recommended PAW pseudopotentials were employed for all elements. A fully geometry optimized CsPbI_3_ bulk structure (space group 221) using a k‐point grid of 6 × 6 × 6 was used to construct the surface model. Ligand‐surface interactions were investigated using the widely studied (001) PbI_2_‐terminated perovskite surface. Atomic positions were relaxed for surface slabs until forces were below 0.01 eV Å^−1^ and total energy converged to 10^−4 ^eV. Grimme's DFT‐D3 correction was included to account for van der Waals interactions [69]. A 500 eV plane‐wave cutoff and a Γ‐centered 3 × 3 × 1 k‐mesh were used for the surface structures. A Gaussian smearing of 0.1 eV was used for all geometry optimizations. Dipole corrections were included with a sufficiently large cell vacuum of ∼40 Å to ensure planar average convergence [70, 71]. Bader charge analysis was performed to obtain accurate charge partitioning on the DFT‐optimized molecule‐surface structures using high‐precision DFT single‐point calculations with a fine FFT mesh of 260 × 260 × 1400 points. Such ab initio computational techniques have been applied to recent studies of molecular passivation of perovskite halide surfaces [72, 73, 74].

### 4.6 Optical Simulation

The light‐emitting behavior of PeNPLs were calculated with transfer‐matrix formalism based on the optical model previously reported in Refs. [75, 76]. The device structure consists of glass (*n* = 1.50)/ITO (*n* = 1.9, 170 nm)/PEDOT:PSS (*n* = 1.5, 30 nm)/Poly‐TPD (*n* = 1.6, 20 nm)/PeNPLs (20 nm)/TPBI (*n* = 1.7, 20 nm)/ZADN (*n* = 1.7, 40 nm)/Al (100 nm), based on the refractive indices and radiation spectrum shown in Figure 2b and Figure S33. The dipoles were assumed to be uniformly distributed over the perovskite layer, while the relative fractions of vertical and horizontal orientation have been varied. The imaginary part of the refractive index, *k*, was calculated from the absorption coefficient of the films. The extinction coefficient *k* was calculated using the relation *k*  =  αλ/4π, where λ is the wavelength.

## Author Contributions

J.K., W.H.J., J.Y. and R.L.Z.H. conceived the ideas behind this work. J.K. and W.H.J. performed formal analysis under the supervision of M.H.S., B.R.L., and R.L.Z.H., A.N.A and V. performed DFT calculations under the supervision of M.S.I. D.K. and D.L performed confocal PL microscopy. Y.‐T.H. and Y.W. performed transient absorption spectroscopy under the supervision of A.R. C.‐Y.C. and J.K performed the TEM measurements. X.S., A.S. and H.J.S. performed optical analysis, S.Y.B. performed XRD. J.Y. performed XPS. Y.I.K. performed FTIR. B.J. performed optical simulation under the supervision of C.C. J.K. and W.H.J. drafted the first version of the manuscript. J.Y., M.S.I., B.R.L., M.S. and R.L.Z.H. reviewed and revised the manuscript. This work was carried out under the supervision of B.R.L., M.H.S and R.L.Z.H. All the authors contributed to the discussion of the manuscript.

## Conflicts of Interest

The authors declare no conflicts of interest.

## Supporting information




**Supporting File**: adma74023‐sup‐0001‐SuppMat.pdf.

## Data Availability

The data that support the findings of this study are available from the Oxford Research Archive repository through the following link: https://dx.doi.org/10.5287/ora‐rjb2zz0q8.
