# Supplementary material for: Ligand Engineering for Precise Control of Ultrathin CsPbI3 Nanoplatelet Superlattices for Efficient Light‐Emitting Diodes
Source: Adv Mater. 2026 Jul 10;38(46):e74023. doi: 10.1002/adma.74023 (PMC13486141; doi:10.1002/adma.74023)
Supplement: Supplementary file 1 [file ADMA-38-e74023-s001.pdf]

Supporting Information for

Ligand Engineering for Precise Control of Ultrathin $CsPbI_3$ Nanoplatelet Superlattices for Efficient Light-Emitting Diodes

Jongbeom Kim[1,†], Woo Hyeon Jeong[2,3,†], Junzhi Ye[3,4]*, Allison Nicole Arber[5], Vikram[5], Donghan Kim[1], Yi-Teng Huang[3,6], Yixin Wang[3], Dongeun Kim[1], Dongryeol Lee[1], Chia-Yu Chang[3], Xinyu Shen[7], Sung Yong Bae[3], Ashish Gaurav[3], Ye In Kim[1], Jinkyu Yang[1], Boseung Je[8], Changsoon Cho[8], Akshay Rao[9], Henry J. Snaith[7], M. Saiful Islam[5]*, Bo Ram Lee[2]*, Myoung Hoon Song[1]* Robert L. Z. Hoye[3]*

[1]Department of Materials Science and Engineering, Ulsan National Institute of Science and Technology (UNIST), Ulsan, 44919 Republic of Korea

[2]School of Advanced Materials Science and Engineering, Sungkyunkwan University, Suwon, 16419, Republic of Korea

[3]Inorganic Chemistry Laboratory, University of Oxford, Oxford, OX1 3QR, United Kingdom

[4]Institute of Polymer Optoelectronic Materials and Devices, Guangdong Basic Research Center of Excellence for Energy & Information Polymer Materials, State Key Laboratory of Luminescent Materials and Devices, School of Materials Science and Engineering, South China University of Technology, Guangzhou 510640, China.

[5]Department of Materials, University of Oxford, Oxford, OX1 3PH, United Kingdom

[6]Graduate Institute of Photonics and Optoelectronics and Department of Electrical Engineering, National Taiwan University, Taipei 10617, Taiwan

[7]Clarendon Laboratory, Department of Physics, University of Oxford, Oxford, OX1 3PU United Kingdom

[8]Department of Materials Science and Engineering, Pohang University of Science and Technology (POSTECH), Pohang, 37673, Republic of Korea

[9]Cavendish Laboratory, University of Cambridge, Cambridge CB3 0US, United Kingdom

[†]These authors contributed equally.

*Correspondence: junzhiye1994@scut.edu.cn (J.Y.), saiful.islam@materials.ox.ac.uk (M.S.I.), brlee@skku.edu (B.R.L.), mhsong@unist.ac.kr (M.H.S.), robert.hoye@chem.ox.ac.uk (R.L.Z.H.)

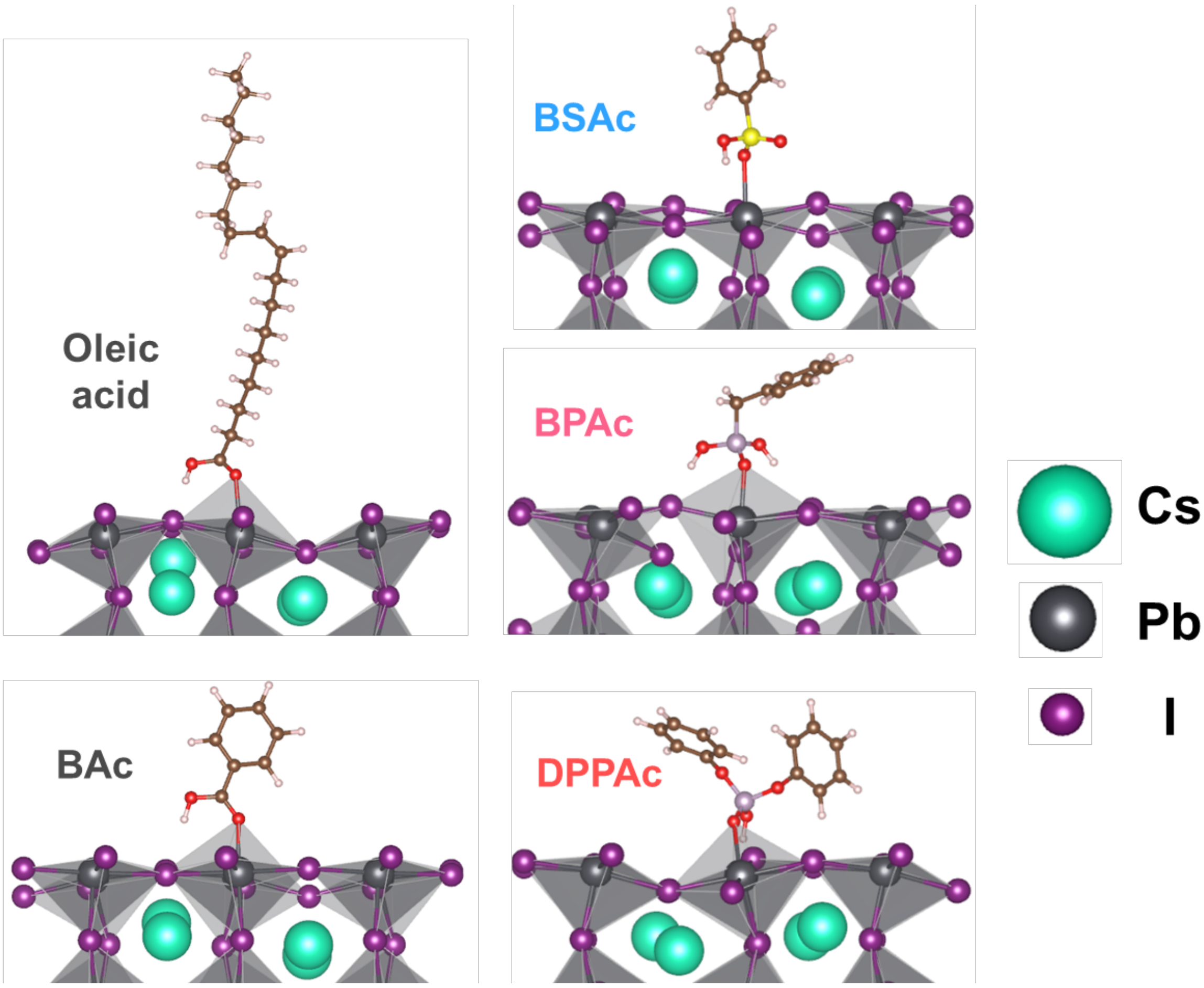

Figure S1. Optimized structure of each ligand on the (001) PbI_2 terminated perovskite surface for calculating ligand surface adsorption energy

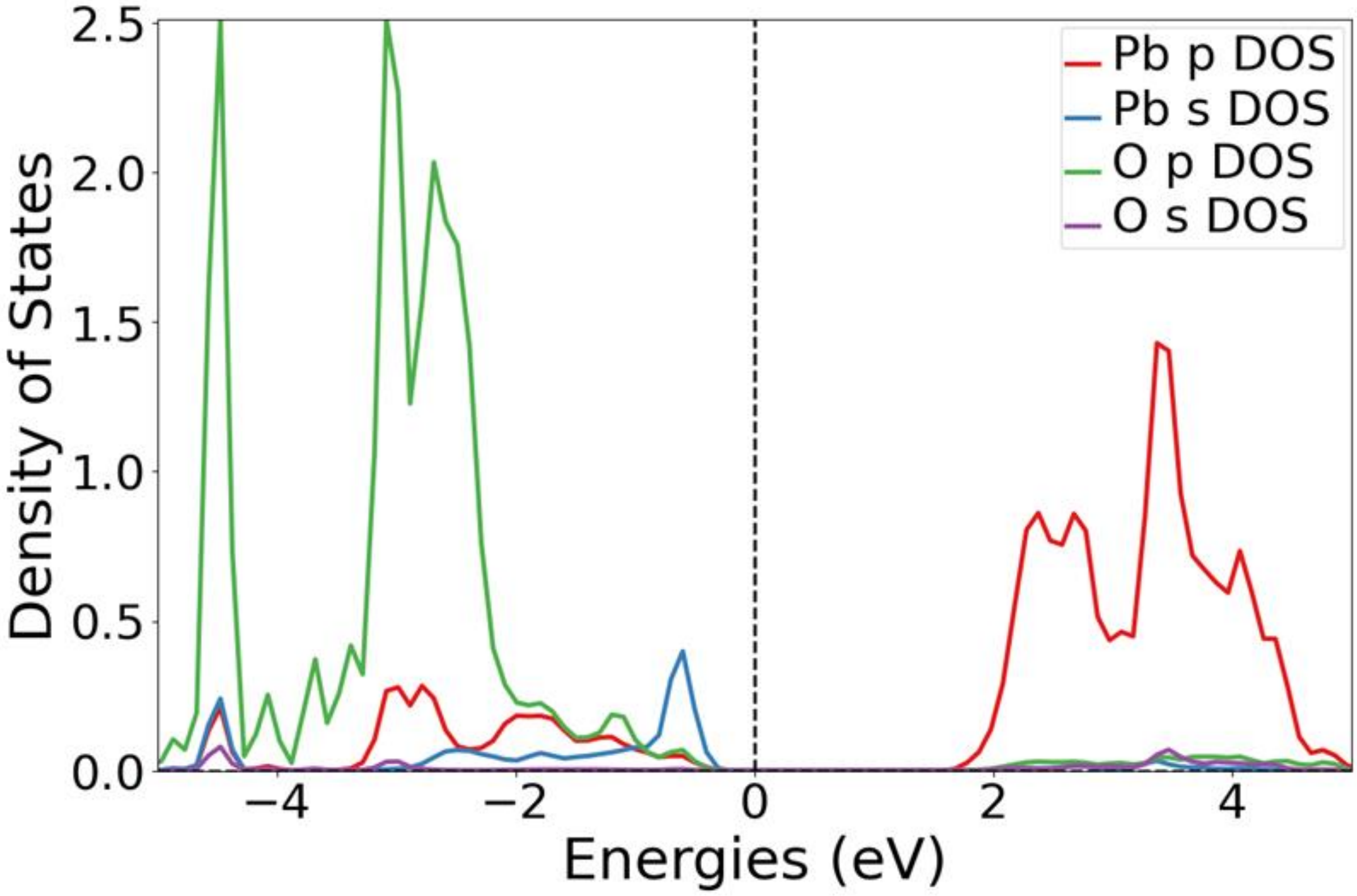

Figure S2. Projected density of states (PDOS) for Pb and O atoms involved in bonding at the BPAc-perovskite surface.

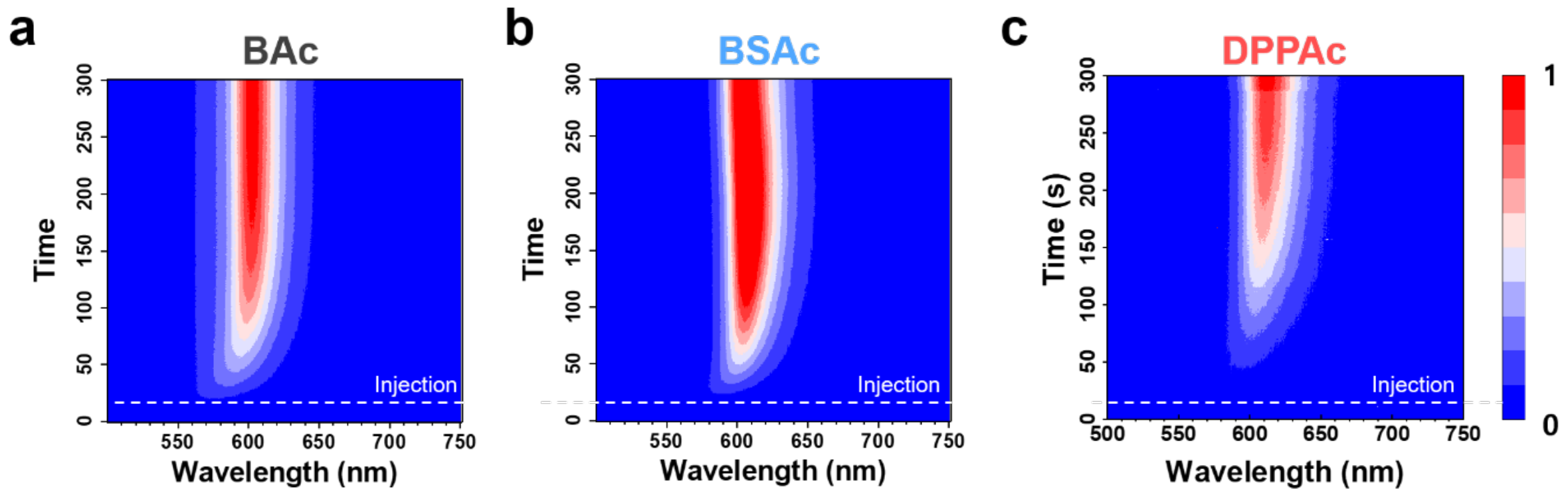

Figure S3. *In-situ* photoluminescence spectra of **a** BAc-, **b** BSAc- and **c** DPPAc-PeNPLs in colloidal solution recorded during synthesis under ambient air conditions, excited with a 405 nm CW laser (37.3 mW cm^{-2}). Dashed white line indicates when the Cs-oleate precursor was injected into the PbI_2-ligand solution.

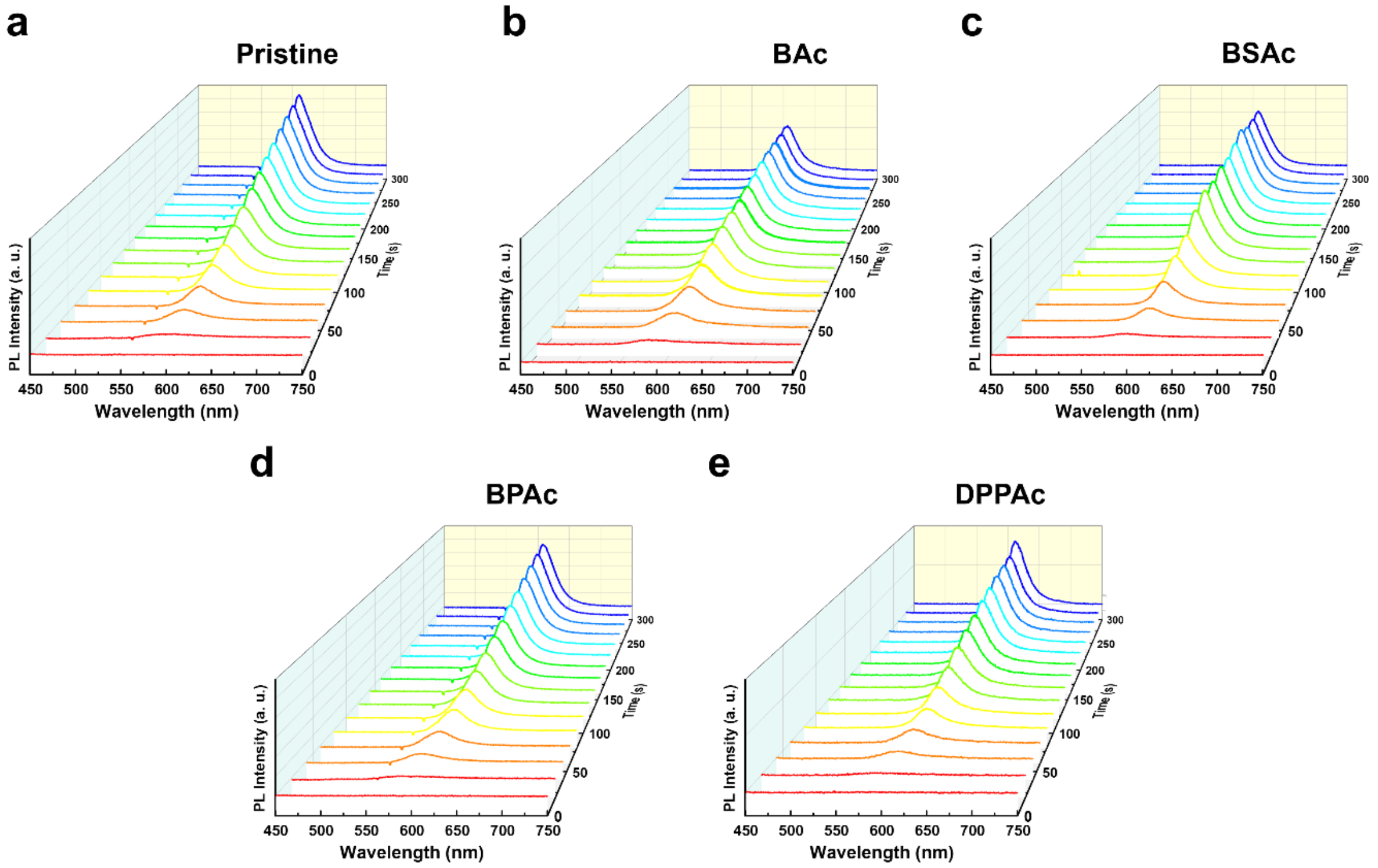

Figure S4. *In-situ* PL spectra during the formation of PeNPLs following the injection of the Cs-oleate into the PbI_2 solution. Spectra obtained at 20 s time intervals. Samples were measured in ambient air during synthesis.

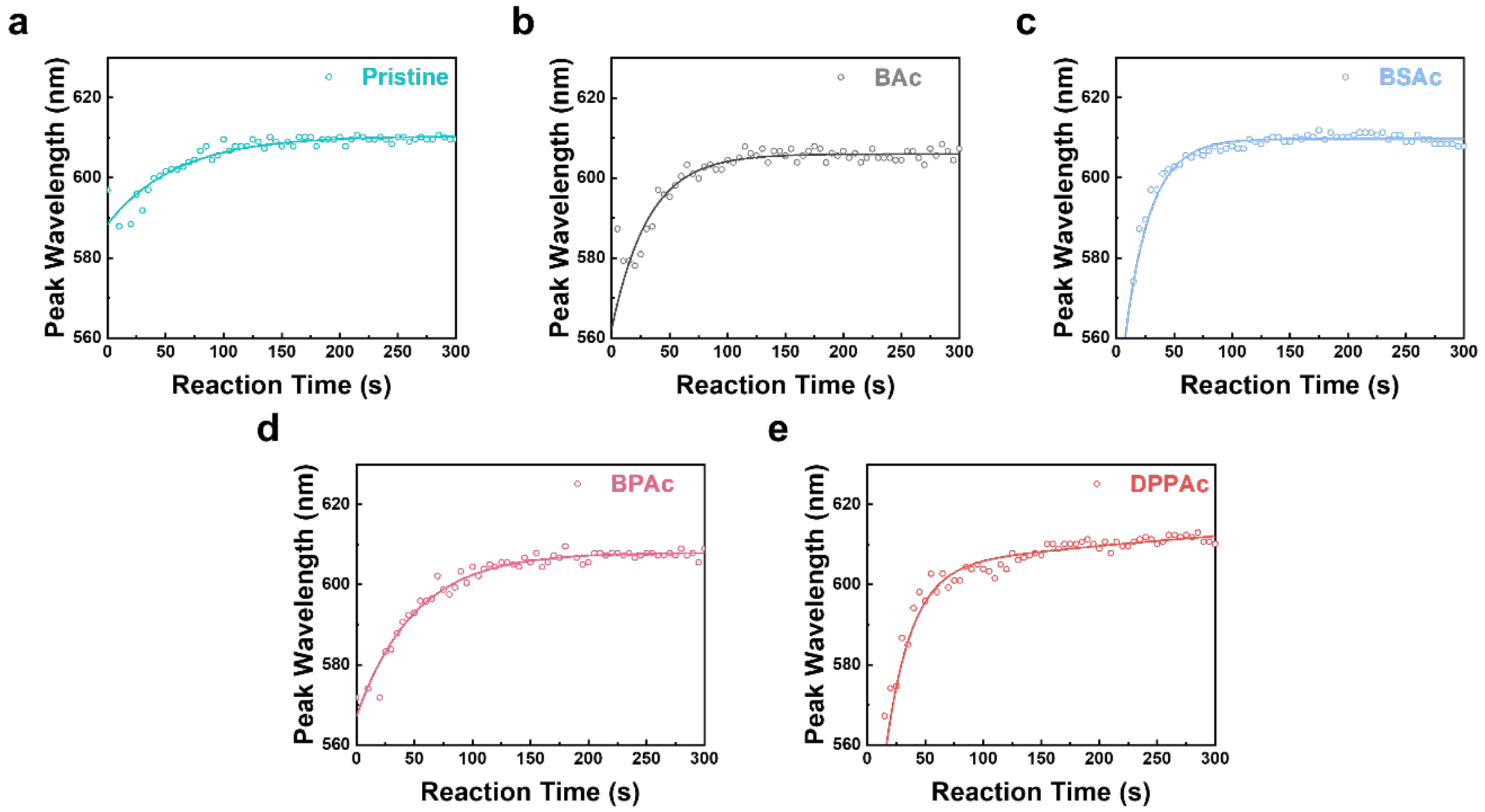

Figure S5. Time-resolved evolution of the PL peak wavelength during growth of **a** Pristine-, **b** BAc-, **c** BSAc- **d** BPAc- and **d** DPPAc-PeNPLs, with 5 s interval data collection.

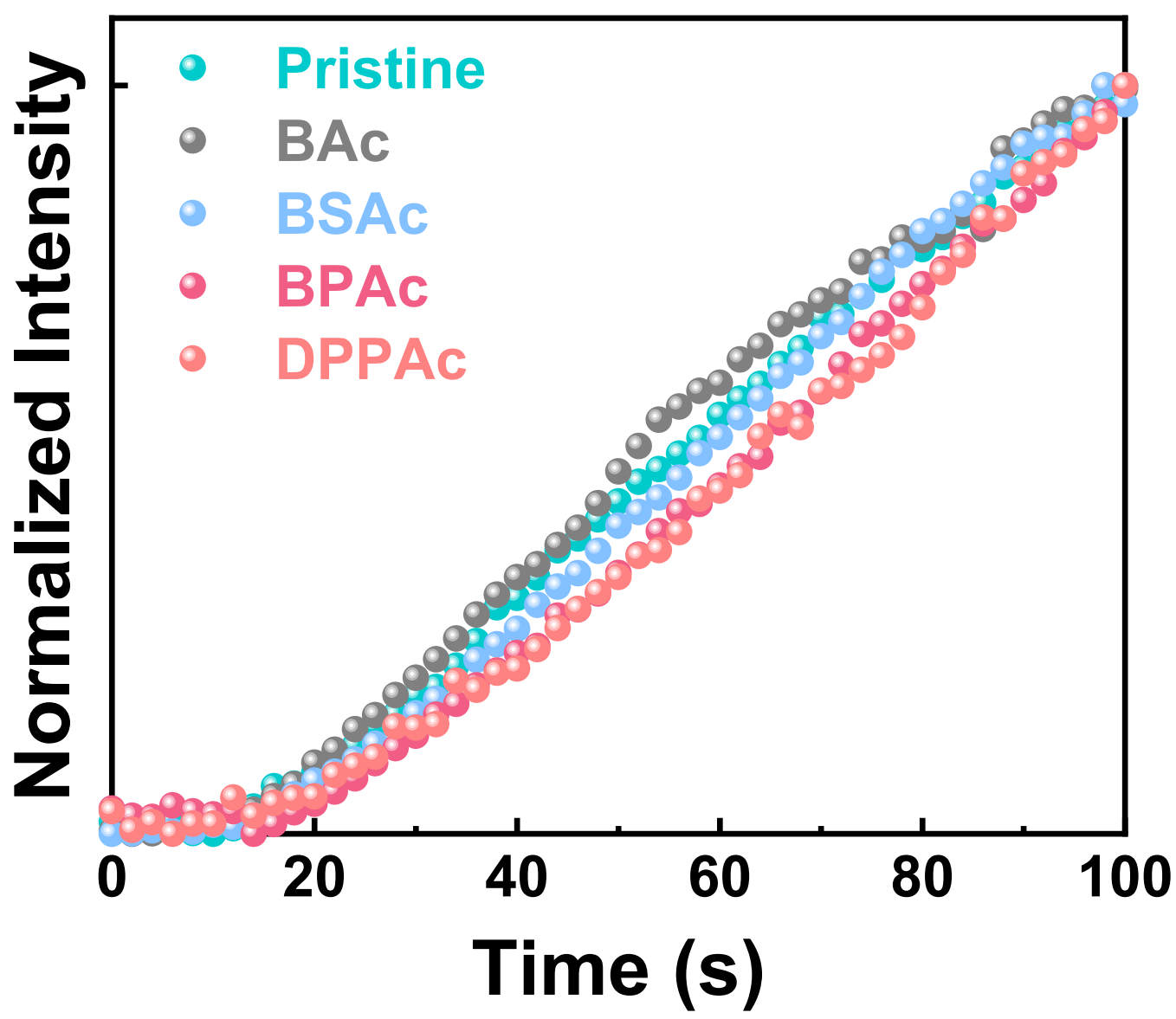

Figure S6. *In-situ* PL intensity changes of during synthesis of PeNPLs with different ancillary ligands.

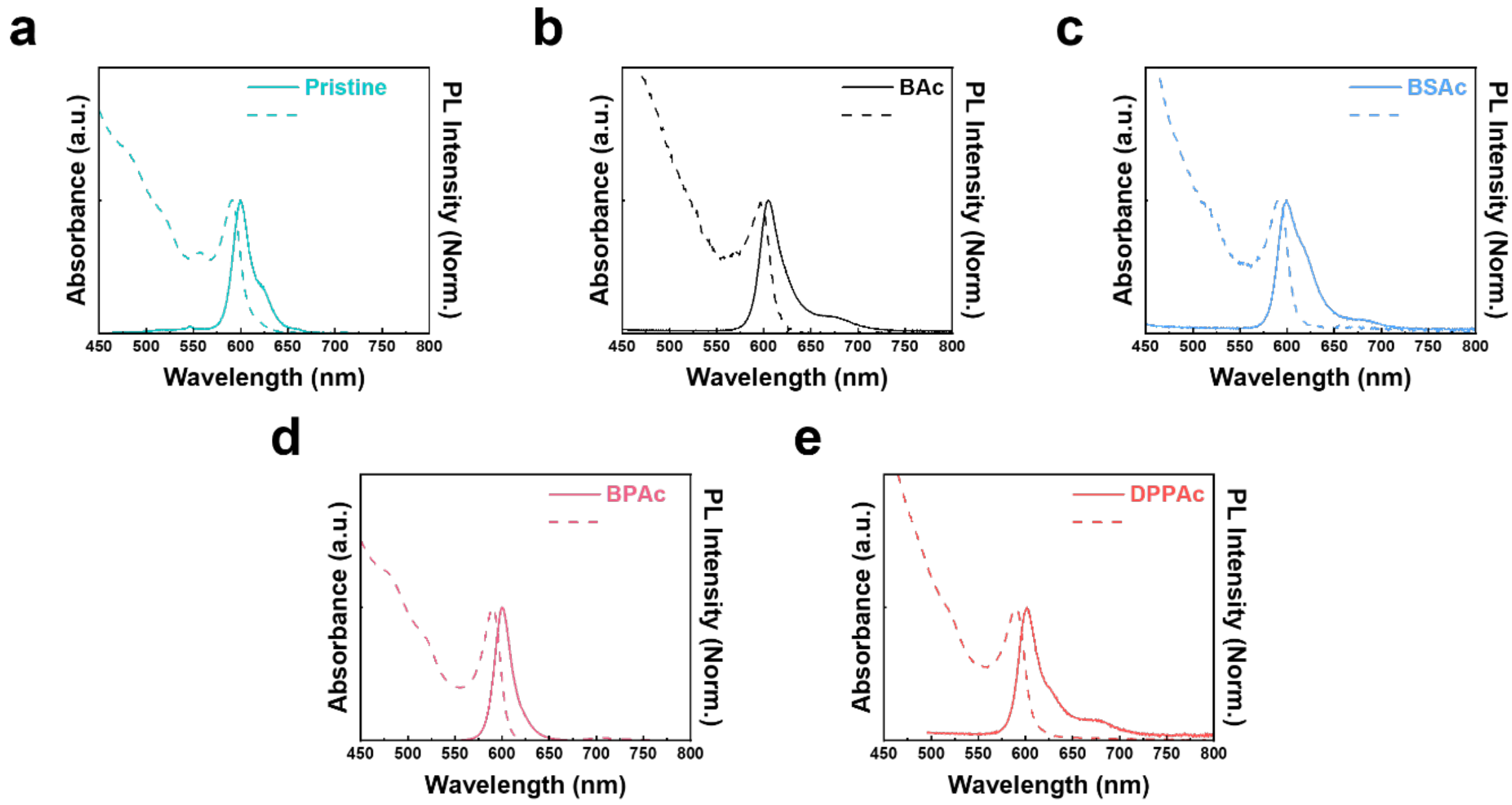

Figure S7. Ultraviolet-visible absorption (dashed lines) and photoluminescence (solid lines) spectra of **a** pristine-PeNPL, **b** BAc-PeNPL, **c** BSAc-PeNPL, **d** BPAc-PeNPL and **e** DPPAc-PeNPL dispersions.

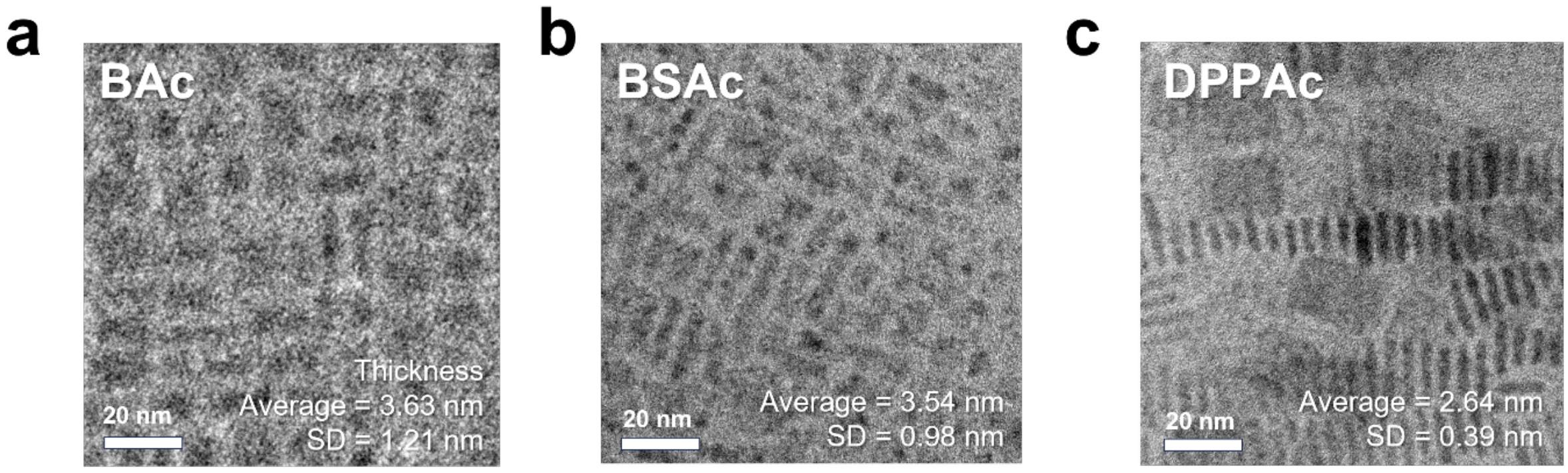

Figure S8. Transmission electron microscopy (TEM) image of **a** BAc-PeNPLs, **b** BSAc-PeNPLs and **c** DPPAc-PeNPLs. These PeNPLs were dispersed in hexane and drop-cast onto a Cu TEM grid. These samples, prepared by drop casting, are useful for measuring the size of PeNPLs, but are not representative of the orientation of PeNPLs in spin coated films, for which GIWAXS provides a more accurate measure.

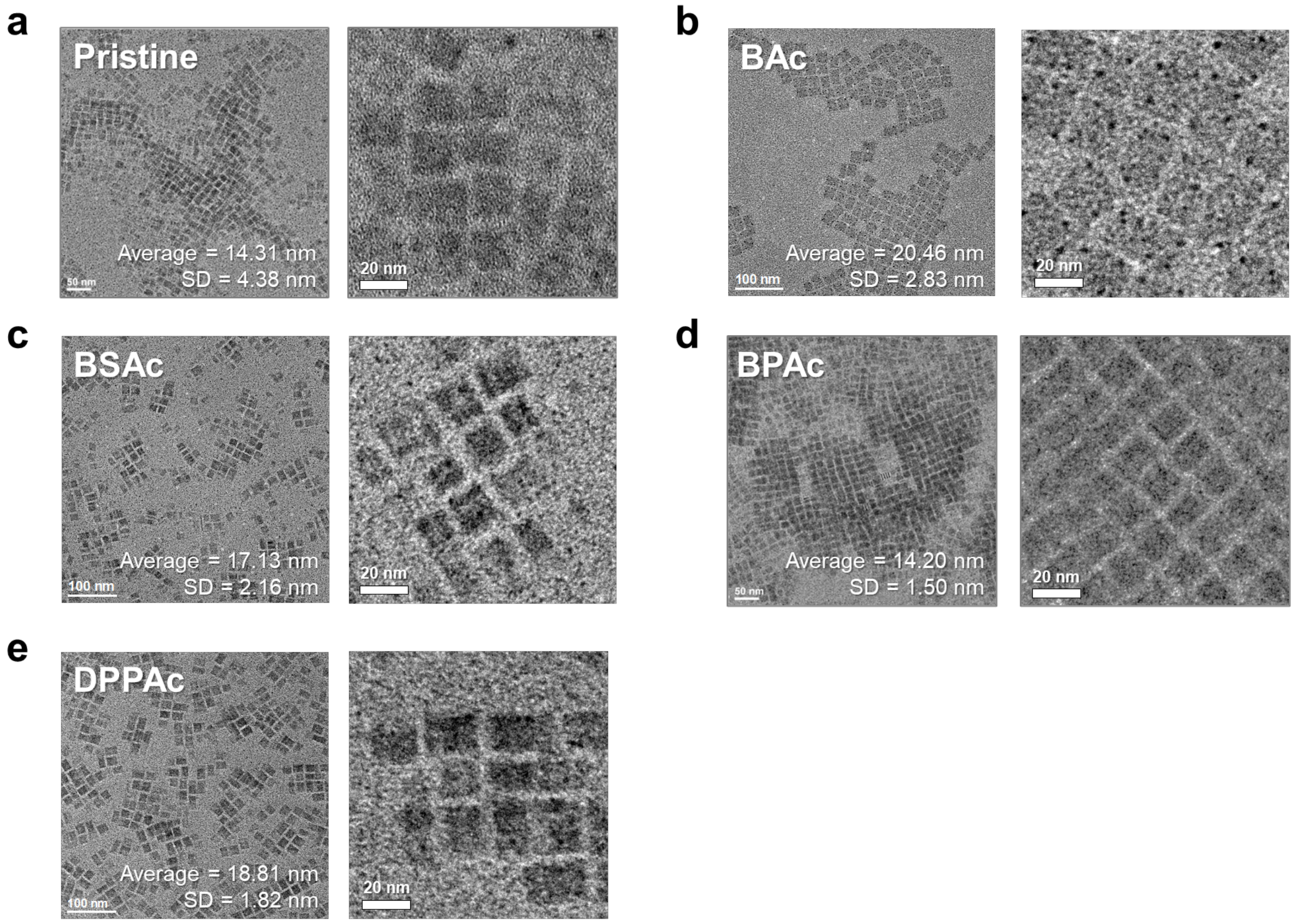

Figure S9. Transmission electron microscopy (TEM) image of **a** pristine-, **b** BAc-, **c** BSAc, **d** BPAc- and **e** DPPAc-PeNPL dispersions. These PeNPLs were dispersed in an octane solvent, and drop-cast onto a Cu TEM grid.

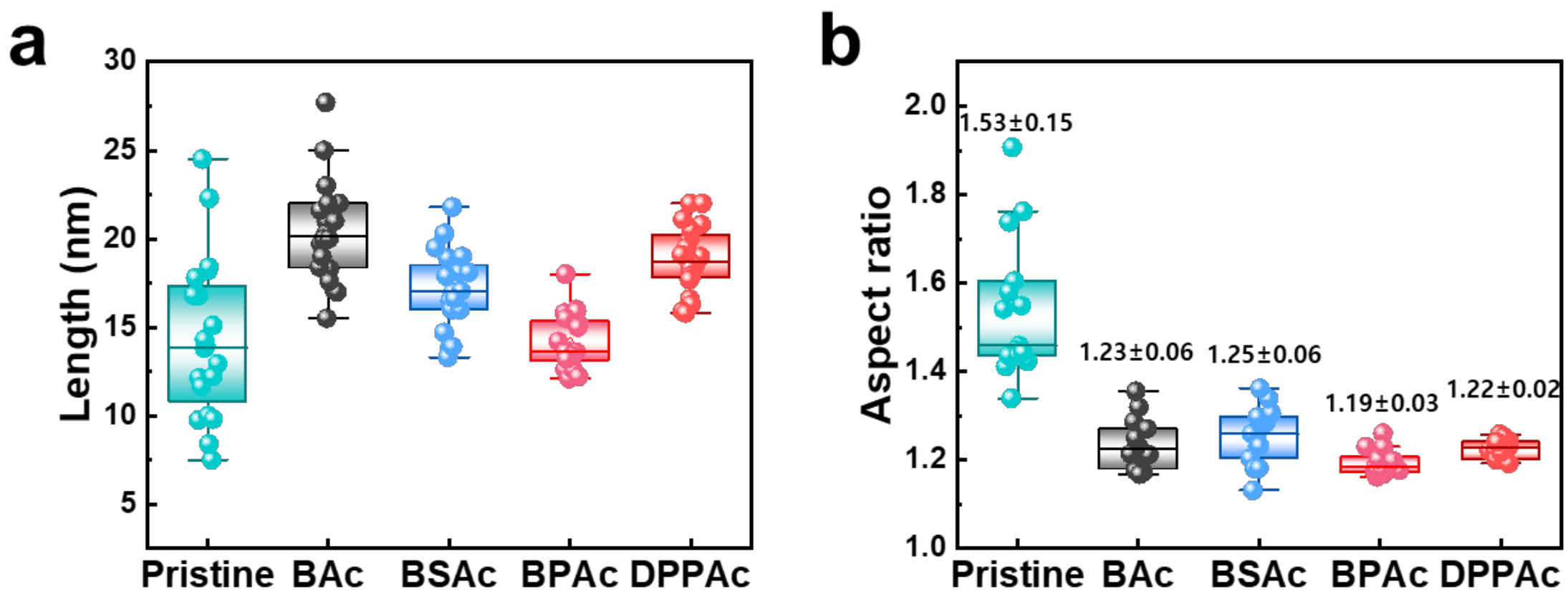

Figure S10. a Size distribution and **b** aspect ratio histogram of each PeNPLs determined from TEM measurements from Figure S8.

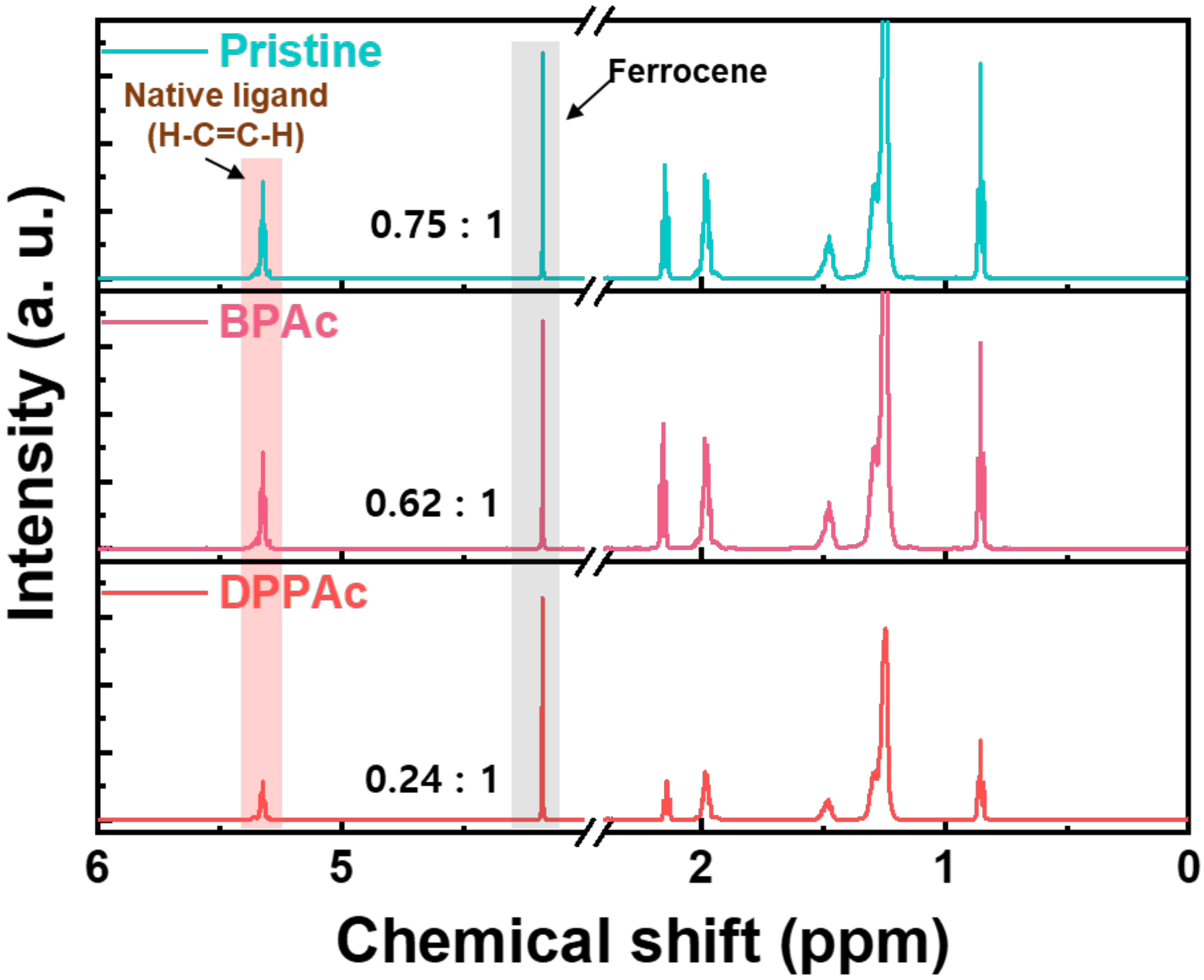

Figure S11. ^{1}H-NMR results of pristine-, BPAc- and DPPAc-PeNPLs. Quantitative integration ratio of peaks for ferrocene and native ligand (alkene) is calculated.

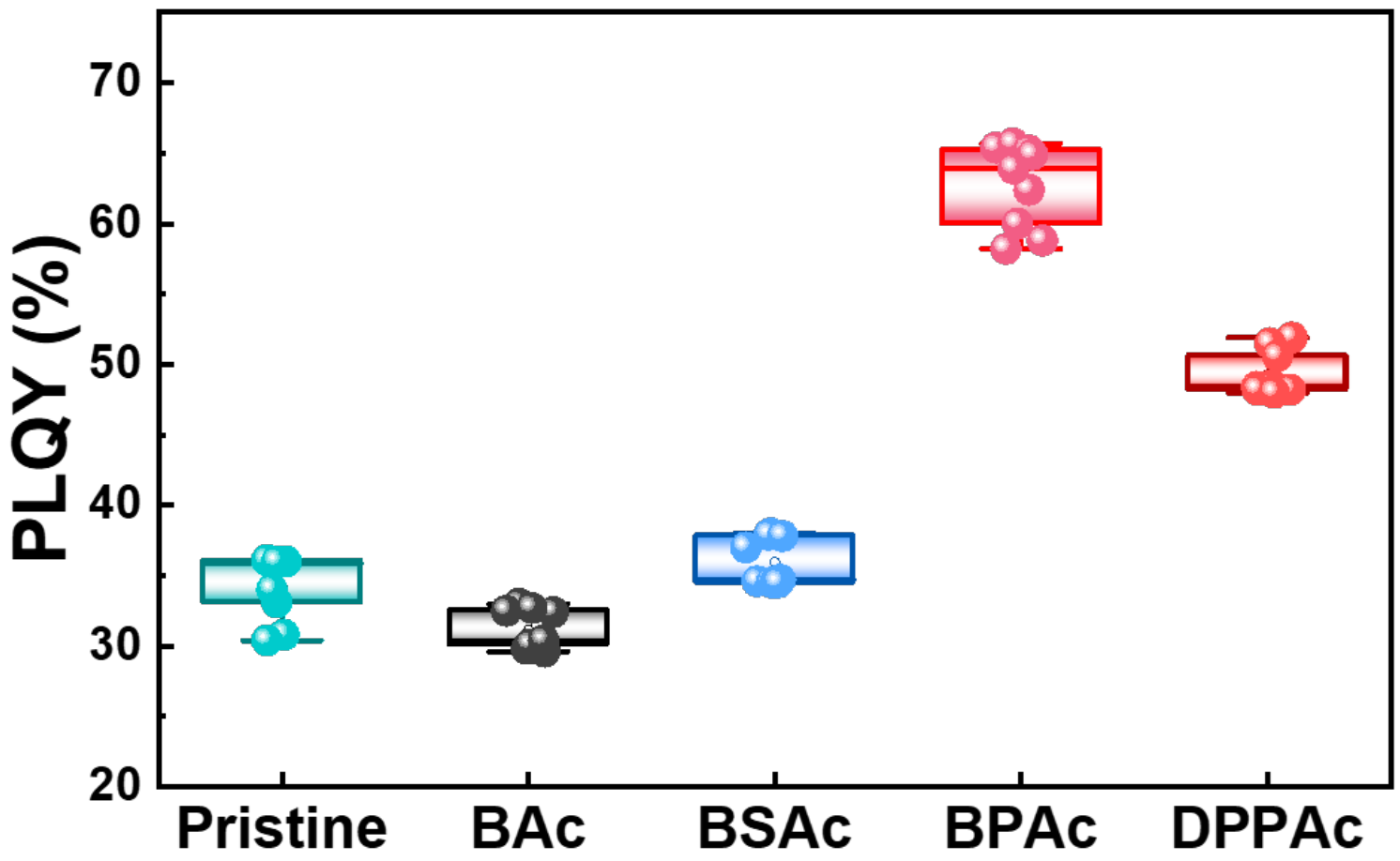

Figure S12. PLQY distributions of Pristine-, BAc-, BSAc-, BPAc and DPPAc-PeNPL colloidal solutions, excited with a 400 nm wavelength Xenon lamp. Samples were measured using a Quantaurus-QY Absolute PL quantum yield spectrometer (HAMAMATSU).

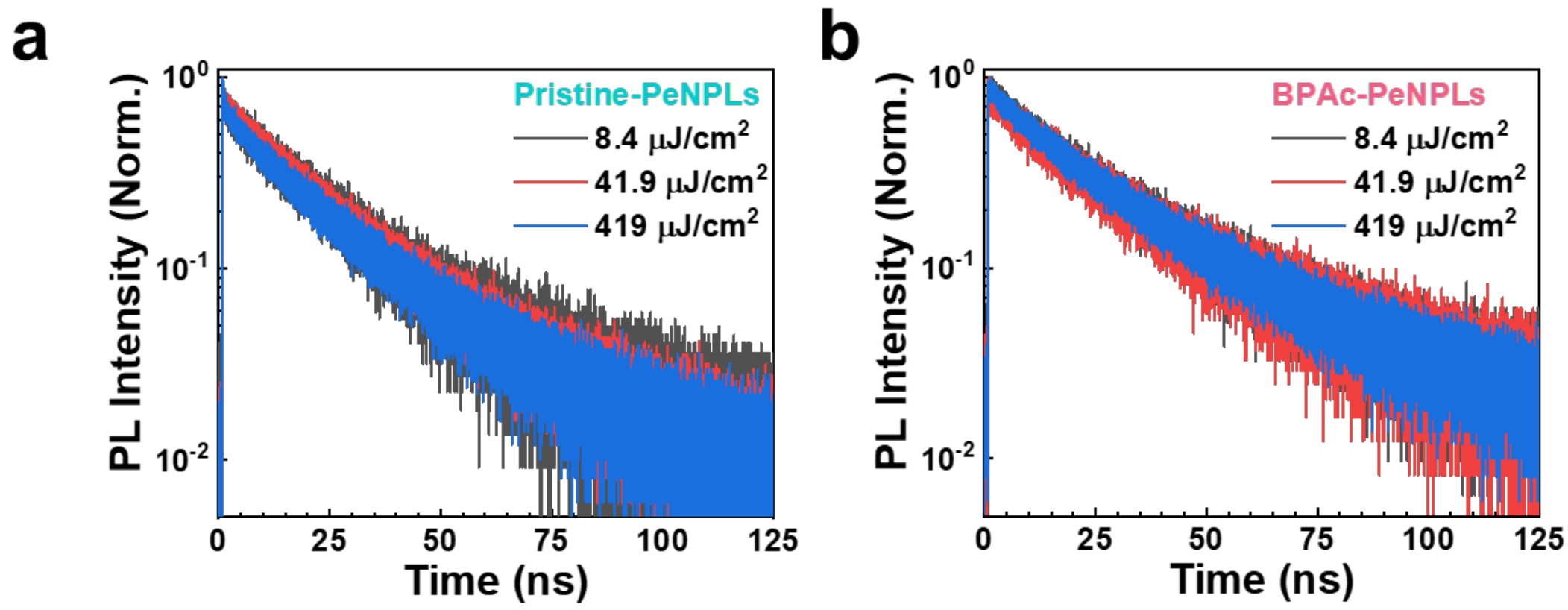

Figure S13. Excitation fluence-dependent time-resolved photoluminescence decay curves of **a** pristine-PeNPL, **b** BPAc-PeNPL colloidal solutions excited with a 400 nm wavelength pulsed laser.

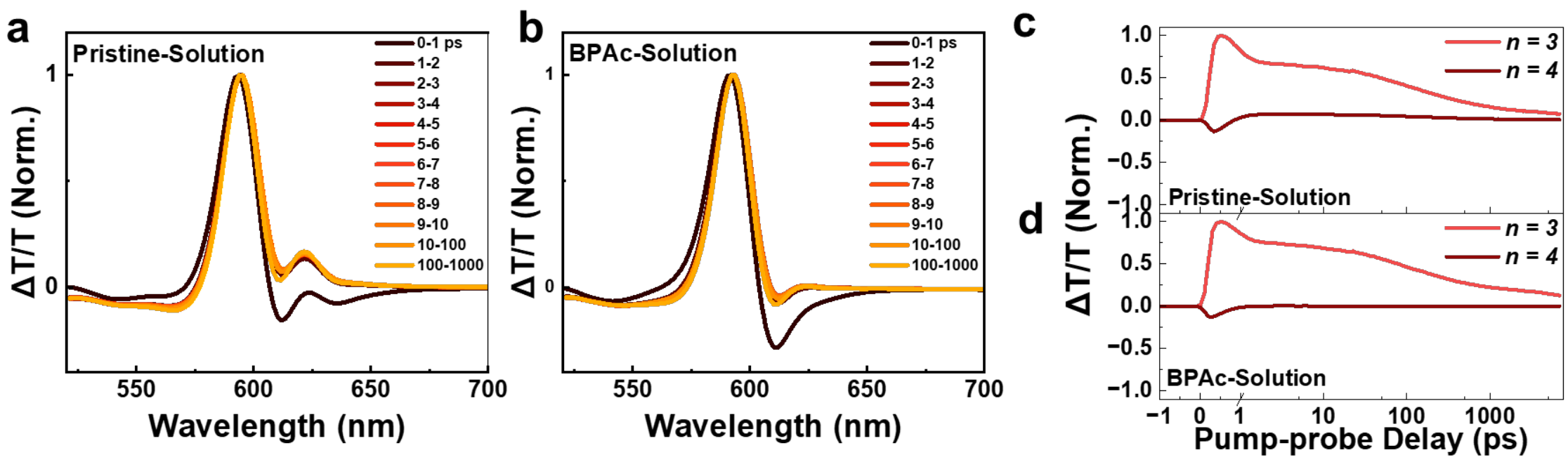

Figure S14. Transient absorption spectroscopy measurements of **a** pristine-PeNPL solution, and **b** BPAc-PeNPL solution excited with a 400 nm wavelength laser (3.37 μJ cm^{-2} fluence). **c** Decay curves of pristine-PeNPL and **d** BPAc-PeNPL colloidal solutions. n=3 refers to three monolayer NPLs probed from 580 nm to 600 nm. n=4 refers to four monolayer NPLs probed from 610 nm to 630 nm. Positive signals represent the ground states bleach (GSB) signals. Each kinetics trace is normalized to the maximum GSB for n = 3.

Note S1. Nuclear Magnetic Resonance Analysis of Ligand–Surface Interactions in PeNPLs

Experimental evidence for BPAc coordination on the surface of the PeNPLs is further supported by solution-phase nuclear magnetic resonance (NMR) measurements. In the ^{1}H-NMR spectrum (Figure S15), BPAc-treated PeNPLs exhibit characteristic resonances at approximately 7.3 ppm and 2.95 ppm, corresponding to the aromatic and methylene protons of BPAc, confirming the presence of BPAc molecules bound to the nanocrystal surface [1]. Furthermore, ^{207}Pb NMR spectra (Figure S16a) reveal a pronounced chemical-shift change relative to pristine PeNPLs, indicating a modified Pb coordination environment upon BPAc binding [2]. Further evidence of direct phosphonate coordination is provided by ^{31}P NMR measurements (Figure S16b). Compared with the BPAc ligand spectra, the phosphorus resonance of BPAc-PeNPLs exhibits a clear chemical shift change, indicating an altered phosphorus chemical environment arising from coordination of the phosphonic acid group with surface Pb sites.

Taken together, these NMR results provide direct evidence of strong ligand binding to the perovskite surface, consistent with the binding energies predicted by the DFT calculations in **Figure 1b**.

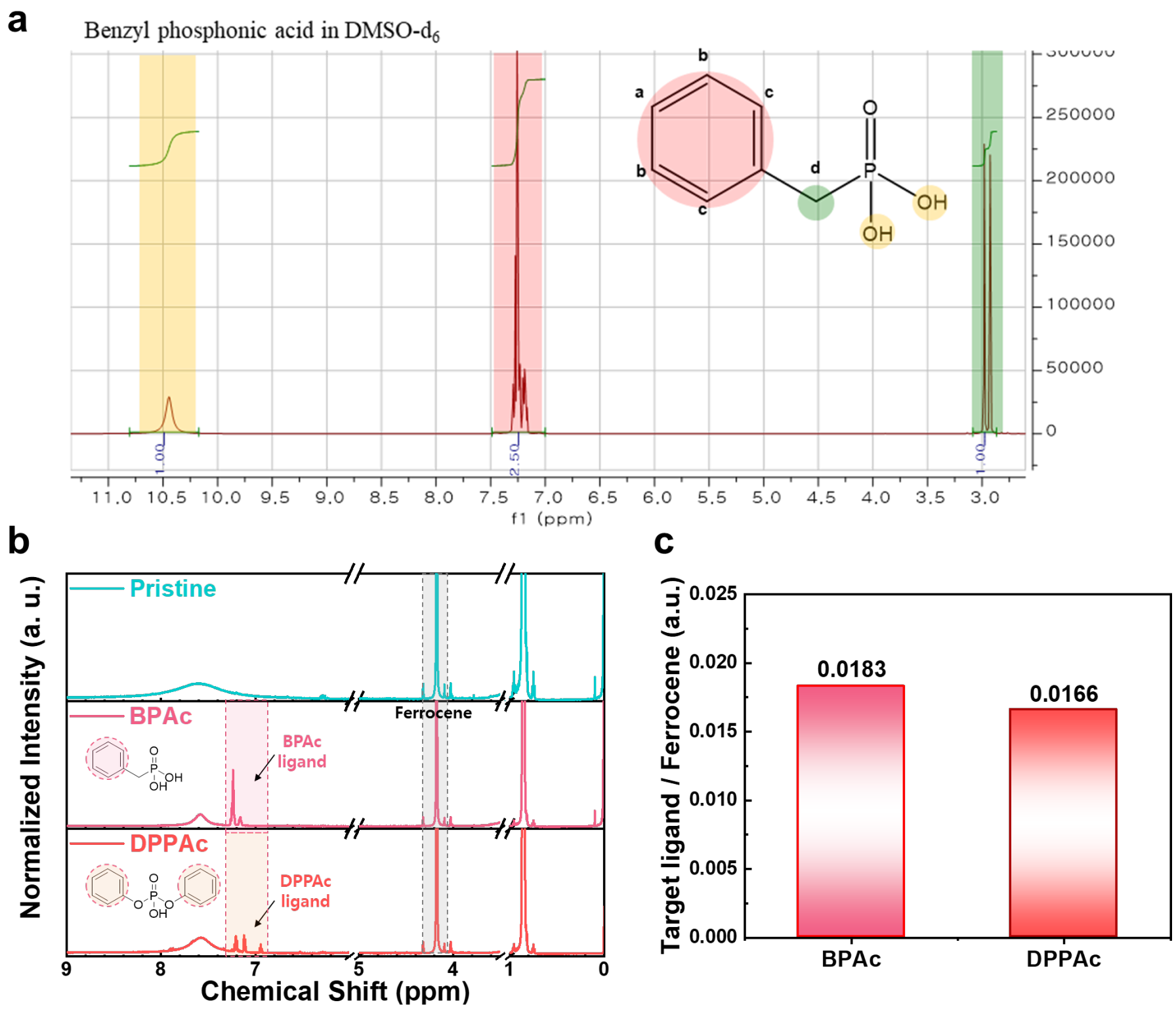

Figure S15. a Chemical structure and ^{1}H NMR spectra of benzylphosphonic acid (BPAc) dissolved in DMSO-d_6. **b** ^{1}H NMR spectra of pristine-, BPAc and DPPAc-PeNPL dissolved in DMSO-d_6. **c** Ligand concentrations calculated by comparison of integrated peak areas. Ferrocene is used as the internal standard for quantitative comparison of the ratios of the ligand densities on the PeNPL surfaces.

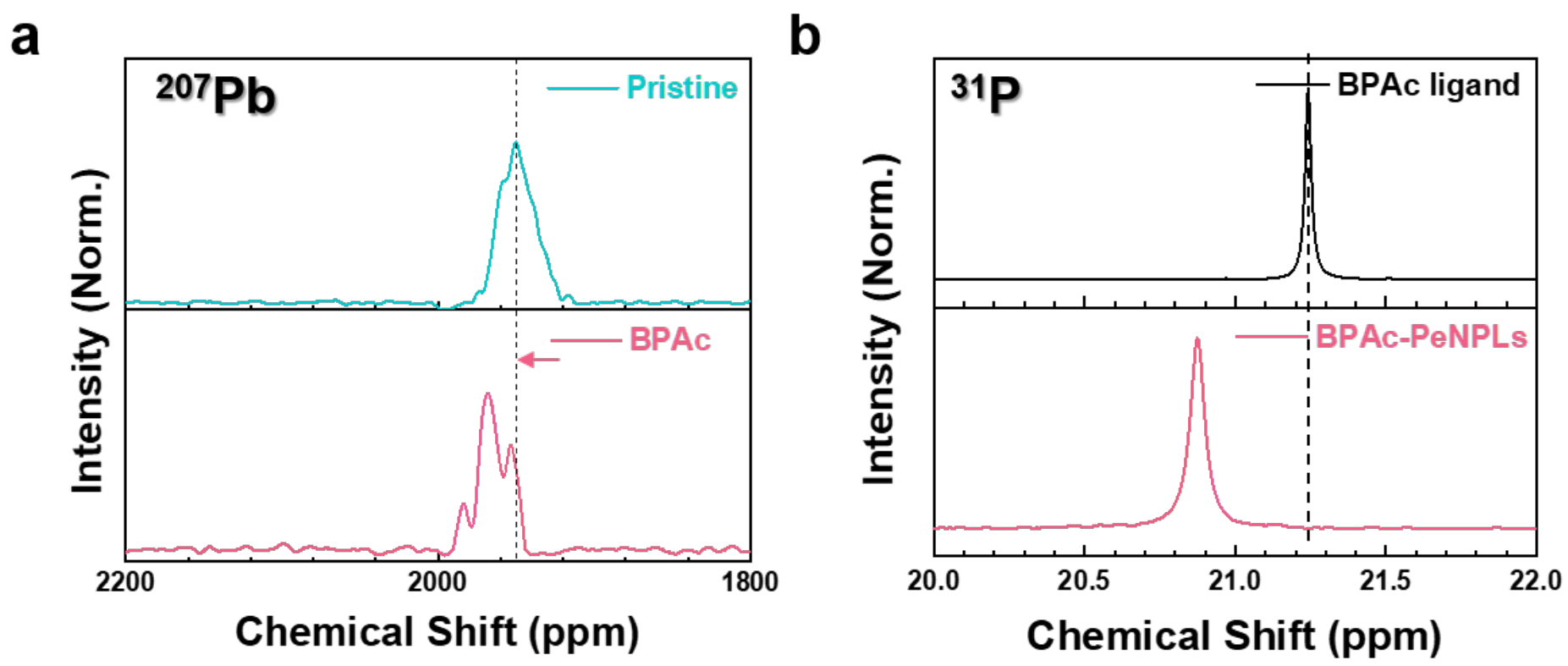

Figure S16. a ^{207}Pb NMR spectra of pristine- and BPAc-PeNPLs dissolution in DMSO-d_6. **b** ^{31}P NMR spectra of BPAc ligand and BPAc-PeNPL dissolution in DMSO-d_6.

Note S2. XPS and FTIR Evidence for BPAc Retention in PeNPL Thin Films

To further verify that BPAc remains associated with the purified nanocrystal surface and persists after film formation, additional X-ray photoelectron spectroscopy (XPS) and Fourier-transform infrared (FTIR) measurements were performed. As shown in **Figure S17**, XPS survey and core-level spectra reveal clear differences between pristine- and BPAc-PeNPL thin films. In particular, the Pb 4f core level peaks exhibit a binding-energy shift upon BPAc incorporation, indicating modification of the Pb chemical environment arising from ligand coordination. By contrast, the Cs 3d and I 3d features remain largely unchanged, suggesting that the primary interaction occurs through Pb surface sites, consistent with the proposed Lewis base coordination mechanism.

Further support is provided by FTIR spectra (**Figure S18**), where BPAc-PeNPL thin films exhibit vibrational signatures associated with the benzene ring and phosphonate-containing surface species that are absent or significantly weaker in pristine films. The persistence of these features after purification and film formation supports retention of BPAc ligands in the final PeNPL thin films.

Together with the NMR results in Note S1, these measurements provide complementary evidence that BPAc remains bound to the PeNPL surface after purification and contributes to persistent defect passivation in the final films.

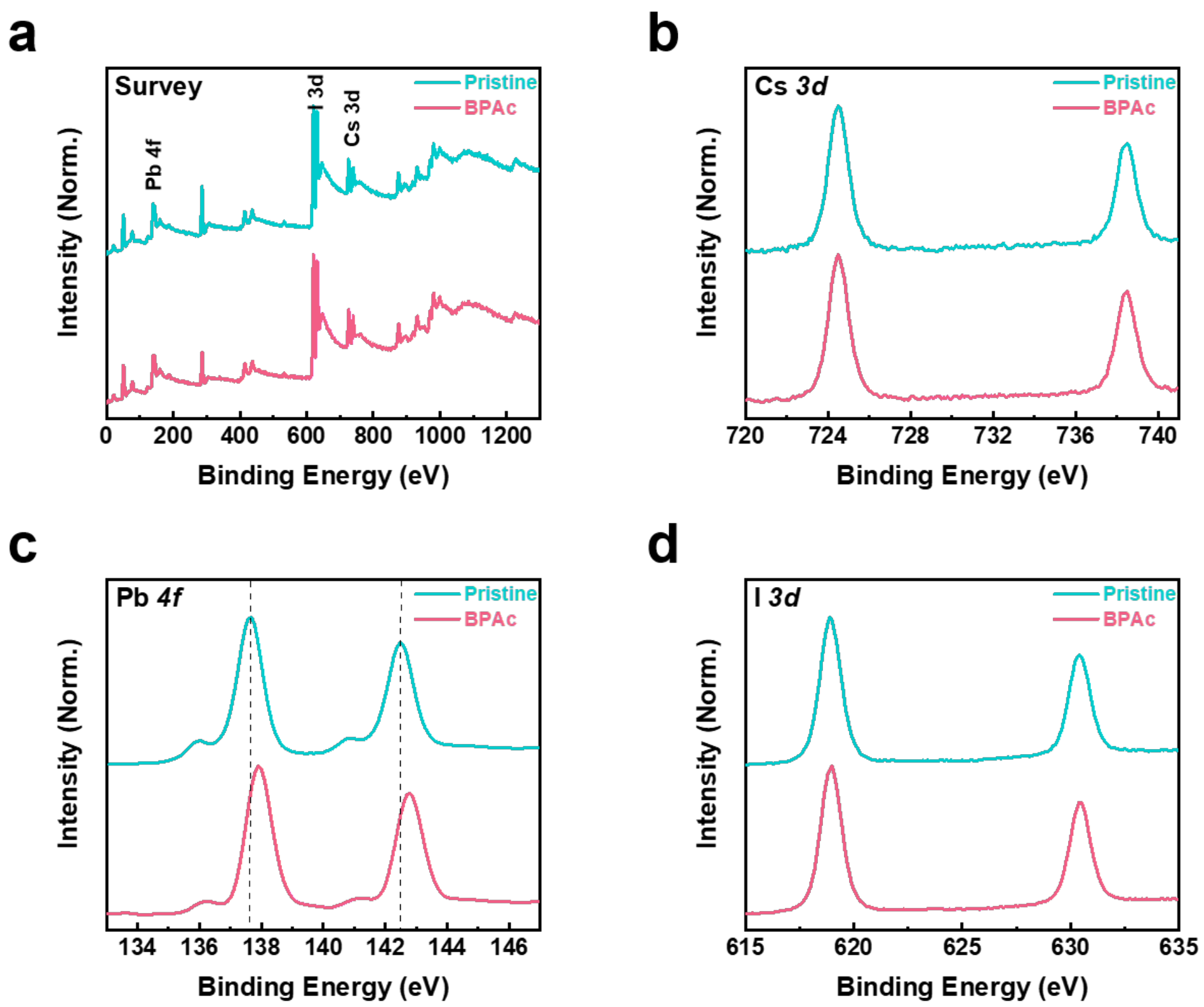

Figure S17. **a** Survey XPS spectra, **b** Cs 3d, **c** Pb 4f and **d** I 3d core level spectra of Pristine- and BPAc-PeNPLs.

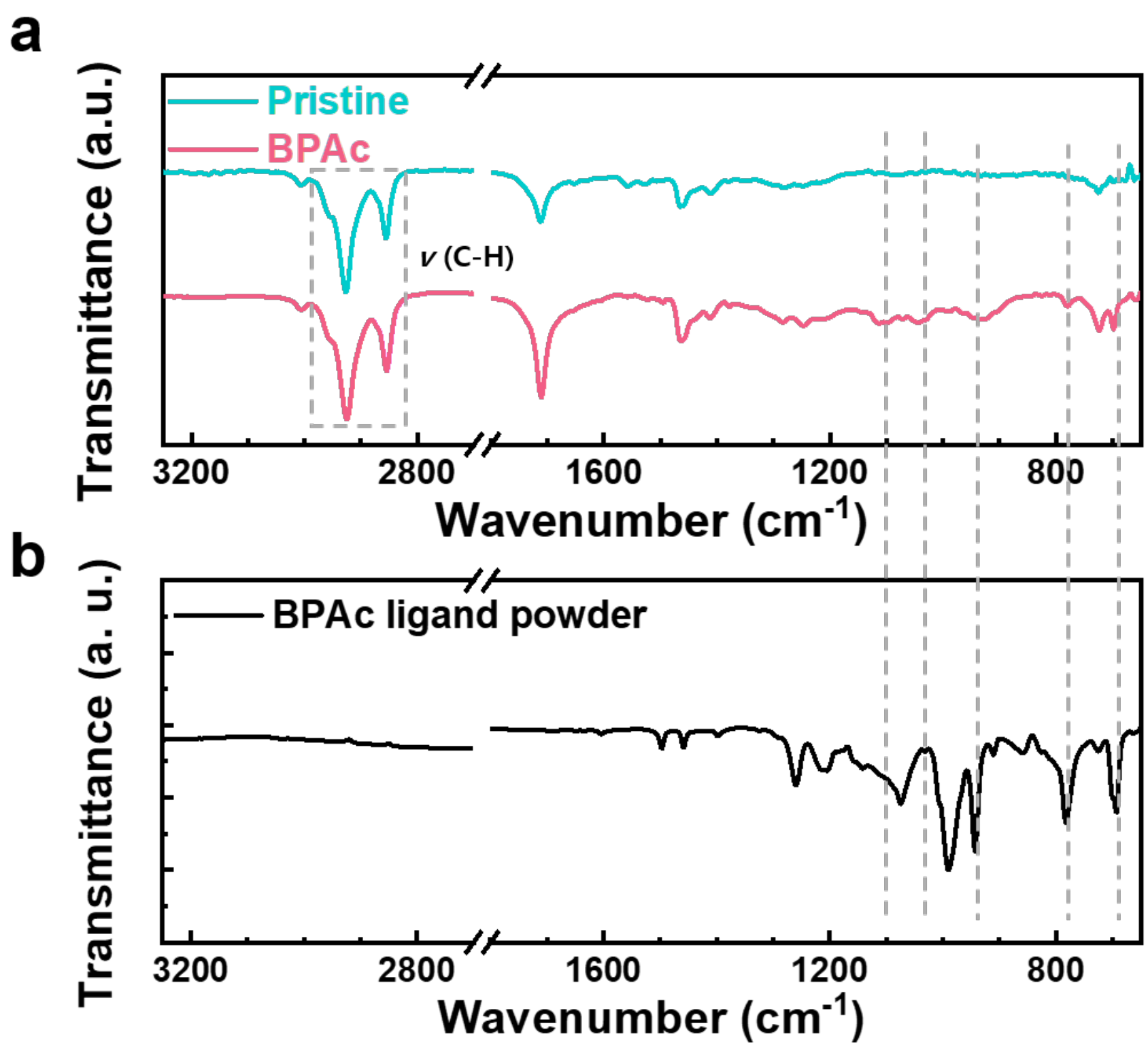

Figure S18. Fourier-transform infrared spectroscopy spectra of **a** Pristine- and BPAc-PeNPLs powder, **b** BPAc ligand powder.

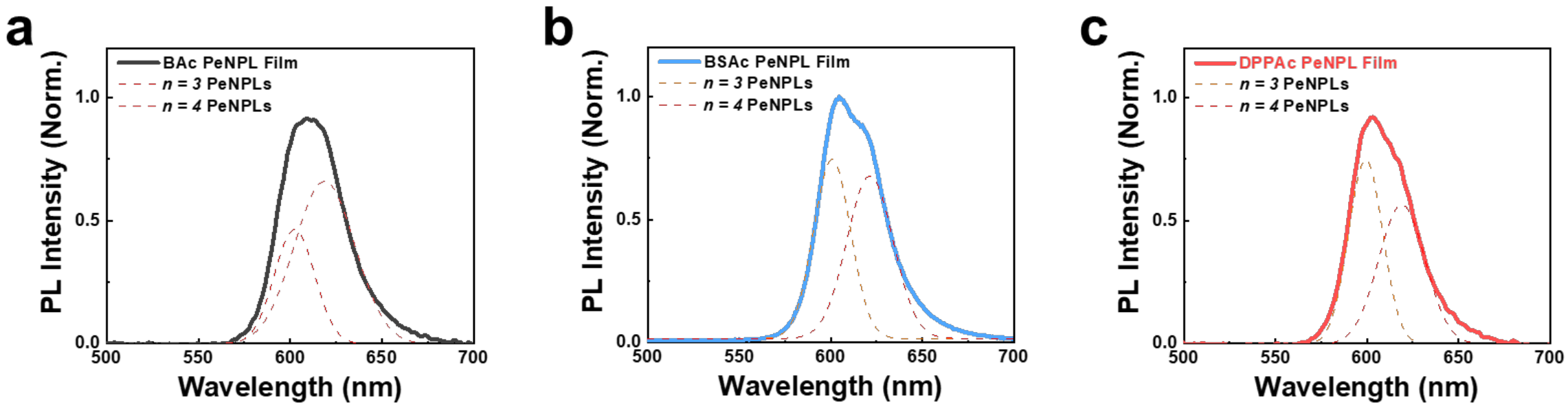

Figure S19. PL spectra of **a** BAc-, **b** BSAc- and **c** DPPAc-PeNPL thin films showing contributions from 3ML and 4ML components.

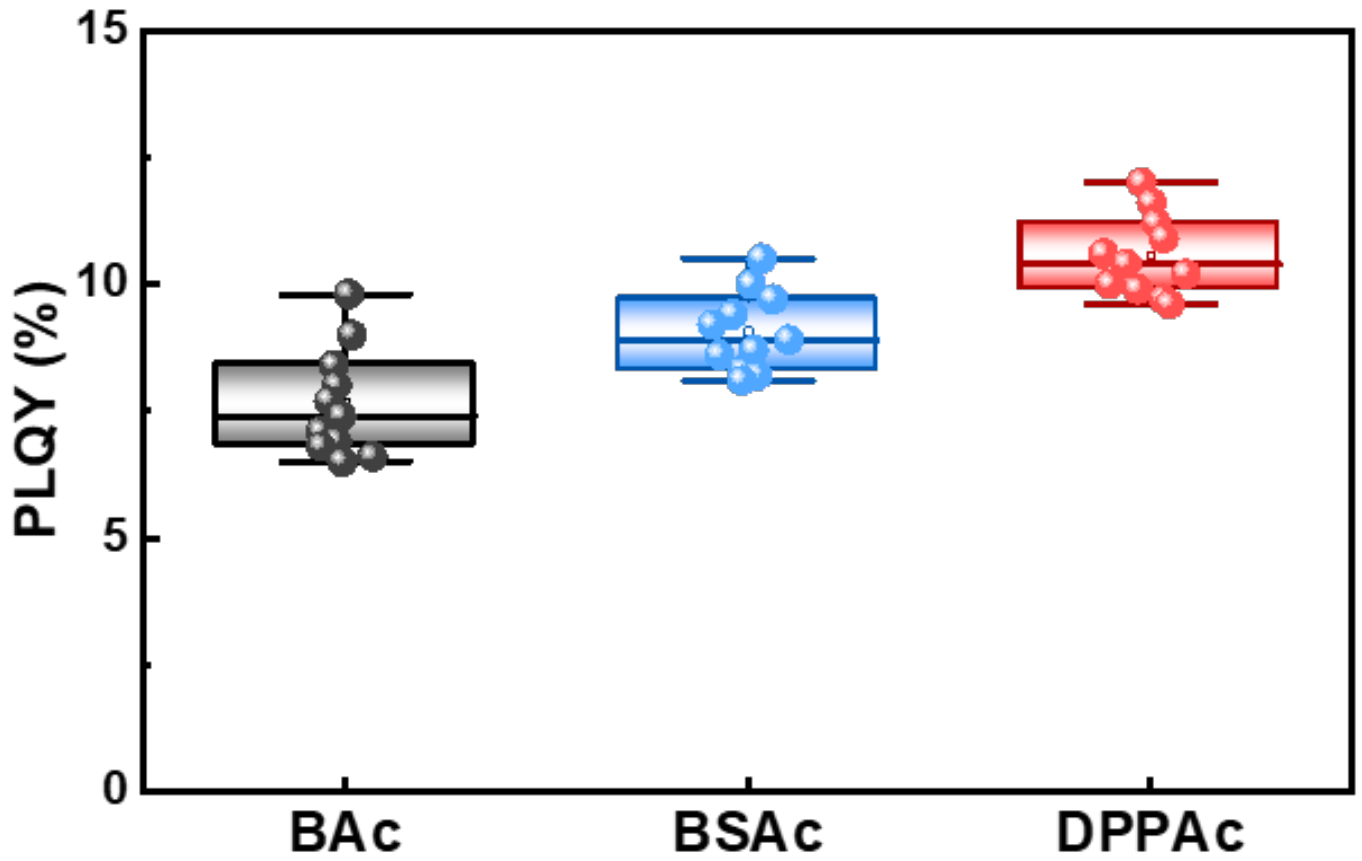

Figure S20. PLQY distributions of BAc-, BSAc- and DPPAc-PeNPL thin films, excited with a 400 nm wavelength Xenon lamp. Samples were measured using a Quantaurus-QY Absolute PL quantum yield spectrometer (HAMAMATSU). PLQY distributions of films based on pristine and BPAc-PeNPL shown in Figure 2c of the main text.

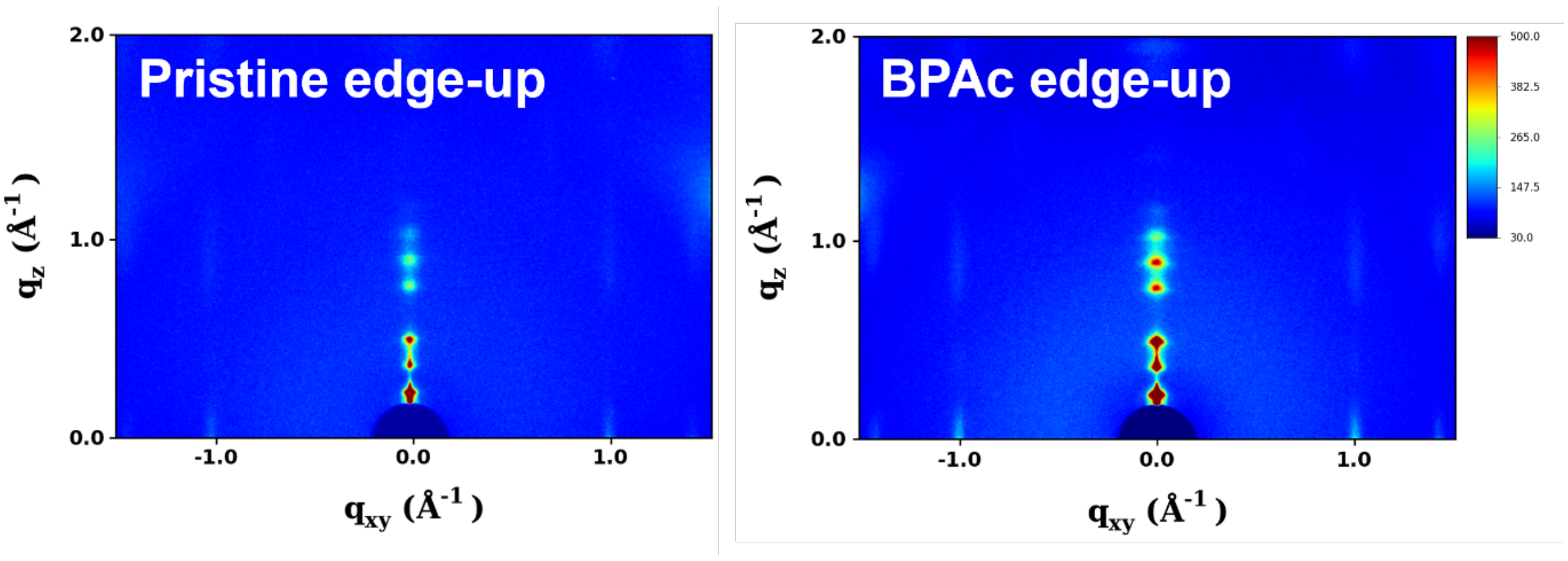

Figure S21. Grazing-incidence wide-angle X-ray scattering (GIWAXS) patterns of edge-up oriented PeNPL thin films.

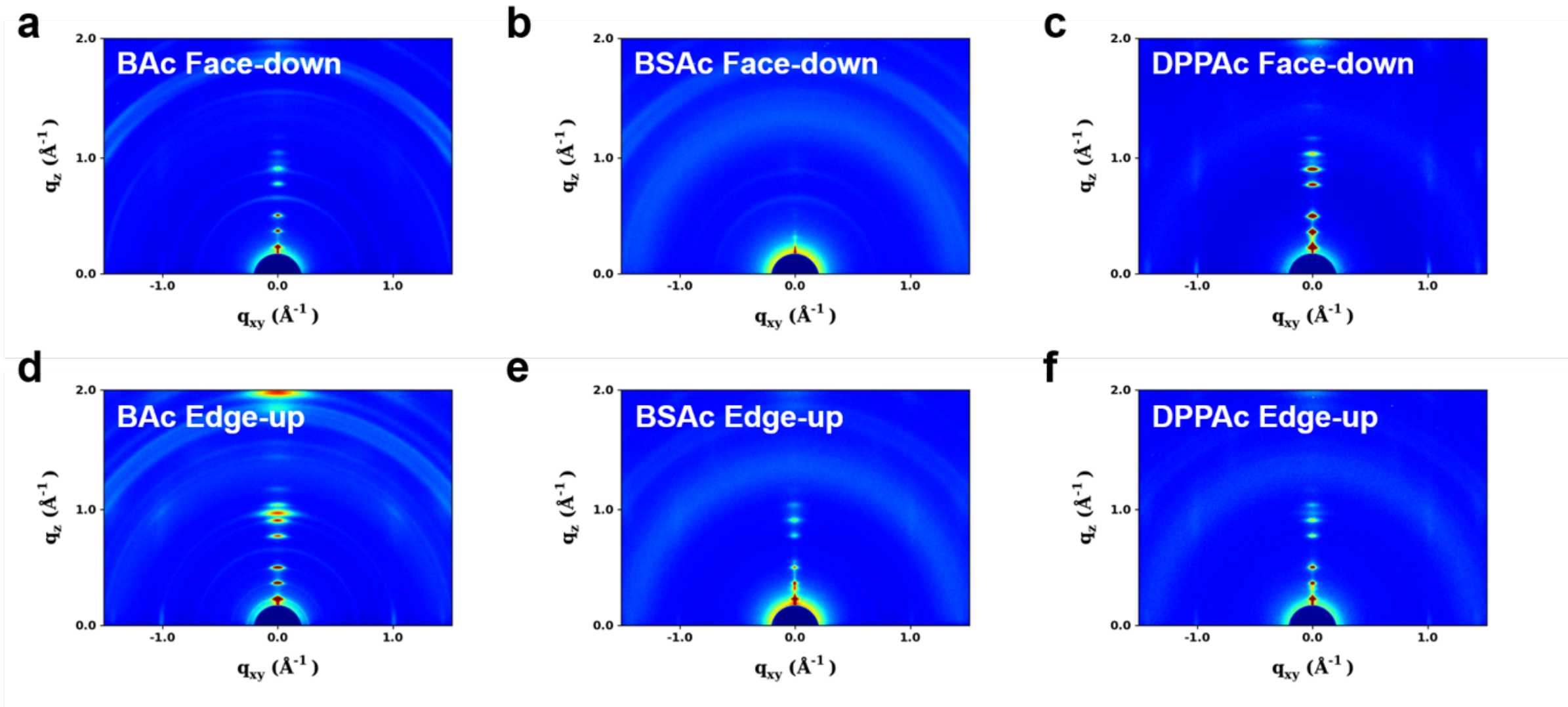

Figure S22. Grazing-incidence wide-angle X-ray scattering (GIWAXS) patterns of BAc-, BSAc-, DPPAc-PeNPL thin films.

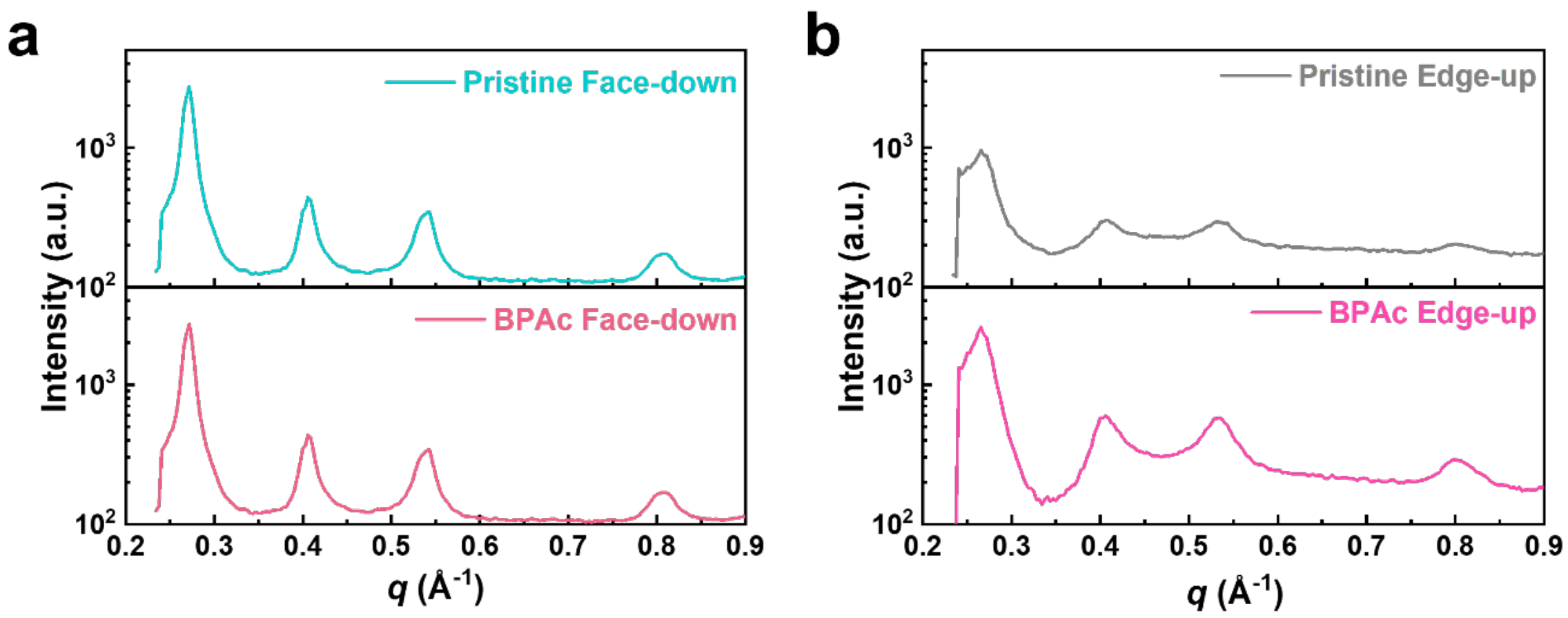

Figure S23. One-dimensional GIWAXS profiles integrated over all azimuthal angles in the low-angle regions for PeNPL films with an overall **a** face-down and **b** edge-up orientation. *q* is the absolute value of the scattering vector.

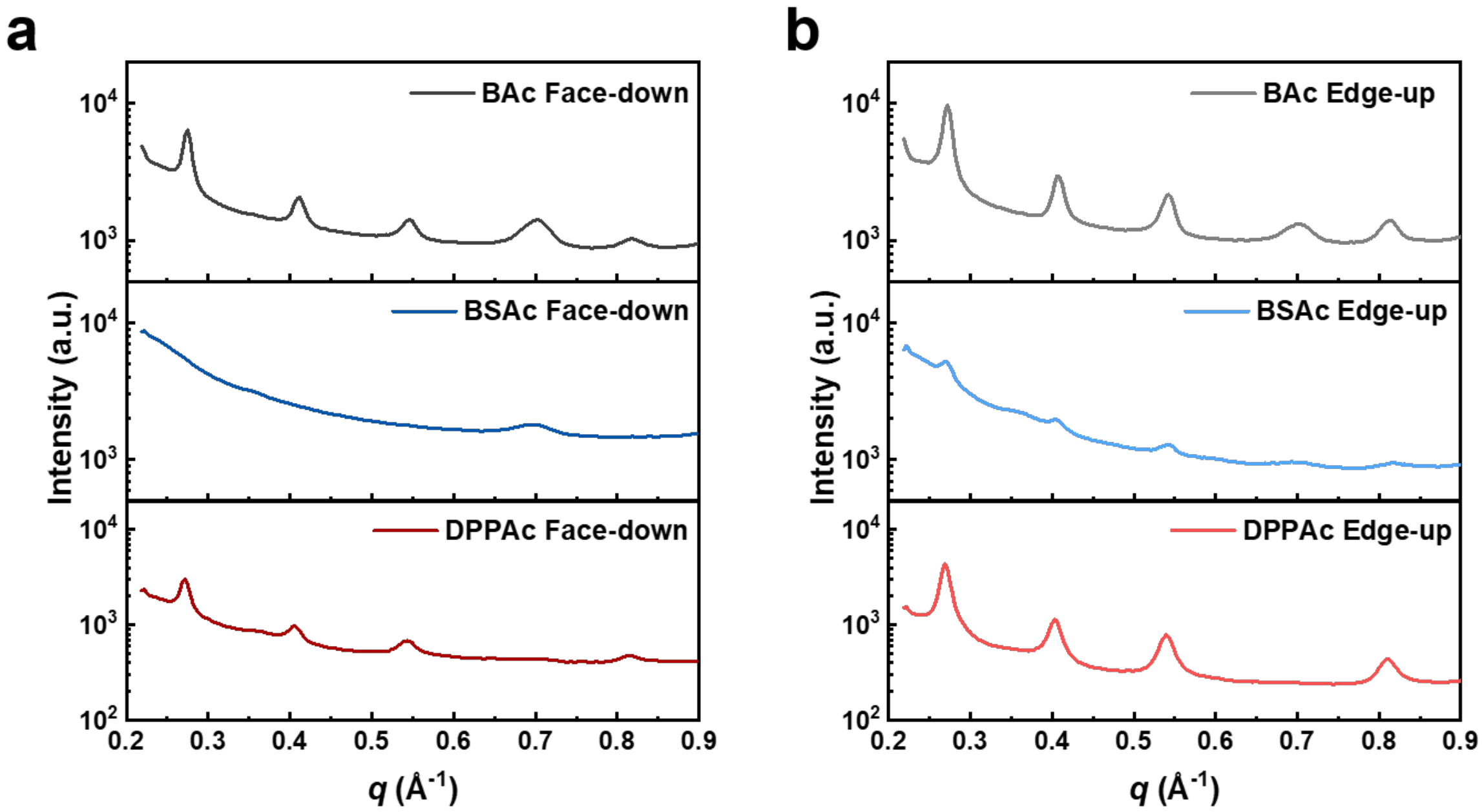

Figure S24. One-dimensional GIWAXS profiles integrated over all azimuthal angles for **a** face-down and **b** edge-up oriented BAc-, BSAc- and DPPAc-PeNPL thin films in low-angle region. *q* is the absolute value of the scattering vector.

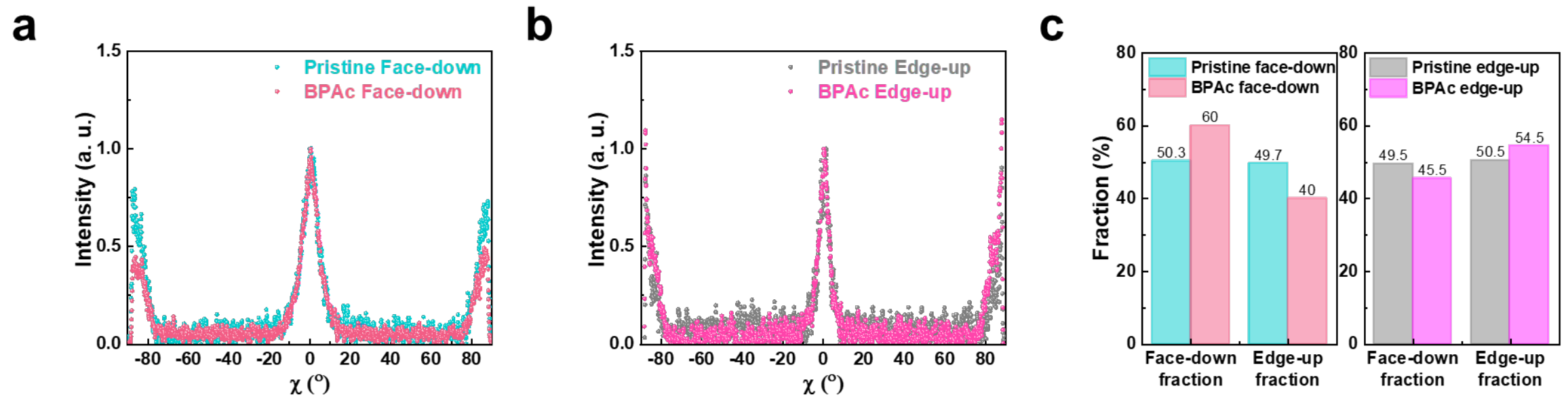

Figure S25. Azimuthal angle (χ) dependence of (100) diffraction peak intensity for **a** face-down and **b** edge-up oriented PeNPL thin films. **c** The extracted ratio of face-down and edge-up oriented PeNPLs from Figure S18a-b. The distribution ratio is calculated by

$$\text{Fraction of face-down oriented PeNPLs (\%)} = \frac{\int_0^{20} \mathrm{I}\, d\chi}{\int_{70}^{88.6} \mathrm{I}\, d\chi + \int_0^{20} \mathrm{I}\, d\chi}$$

$$\text{Fraction of edge-up oriented PeNPLs (\%)} = \frac{\int_{70}^{88.6} \mathrm{I}\, d\chi}{\int_{70}^{88.6} \mathrm{I}\, d\chi + \int_0^{20} \mathrm{I}\, d\chi}$$

, where χ is the azimuthal angle and I is intensity.

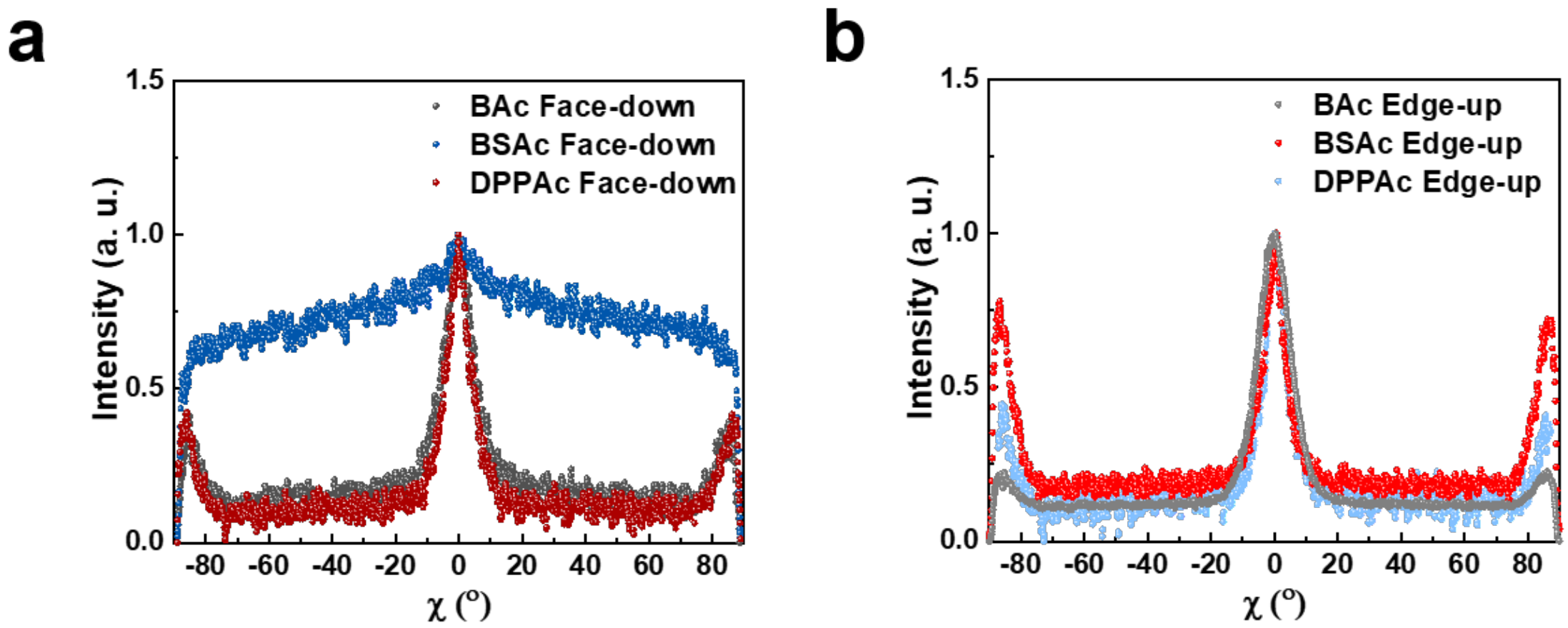

Figure S26. Azimuthal angle (χ) dependence of (100) diffraction peak intensity for **a** face-down and **b** edge-up oriented BAc-, BSAc- and DPPAc-PeNPL thin films.

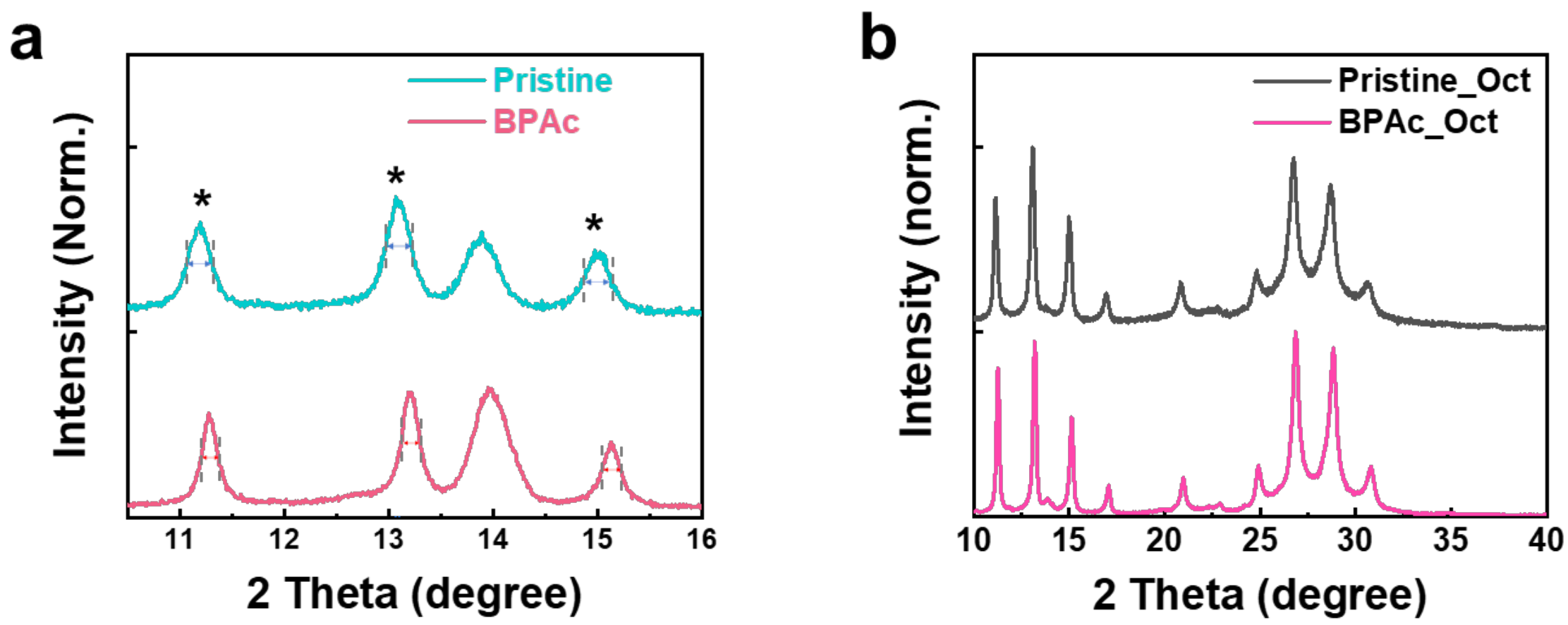

Figure S27. a Zoomed-in X-ray diffraction (XRD) patterns of edge-up oriented each PeNPL thin films. **b** Diffraction (XRD) patterns of face-down oriented each PeNPL thin film. The peaks marked with stars correspond to the multilayer diffraction pattern.

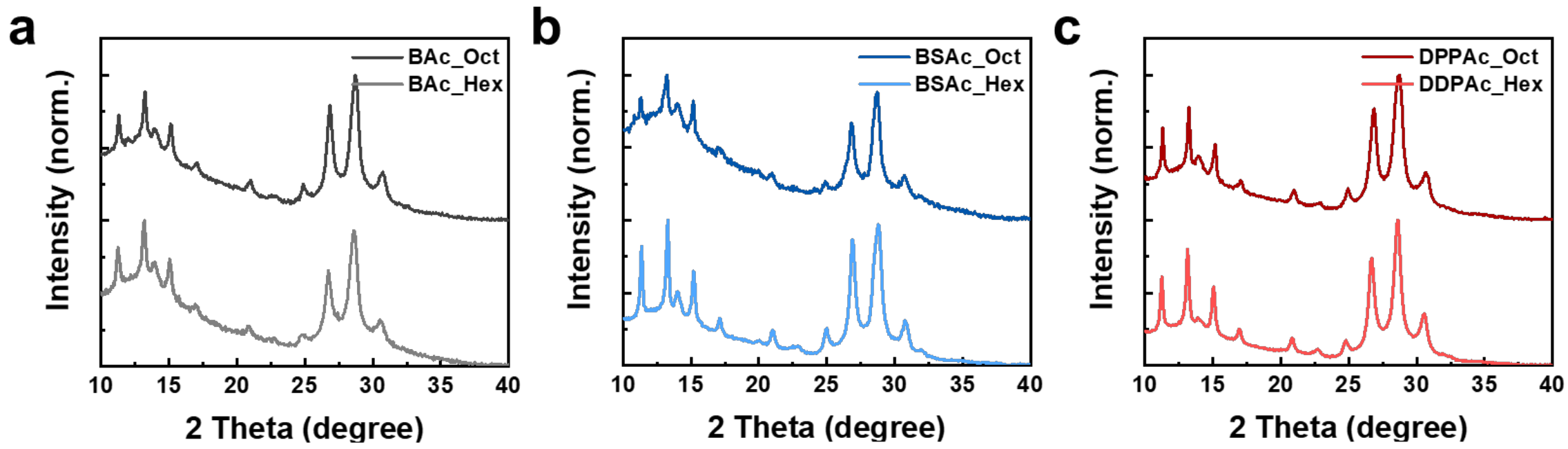

Figure S28. X-ray diffraction (XRD) patterns of **a** BAc-, **b** BSAc- and **c** DPPAc-PeNPL thin film. Each PeNPL thin film was fabricated by spin coating colloidal PeNPL dispersions in octane and hexane.

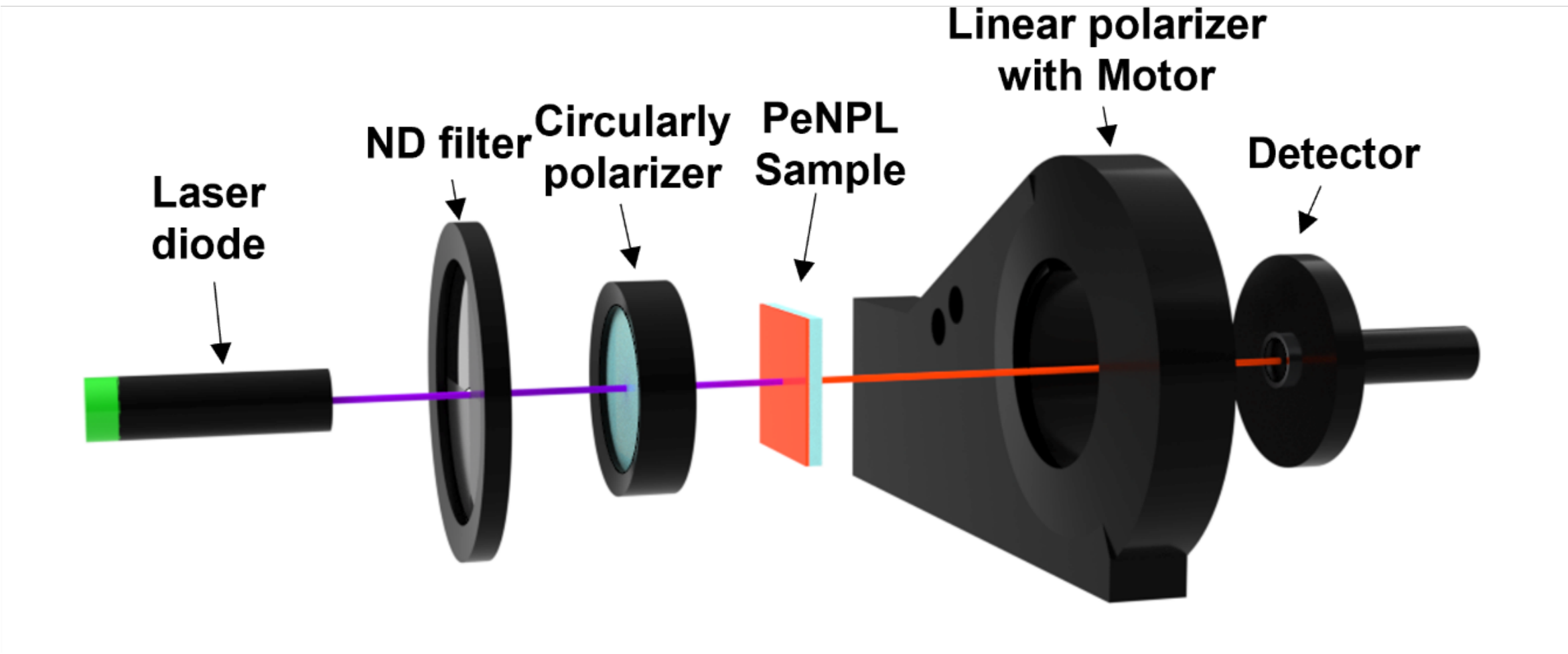

Figure S29. Experimental setup for measuring the degree of linear polarization (DOP) in PL.

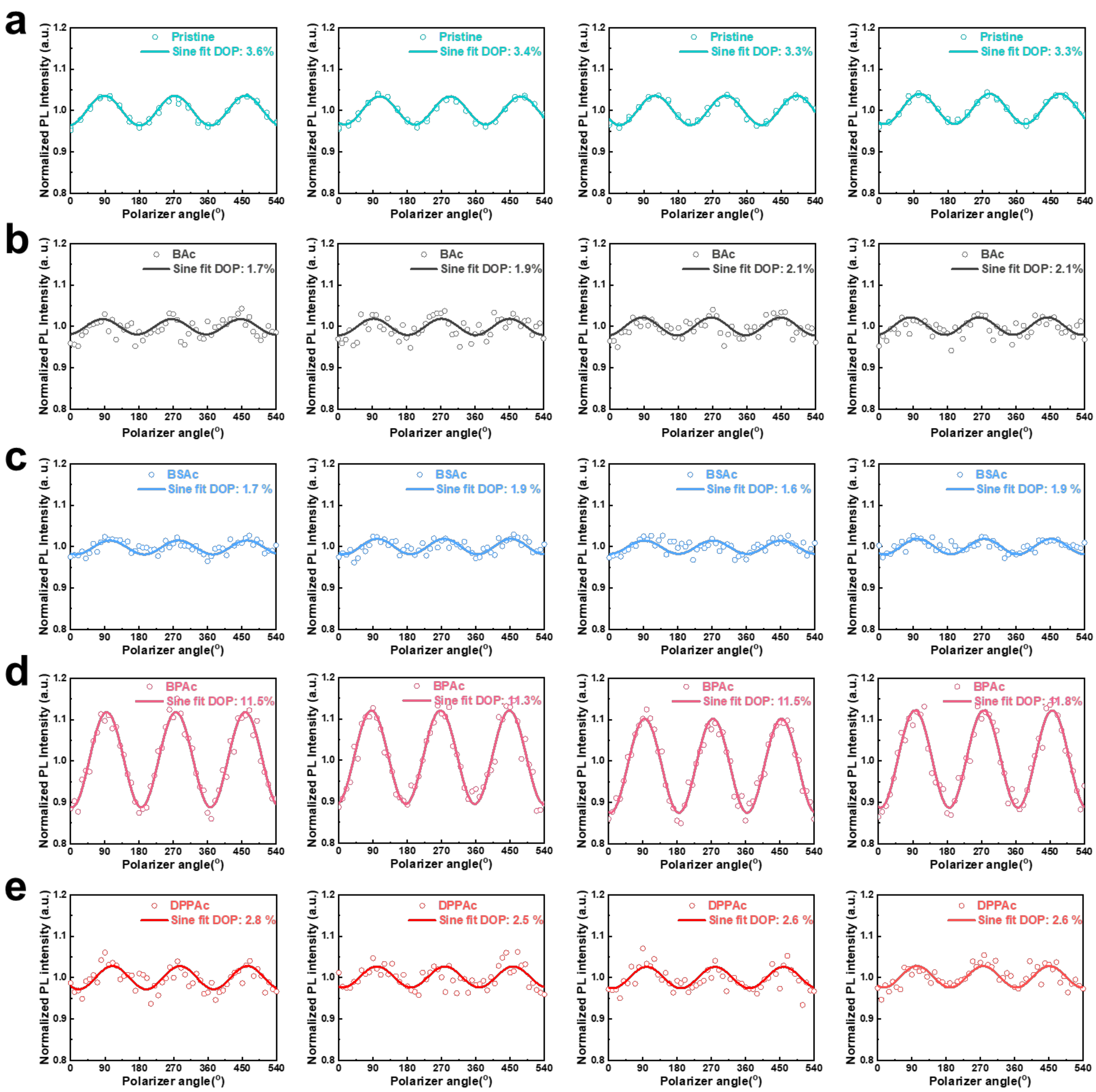

Figure S30. Polarization dependence of the normalized PL intensity of each PeNPLs thin films made in different batches, along with the distribution in the degree of linear polarization (DOP) determined from these measurements. Measurements performed inside a N_2-filled glovebox with a 405 nm wavelength CW laser at 37.3 mW cm^{-2} power density.

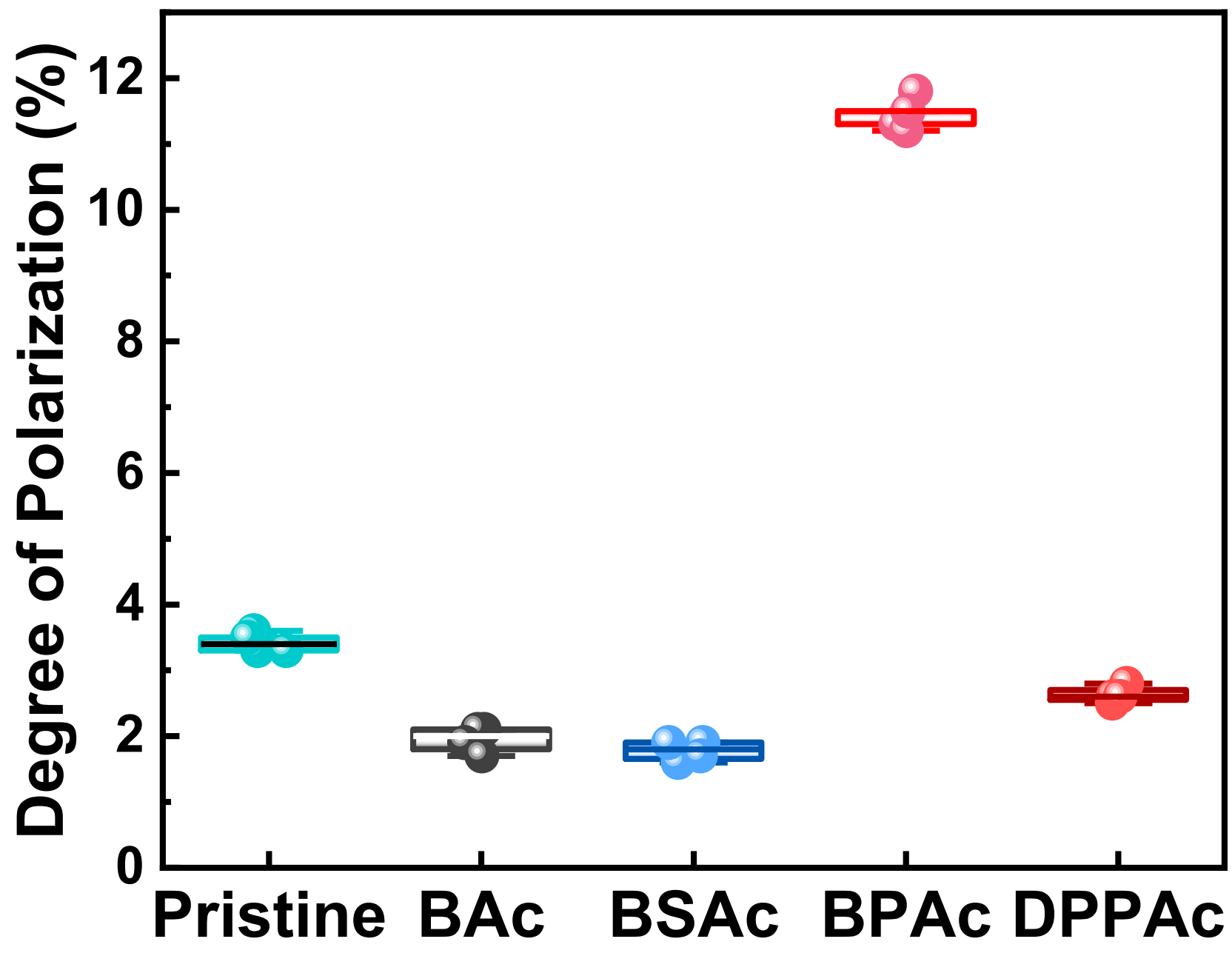

Figure S31. Histogram showing the distribution in the degrees of polarization displayed in Figure S30

Note S3. Optical Simulations of Orientation-Dependent Outcoupling in PeNPL LEDs

To quantitatively estimate the contribution of PeNPL orientation to optical outcoupling, we performed optical simulations using the face-down/edge-up orientation ratios of the PeNPL thin films determined from GIWAXS measurements (Figure S32). The isotropic emitter case was used as a reference, with a relative efficiency of 100%. The vertical dipole ratio was varied from 0.3333, corresponding to isotropic emission, to 0, corresponding to a fully horizontal dipole configuration.

For the pristine-PeNPL film, the face-down/edge-up ratio was measured to be 50.3:49.7, corresponding to a vertical dipole ratio of 0.2485. This orientation distribution yields a modest increase in the calculated relative efficiency compared with the isotropic emitter (Figure S32). For the BPAc-PeNPL film, the face-down/edge-up ratio increased to 60:40, corresponding to a vertical dipole ratio of 0.2, leading to a higher calculated relative efficiency. As an ideal upper-limit case, a fully face-down aligned film with only horizontal dipoles was also simulated, showing a substantially higher relative efficiency. These results indicate that the increased face-down orientation in BPAc-PeNPL films provides an additional optical outcoupling effect.

Together with the improved PLQY, defect passivation, and film uniformity discussed in the main text, this orientation-dependent optical enhancement supports the synergistic origin of the improved EQE in BPAc-PeLEDs.

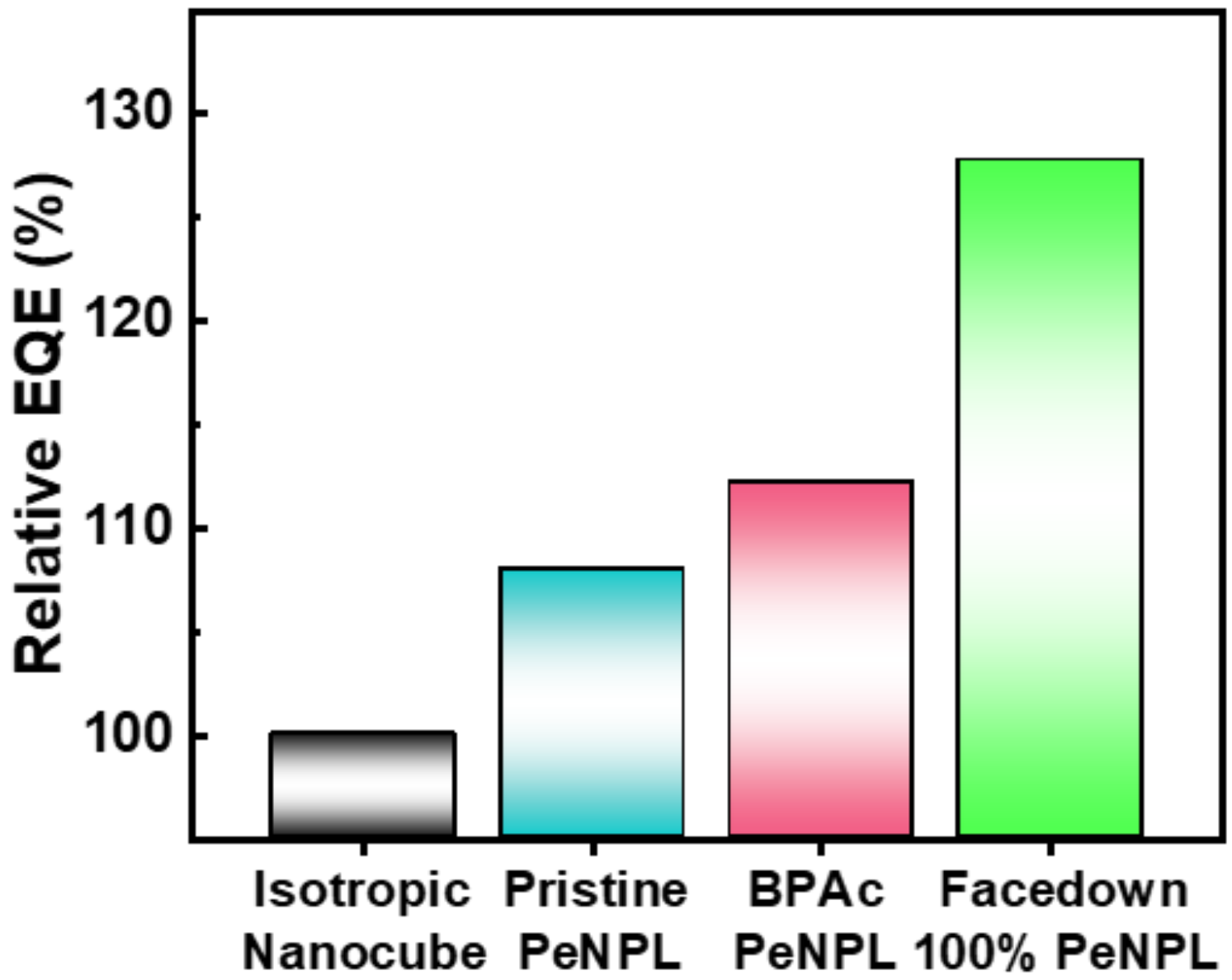

Figure S32. Optical simulation of the relative external quantum efficiency of PeNPLs LED device as a function of vertical dipole ratio. The relative EQE was calculated by varying the vertical dipole ratio from isotropic emission (vertical dipole ratio = 0.3333) to fully horizontal dipole orientation (vertical dipole ratio = 0). The simulated dipole configurations correspond to isotropic orientation, pristine-PeNPL films (face-down:edge-up = 50.3:49.7, vertical dipole ratio = 0.2485), BPAc-PeNPL films (face-down:edge-up = 60:40, vertical dipole ratio = 0.2), and an ideal fully face-down aligned structure with only horizontal dipoles. The simulation indicates that enhanced horizontal dipole orientation increases light outcoupling efficiency, resulting in improved relative EQE.

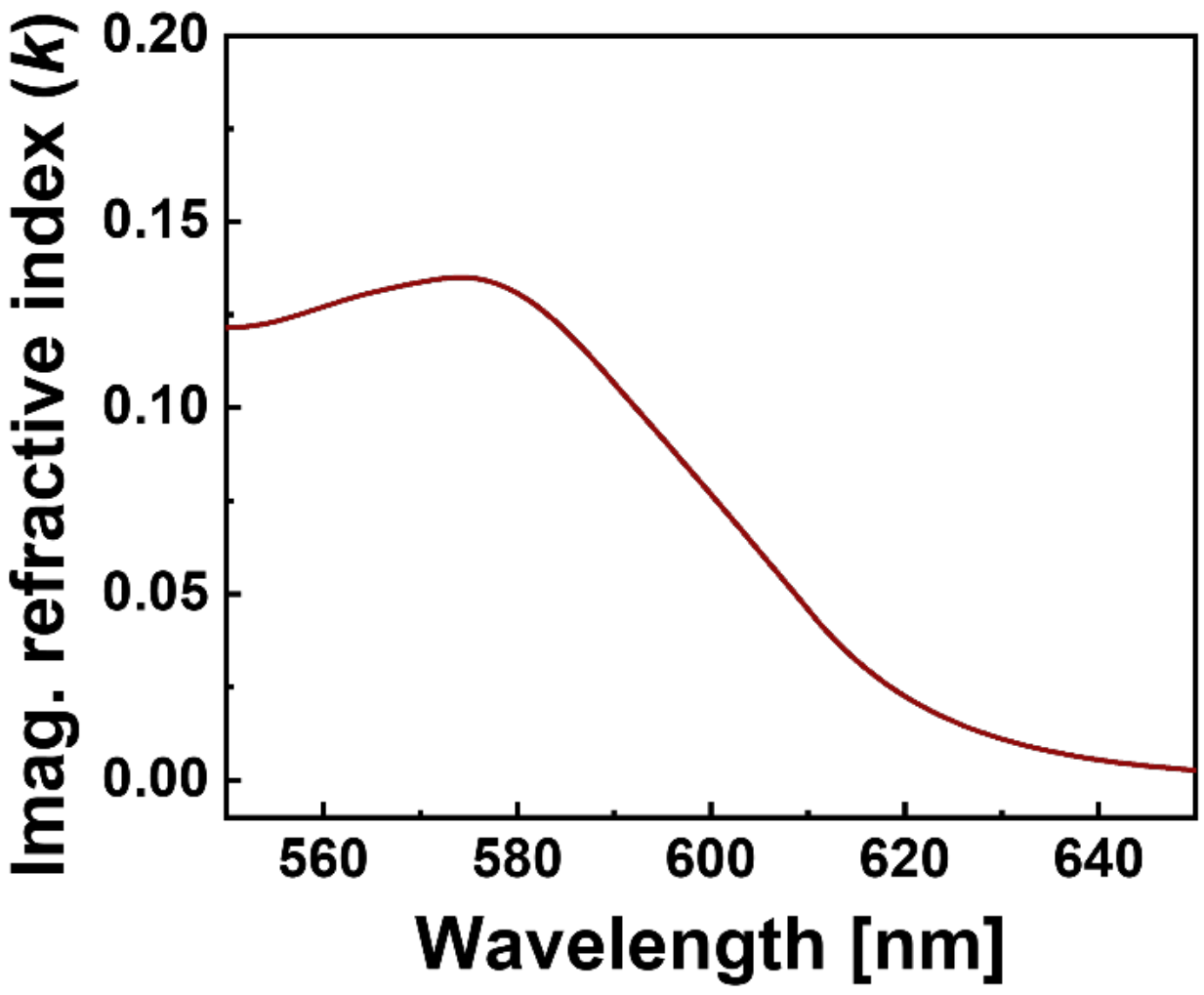

Figure S33. Refractive index of PeNPLs film as a function of wavelength. The imaginary part of the refractive index, k, was calculated from the absorption coefficient of the films. The extinction coefficient k was calculated using the relation $k = \alpha\lambda/4\pi$, where λ is the wavelength.

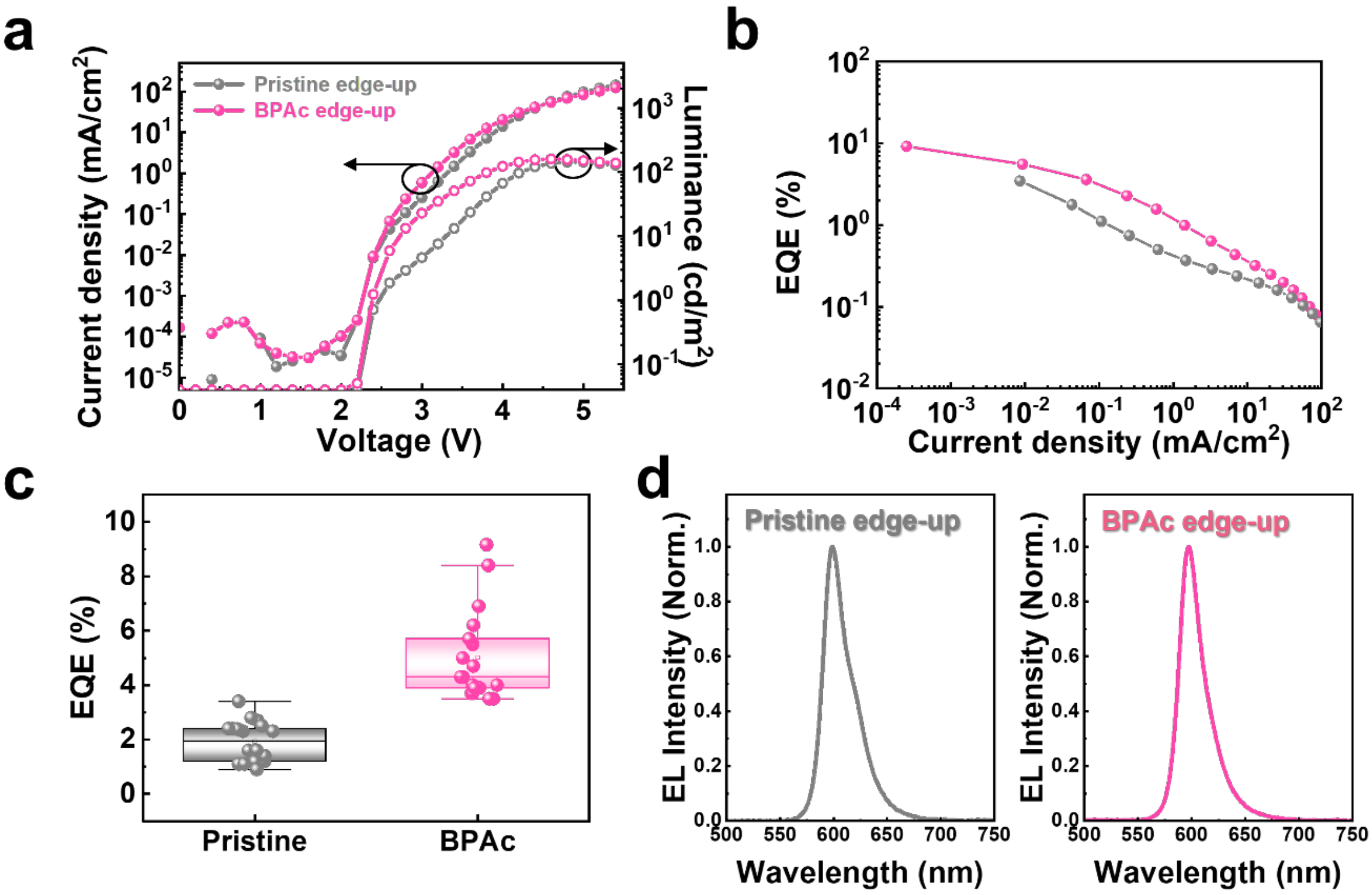

e

	L_{max}(cd/m²)	EQE(%)	Turn-on(V) (0.1 cd/m²)	CE_{max}(cd/A)
Pristine edge-up	142@5.0V	3.4@2.4V	2.4	8.4@2.4V
BPAc edge up	159@4.6V	9.1@2.4V	2.2	20@2.2V

Figure S34. a Current density–Voltage (*J–V–L*) voltage curve, **b** External quantum efficiency (EQE)–Current density (*EQE–J*) curve of pristine- and BPAc-PeNPL LED devices based on PeNPLs that have an overall edge-up orientation. **c** EQE_{max} histogram from 17 individual PeNPL devices. **d** Electroluminescence (EL) spectra of each LED devices. **e** Device performance parameters of best-performing LEDs

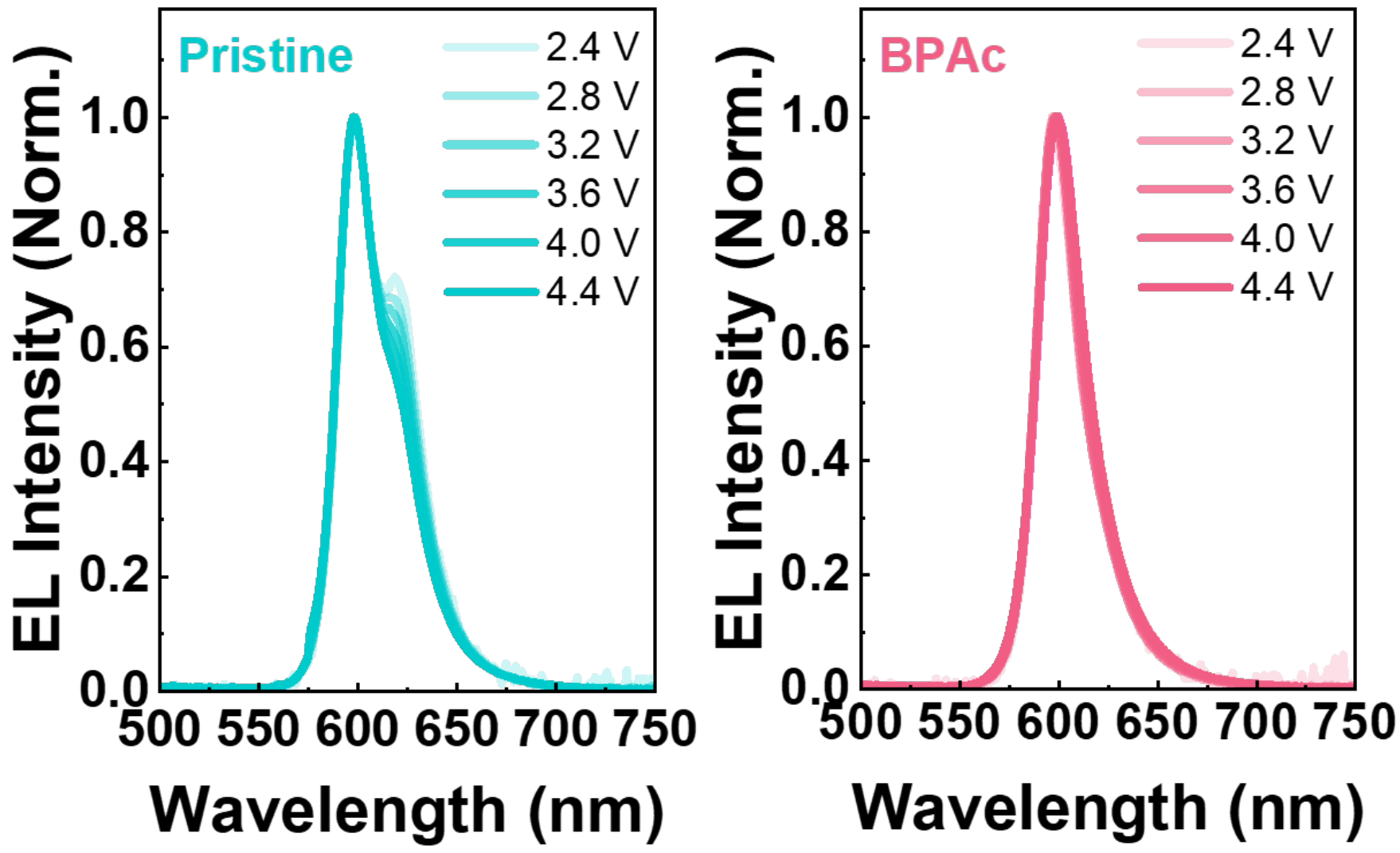

Figure S35. Normalized EL spectra of Pristine- and BPAc-PeLEDs under varying applied voltages. The PeNPLs have an overall face-down orientation.

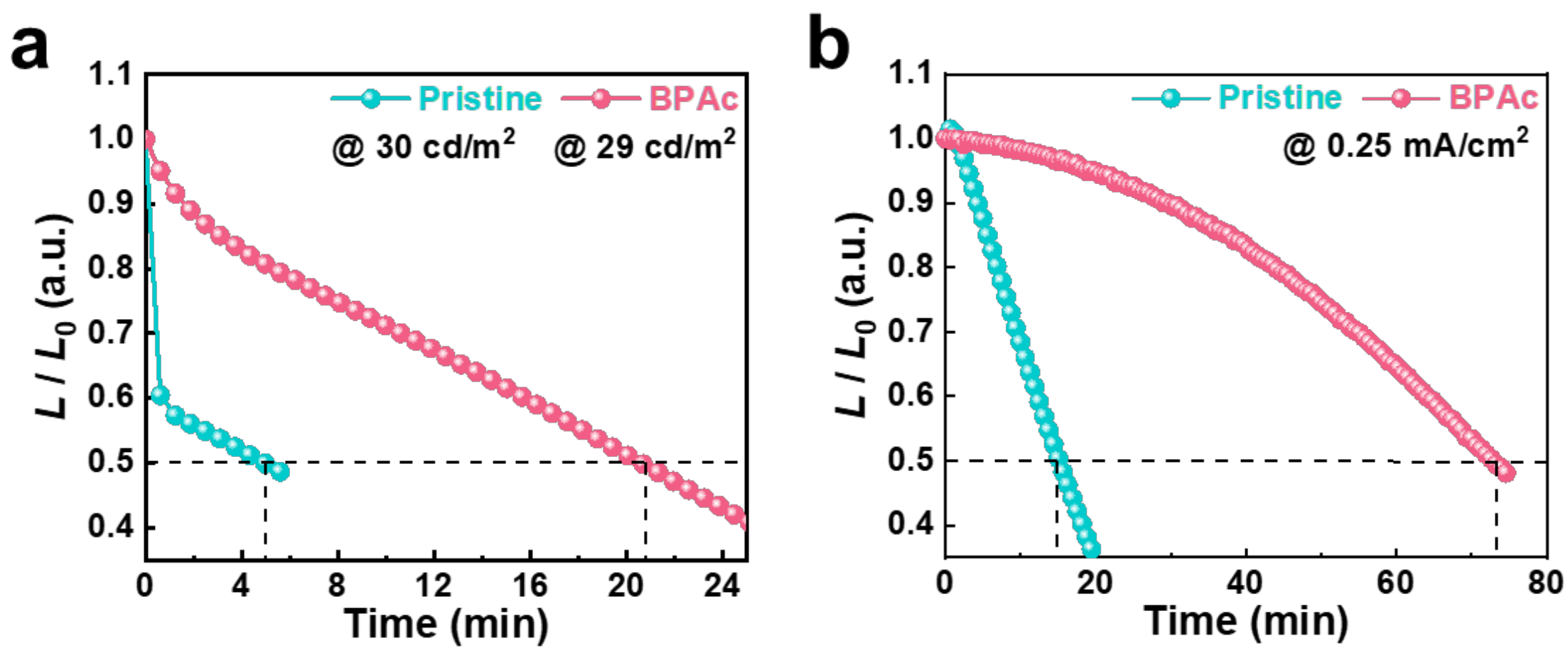

Figure S36. Device stability test from half lifetime measurements (T_{50}) of Pristine- and BPAc-PeLED devices measured under ambient conditions with encapsulation. These PeNPLs have an overall face-down orientation. **a** Pristine-PeLED measured under constant current density (4.44 mA cm^{-2}) and BPAc-PeLED measured under constant current density (0.74 mA cm^{-2}). **b** Pristine-PeLED and BPAc-PeLED measured under constant current density (0.25 mA cm^{-2}, initial luminance: 9.1 cd m^{-2}; pristine-PeLED, 9.9 cd m^{-2}; BPAc-PeLED)

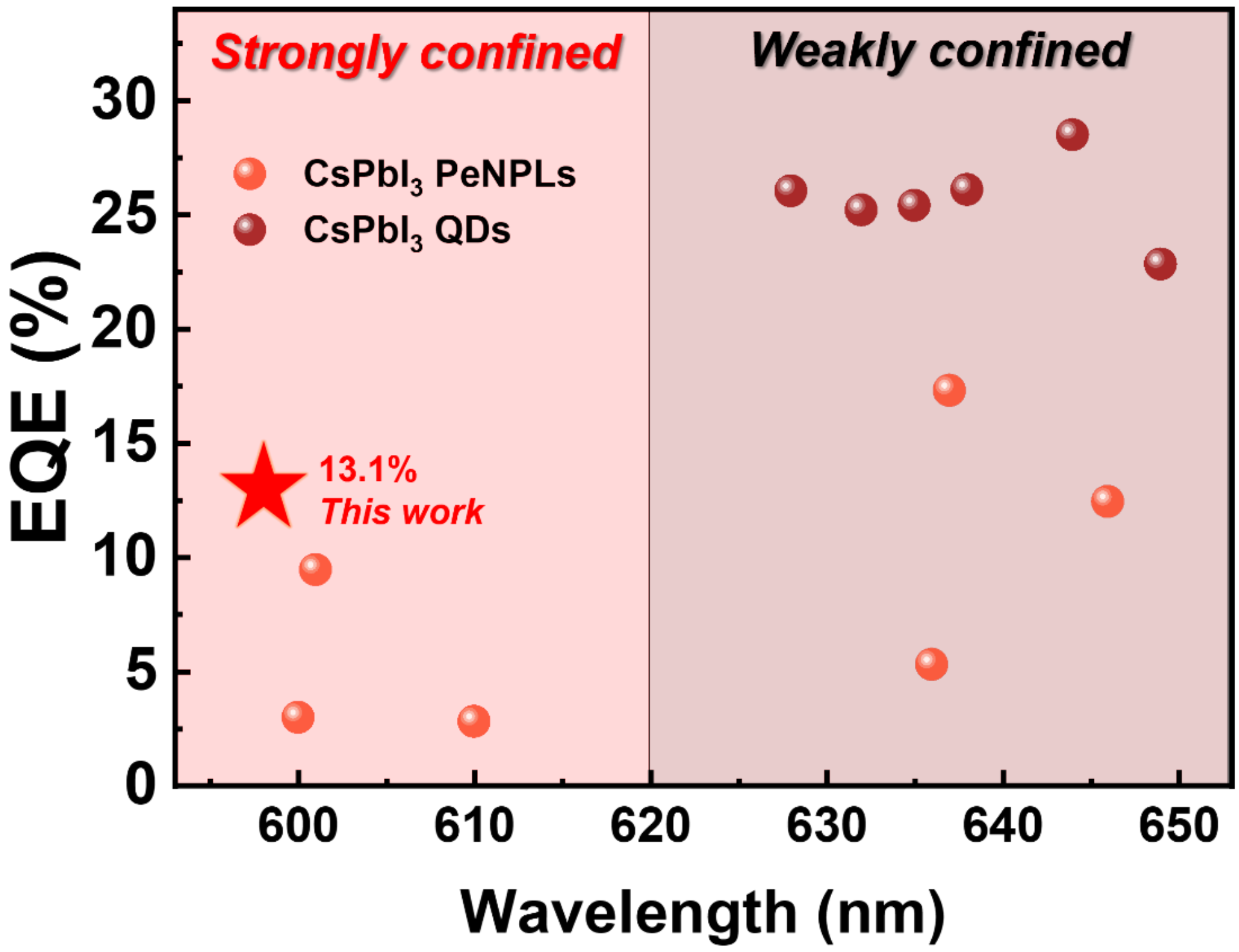

Figure S37. Comparison of the EQE_{max} of $CsPbI_3$ perovskite nanocrystal-based LED devices (< 650 nm) with previous literature [2-14].

Table S1. Summary of time-resolved PL decay profiles of colloidal PeNPL solutions, fit with a phenomenological tri-exponential decay curve. Note that while this model does not necessarily provide a close physical description of the recombination processes involved, it is useful for quantitatively comparing PL decay curves between different samples

Configuration	τ_1 [ns]	τ_2 [ns]	τ_3 [ns]	τ_{avr} [ns]	χ^2
pristine-PeNPLs 8.4 μJ/cm^2	0.44 (15%)	14.61 (62%)	43.39 (23%)	15.89	0.98
pristine-PeNPLs 41.9 μJ/cm^2	0.49 (16%)	13.68 (57%)	36.98 (27%)	15.78	0.99
pristine-PeNPLs 419 μJ/cm^2	0.50 (27%)	13.10 (49%)	37.10 (24%)	14.18	0.99
BPAc-PeNPLs 8.4 μJ/cm^2	14.15 (58%)	43.72 (42%)		22.24	0.98
BPAc-PeNPLs 41.9 μJ/cm^2	14.23 (60%)	45.13 (40%)		21.13	0.98
BPAc-PeNPLs 419 μJ/cm^2	13.62 (58%)	41.81 (42%)		21.82	0.99

Table S2. Summary of superlattices-based perovskite LEDs performance reported in literature.

Type	Perovskite	EL peak [nm]	EQE_{max} [%]	L_{max} [cd/m^2]	Reference
Isotropic nanocrystals (Weakly confined*)	$FA_{0.5}MA_{0.5}PbBr_3$ NCs	528	24.96	5009	*Nat. Comm.* **2022**, 13, 2106
	$CsPbI_3$ NCs	645	15.72	6149	*ACS Energy Lett.* **2025**, 10, 2341-2348
	$CsPb(I_{0.6}Br_{0.4})_3$ NCs	619	10.75	2176	*Chem. Commun.* **2025**, 61, 8859-8862
	$CsPbBr_3$ QDs	518	30.9	117,144	*Nature* **2026**, 652, 1173-1180
Anisotropic nanocrystals (Highly confined*)	$CsPbBr_3$ NPLs (n = 3)	455	4.15	1511	*ACS Energy Lett.* **2023**, 8, 10, 4259–4266
	$CsPbBr_3$ NPLs (n = 3)	460	3.18	128	*ACS Appl. Mater. Interfaces* **2022**, 14, 5682-5691
	$CsPbBr_3$ NPLs (n = 3)	462	1.77	691	*Adv. Energy Mater.* **2023**, 13, 2201605
	$CsPbBr_3$ NPLs (n = 3)	463	3.14	9	*Matter* **2026**, 102760
	$CsPbBr_3$ NPLs (n = 3)	465	5.44	227	*Nano Lett.* **2024**, 24, 7004-7011
	$CsPbBr_3$ NPLs (n = 3)	469	1.42	41	*J. Phys. Chem. C* **2019**, 123, 26161
	$CsPbI_3$ NPLs (n = 3)	601	9.45	29800	*Small* **2025**, 21, 2408422
	$CsPbI_3$ NPLs (n = 3)	620	2.2	408	*Nat. Photon.* **2024**, 18, 586-594
	**$CsPbI_3$ NPLs (n = 3)**	**600**	**13.1**	**78**	**This work**

*Weakly confined: dimensions larger than the exciton Bohr radius; highly confined: at least one dimension smaller than the $n \leq 3$ (ultrathin regime).

SUPPORTING REFERENCES

1. A. Brodzka, P. Kowalczyk, D. Trzepizur, et al., “The Synthesis and Evaluation of Diethyl Benzylphosphonates as Potential Antimicrobial Agents,” *Molecules* 27 (2022): 6865, https://doi.org/10.3390/molecules27206865.

2. J. Kim, Y. I. Kim, H. Guo, et al., “Toward α-CsPbI3 Quantum Dots via Dual-Functional Fluorinated Acidic Ligand,” *ACS Energy Letters* 10 (2025): 4402–4409, https://doi.org/10.1021/acsenergylett.5c01958.

3. Z. Qi, X. Mei, J. Wang, et al., “Ligand-Pinning Induced Size Modulation of CsPbI3 Perovskite Quantum Dots for Red Light-Emitting Diodes,” *Advanced Functional Materials* 34 (2024): 2405679, https://doi.org/10.1002/adfm.202405679.

4. H. Li, Y. Feng, M. Zhu, et al., “Nanosurface-Reconstructed Perovskite for Highly Efficient and Stable Active-Matrix Light-Emitting Diode Display,” *Nature Nanotechnology* 19 (2024): 638–645, https://doi.org/10.1038/s41565-024-01652-y.

5. Y. Feng, H. Li, M. Zhu, et al., “Nucleophilic Reaction-Enabled Chloride Modification on CsPbI3 Quantum Dots for Pure Red Light-Emitting Diodes with Efficiency Exceeding 26%,” *Angewandte Chemie International Edition* 63 (2024): e202318777, https://doi.org/10.1002/anie.202318777.

6. Y. Lou, H. Wang, Z. Zhou, et al., “Stabilization of γ-$CsPbI_3$ Nanoplatelets via Tailored Surface Coordination for High-Performance Rec. 2020 Pure-Red LEDs,” *ACS Nano* 19 (2025): 23151–23161, https://doi.org/10.1021/acsnano.5c04461.

7. A. P. Litvin, Z. Song, X. Zhang, W. Zheng, A. L. Rogach, “Pure-Red Light-Emitting Diodes Based on Lead Halide Perovskite Nanoplatelets Obtained by Ostwald Ripening and Two-Step Anion Exchange,” *Small*, e11548, https://doi.org/10.1002/smll.202511548.

8. M. Chen, T. Zhang, A. Elsukova, et al., “Kinetically Controlled Synthesis of Quasi-Square $CsPbI_3$ Nanoplatelets with Excellent Stability,” *Small* 20 (2024): e2306360, https://doi.org/10.1002/smll.202306360.

9. M. Xie, J. Guo, C. Bi, et al., “Efficient and Stable Pure-Red Perovskite LED Based on Uniform Arrangement Strongly Confined Quantum-Dot Film,” *ACS Energy Letters* 9 (2024): 4003–4008, https://doi.org/10.1021/acsenergylett.4c01479.

10. J. Hu, C. Bi, K. Ren, et al., “High-Efficiency Pure-Red $CsPbI_3$ Quantum Dot Light-Emitting Diodes Enabled by Strongly Electrostatic Potential Solvent and Sequential Ligand Post-Treatment Process,” *Nano Letters* 24 (2024): 4571–4579, https://doi.org/10.1021/acs.nanolett.4c00651.

11. Y. Li, M. Deng, X. Zhang, et al., "Stable and Efficient $CsPbI_3$ Quantum-Dot Light-Emitting Diodes with Strong Quantum Confinement," *Nature Communications* 15 (2024): 5696, https://doi.org/10.1038/s41467-024-50022-8.

12. J. Ye, A. Ren, L. Dai, et al., "Direct Linearly Polarized Electroluminescence from Perovskite Nanoplatelet Superlattices," *Nature Photonics* 18 (2024): 586–594, https://doi.org/10.1038/s41566-024-01398-y.

13. A. P. Litvin, J. Guo, J. Wang, X. Zhang, W. Zheng, A. L. Rogach, "Systematic Study of the Synthesis of Monodisperse CsPbI3 Perovskite Nanoplatelets for Efficient Color-Pure Light Emitting Diodes," *Small (*2025): e2408422, https://doi.org/10.1002/smll.202408422.

14. Z. Li, Y. Song, S. Cao, et al., "Achieving a Near-Unity Photoluminescence Quantum Yield and High Stability of $CsPbI_3$ Nanoplatelets by Hydroiodic Acid-Assisted Ligand Treatment," *Inorganic Chemistry Frontiers* 11 (2024): 2392–2401, https://doi.org/10.1039/d4qi00168k.
